# Crystal structures of ten phosphane chalcogenide complexes of gold(III) chloride and bromide

**DOI:** 10.1107/S2056989024002032

**Published:** 2024-03-12

**Authors:** Daniel Upmann, Dirk Bockfeld, Peter G. Jones, Eliza Târcoveanu

**Affiliations:** a Institut für Anorganische und Analytische Chemie, Technische Universität Braunschweig, Hagenring 30, D-38106 Braunschweig, Germany; Universität Greifswald, Germany

**Keywords:** crystal structure, gold(III) halides, phosphane chalcogenides, secondary inter­actions

## Abstract

The structures of ten phosphane chalcogenide complexes of gold(III) halides, with general formula *R* ^1^_3–*n*_ *R* ^2^_*n*_P*E*Au*X*_3_ (*R* ^1^ = *t*-butyl; *R* ^2^ = *i*-propyl; *n* = 0 to 3; *E* = S or Se; *X* = Cl or Br) are presented. The eight possible chlorido derivatives are: **9a**, *n* = 3, *E* = S; **10a**, *n* = 2, *E* = S; **11a**, *n* = 1, *E* = S; **12a**, *n* = 0, *E* = S; **13a**, *n* = 3, *E* = Se; **14a**, *n* = 2, *E* = Se; **15a**, *n* = 1, *E* = Se; and **16a**, *n* = 0, *E* = Se, and the corresponding bromido derivatives are **9b**–**16b** in the same order. Structures were obtained for **9a**, **10a** (and a second polymorph **10aa**), **11a** (and its deutero­chloro­form monosolvate **11aa**), **12a** (as its di­chloro­methane monosolvate), **14a**, **15a** (as its deutero­chloro­form monosolvate **15aa**, in which the solvent mol­ecule is disordered over two positions), **9b**, **11b**, **13b** and **15b**. The structures of **11a**, **15a**, **11b** and **15b** form an isotypic set, and those of compounds **10aa** and **14a** form an isotypic pair. All structures have *Z*′ = 1. The gold(III) centres show square-planar coordination geometry and the chalcogenide atoms show approximately tetra­hedral angles (except for the very wide angle in **12a**, probably associated with the bulky *t*-butyl groups). The bond lengths at the gold atoms are lengthened with respect to the known gold(I) derivatives, and demonstrate a considerable *trans* influence of S and Se donor atoms on a *trans* Au—Cl bond. Each compound with an isopropyl group shows a short intra­molecular contact of the type C—H_methine_⋯*X_cis_*; these may be regarded as intra­molecular ‘weak’ hydrogen bonds, and they determine the orientation of the Au*X*_3_ groups. The mol­ecular packing is analysed in terms of various short contacts such as weak hydrogen bonds C—H⋯*X* and contacts between the heavier atoms, such as *X*⋯*X* (**9a**, **10aa**, **11aa**, **15aa** and **9b**), S⋯S (**10aa**, **11a** and **12a**) and S⋯Cl (**10a**). The packing of the polymorphs **10a** and **10aa** is thus quite different. The solvent mol­ecules take part in C—H⋯Cl hydrogen bonds; for **15aa**, a disordered solvent region at *z* ≃ 0 is observed. Structure **13b** involves unusual inversion-symmetric dimers with Se⋯Au and Se⋯Br contacts, further connected by Br⋯Br contacts.

## Abstract 2

The structures of ten phosphane chalcogenide complexes of gold(III) halides are presented and compared.

## 1. Chemical context

In Part 6 of this series (Upmann *et al.*, 2024 ▸), we presented the structures of sixteen halogenido-gold(I) complexes of various tri­alkyl­phosphane chalcogenides. Appropriate background material, together with a summary of our previous results, can be found in that publication and is not repeated here.

In this paper, we report the structures of ten tri­alkyl­phosphane chalcogenide complexes of gold(III) trihalides, with general formula (^*t*^Bu_3–*n*_ *^i^* Pr_*n*_P=*E*)Au*X*_3_, of which there are sixteen possible permutations of *n*, the chalcogenide *E* (restricted to S or Se) and *X* (for *X* = Cl or Br; tri­iodido complexes are generally not accessible). The eight theoretically obtainable tri­chlorido derivatives are: **9a**, *n* = 3, *E* = S; **10a**, *n* = 2, *E* = S; **11a**, *n* = 1, *E* = S; **12a**, *n* = 0, *E* = S; **13a**, *n* = 3, *E* = Se; **14a**, *n* = 2, *E* = Se; **15a**, *n* = 1, *E* = Se; and **16a**, *n* = 0, *E* = Se, and the corresponding tri­bromido derivatives are **9b**–**16b** in the same order. These are generally obtained from the gold(I) precursors (numbered analogously as **1a**–**8a** and **1b**–**8b** in our previous publication; Upmann *et al.*, 2024 ▸) using one mole equivalent of elemental bromine or PhICl_2_ [commonly known as iodo­benzene dichloride; systematic name di­chloro­(phen­yl)-λ^3^-iodane] as oxidizing agents. However, ^*t*^Bu_3_P = SeAuCl, **8a**, was found to be unstable, thus ruling out the preparation of **16a**; **13a** also proved to be unstable; and the attempted syntheses of **10b**, **12b**, **14b** and **16b** led to different products, to be described in future publications. This left ten successfully synthesized compounds, leading to thirteen structures; **10a** was obtained as two polymorphs (the second termed **10aa**), whereas structures of **11a** and **15a** were determined both solvent-free and as the deutero­chloro­form monosolvates **11aa** and **15aa**. Compound **12a** was obtained as a di­chloro­methane monosolvate. The structures of **10a**, **11a**, **14a** and **15aa** were briefly presented in a preliminary communication (Upmann & Jones, 2013 ▸), but have been re-refined using a much more recent version of *SHELXL* (2019 rather than 1997; Sheldrick, 2015 ▸) and are discussed in more detail here. Details of the composition of each compound studied are given in Table 1 ▸.

We had earlier synthesized all four permutations (*E* = S or Se, *X* = Cl or Br) of Ph_3_P*E*Au*X*_3_ (Taouss *et al.*, 2015 ▸), but were unable to determine any of the structures because of extensive ‘streaking’ of the diffraction images. 
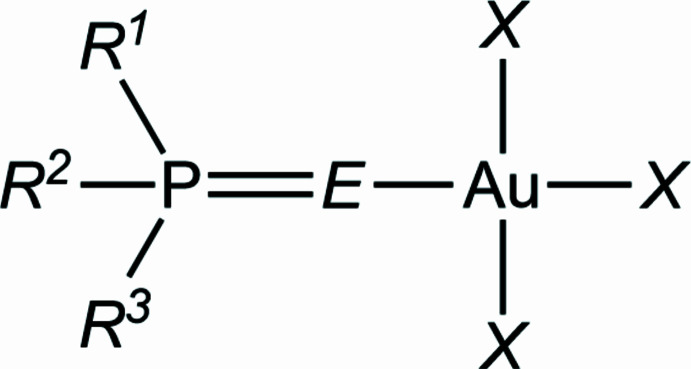


## 2. Structural commentary

**Figure 1:**
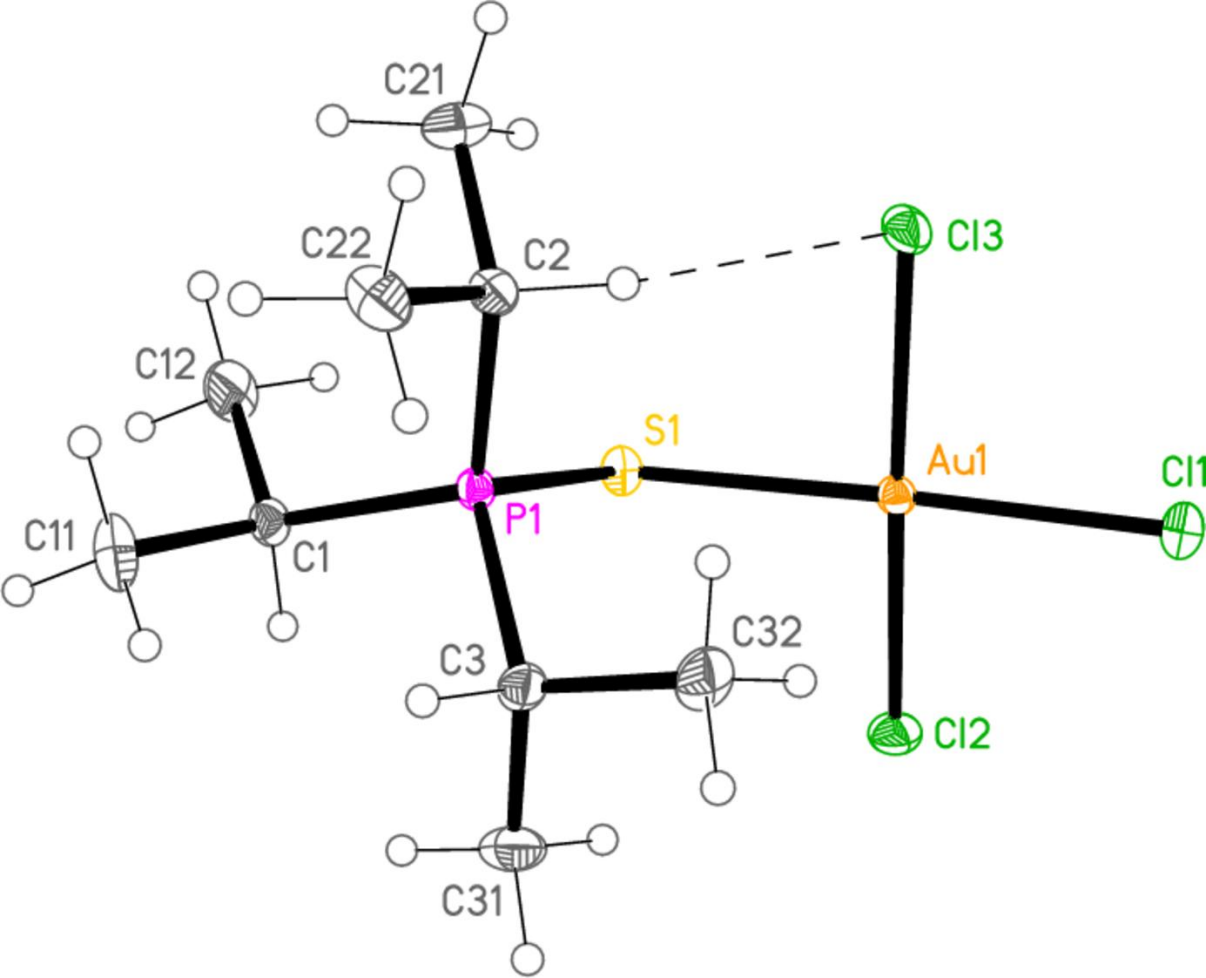
The structure of compound **9a** in the crystal. Ellipsoids represent 50% probability levels. The dashed line represents an intra­molecular hydrogen bond (see text).

*General comments*: All compounds crystallize with one formula unit in the asymmetric unit. The mol­ecular structures are shown in Figs. 1 ▸–13 ▸ ▸ ▸ ▸ ▸ ▸ ▸ ▸ ▸ ▸ ▸ ▸; selected mol­ecular dimensions are given in Tables 2 ▸–14 ▸ ▸ ▸ ▸ ▸ ▸ ▸ ▸ ▸ ▸ ▸ ▸. The *trans* (to *E*) halogen atoms are numbered as *X*1 throughout. All comparisons to the analogous series of gold(I) compounds refer to our previous paper (Upmann *et al.*, 2024 ▸). As expected, all compounds show square planar coordination geometry (angle ranges *ca* 87–95 and 172–179°) at the gold(III) centres; the largest mean deviation from the plane containing the gold atom and all donor atoms is 0.078 Å for **11b**. The approximately tetra­hedral angles (except for **12a**) at the chalcogenide atoms would also be expected (discussed below in more detail).

*Isotypy*: In an extensive series of closely analogous structures, several would be expected to be isotypic. Indeed, the four compounds **11a**, **15a**, **11b** and **15b** form an isotypic set, and compounds **10aa** and **14a** form an isotypic pair.

*Bond lengths and angles (1). P—E—Au—X_3_ groups*: The P—S and P—Se bond lengths lie in the ranges 2.0523–2.0665 (av. 2.0602) and 2.2085–2.2247 (av. 2.2183) Å, respectively, significantly longer than in the gold(I) derivatives (av. 2.0368 and 2.1938 Å, respectively); this further lengthening with respect to the ‘standard’ bond lengths of *ca* 1.95 and 2.11 Å, respectively, in the free ligands implies a slightly higher contribution of the ‘resonance’ form with a P—*E* single bond to the overall bonding. The bond lengths at the gold atoms are in general considerably lengthened with respect to the gold(I) derivatives; the average bond lengths (Å), with the corres­ponding Au^I^ values in square brackets, are Au—S 2.3337 [2.2760], Au—Se 2.4494 [2.3845], Au—Cl *trans* to *E* 2.3107 [2.2840], *cis* to *E* 2.2855, Au—Br *trans* to *E* 2.4471 [2.3979], *cis* to *E* 2.4336.

The considerable *trans* influence of S and Se donor atoms on a *trans* Au—Cl bond is striking. Thus the six Au—Cl bonds *trans* to S have an average length of 2.3054 Å, with twelve shorter *cis* bond lengths, average 2.2857 Å; three Au—Cl bond lengths *trans* to Se have an average length of 2.3213 Å, with six shorter *cis* bond lengths, average 2.2851 Å. However, few other clear trends can be recognized; the Au—S and Au—Se bonds are slightly longer *trans* to Br (av. 2.3445 and 2.4571 Å) than *trans* to Cl (av. 2.3301 and 2.4443 Å), but the differences and the sample sizes are both small. This would be consistent with similar *trans* influences for S, Se and Br.

The angles P—S—Au lie in the range 106.70–111.96 (av. 110.29°), with P—Se—Au = 107.24–108.49 (av. 108.08°). The angles are appreciably wider than for the Au^I^ derivatives (av. 106.17 and 103.86°). Here we have, however, excluded the extreme outlier **12a**, with a P—S—Au angle of 117.50 (3)°, which we tentatively attribute to steric effects; **12a** is the only ^*t*^Bu_3_P=*E* derivative reported in this paper (see also Section 4).

*Bond lengths and angles (2). Phosphane chalcogenide ligands*: For the Au^I^ derivatives involving both types of alkyl groups, the carbon atom anti­periplanar to Au across the Au—*E*—P—C sequence generally belongs to an *i*-propyl group (the exceptions are the ^*t*^Bu_2_^*i*^PrP=*E* derivatives **3a** and **3b**). For the Au^III^ derivatives, however, all six structures involving such ligands have an anti­periplanar *t*-butyl group. Because of the bulky alkyl substituents at phospho­rus, most C—P—C bond angles are greater than the ideal tetra­hedral value. As compensation for this, the *E*—P—C angles to the carbon atom anti­periplanar to *E* are narrower, with values in the range 99.3–102.7°. The steric crowding is also reflected in several short intra­molecular contacts involving the hydrogen atoms. These are listed for convenience in the tables of hydrogen bonds. In particular, contacts of the type C—H⋯*X_cis_*, invariably involving a methine hydrogen (except for the ^*t*^Bu_3_P=S derivative **12a**, which has no methine hydrogens), are short enough to be regarded as intra­molecular ‘weak’ hydrogen bonds; we suggest that the formation of these hydrogen bonds overrides the tendency for the *i*-propyl group to adopt the anti­periplanar position. The halogen atoms are numbered such that *X*3 is the intra­molecular hydrogen-bond acceptor. This hydrogen bond is drawn explicitly only for compound **9a** (Fig. 1 ▸), although the C—H group pointing towards *X*3 can easily be recognized for several other compounds. Another effect associated with these hydrogen bonds is the consistent positioning of the Au*X*_3_ group, with *X*—Au—*E*—P torsion angles of *ca* 65° for the hydrogen-bonded *X* atom and *ca* 115° (with the opposite sign) for the other *cis X*; again, the ^*t*^Bu_3_P=S derivative **12a** behaves differently, with *X*—Au—*E*—P torsion angles of 82.19 (3) and −100.28 (3)°. The C—H⋯Au contacts (the latter with H⋯Au as short as 2.68 Å) all involve methyl groups and could be regarded as an inevitable consequence of the crowding effects rather than any significant inter­action. This applies *a fortiori* to the short C—H⋯*E* contacts, which have very narrow angles at the hydrogen atom.

For the compounds with two structure determinations, least-squares fits were performed (for non-hydrogen atoms) using the program *XP* (Bruker, 1998 ▸). The r.m.s. deviations were 0.20 Å for **10a**/**10aa** (0.10 Å if methyl carbon atoms were omitted; Fig. 14 ▸), 0.13 Å for **11a**/**11aa** and 0.05 Å for **15a**/**15aa** (the latter were fitted with one structure inverted).

*Mol­ecular volumes*: For the gold(I) species, the change in mol­ecular volume (cell volume/*Z*) on changing the elements *E* or *X* (for the same phosphine) was calculated for six pairs S/Se and for six pairs Cl/Br. The values thus obtained were generally consistent with the atomic volumes calculated by Hofmann (2002 ▸), namely S 25.5, Se 30.3, Cl 25.8 and Br 32.7 Å^3^. For the gold(III) series, fewer pairs are available, but the results are less convincing. The two polymorphs **10a**/**10aa** already differ by 4.4 Å^3^, which is comparable to the difference in volume between S and Se according to the density rule postulated by Kitaigorodskii (1961 ▸). More ‘rational’ (denser) crystal packing should correspond to a more stable polymorph, so that **10aa** (*D*_x_ = 2.073 Mg m^−3^) should be more stable than **10a** (*D*_x_ = 2.051 Mg m^−3^). For three pairs Cl/Br, the volume increases range from 13 to 20 Å^3^, but for four pairs S/Se, the differences range from 1.5 to 7 Å^3^ and for the pair **9b**/**13b** the difference has the wrong sign (the sulfur-containing **9b** has a slightly larger volume, by 1 Å^3^). Clearly such a simple additive model for the mol­ecular volumes does not apply well here.

## 3. Supra­molecular features

For general aspects of packing and types of secondary inter­action, as applied to these compounds, a series of general articles are quoted in our previous paper (Upmann *et al.*, 2024 ▸). Hydrogen bonds are given in Tables 15 ▸–27 ▸ ▸ ▸ ▸ ▸ ▸ ▸ ▸ ▸ ▸ ▸ ▸; these include intra­molecular contacts (see above) and several borderline cases. The corresponding symmetry operators, not given explicitly in the following discussion, may also be found in those Tables. In all packing diagrams presented here, hydrogen atoms not involved in hydrogen bonding are omitted for clarity, and the atom labels indicate the asymmetric unit. It is worth repeating the caveat that X-ray methods reveal short inter­molecular contacts, but not the corresponding energies, so that descriptions of mol­ecular packing in terms of particular secondary contacts must to some extent be subjective. Similarly, there is no clear objective judgement, on the basis of contact lengths and angles, as to which contacts should be regarded as more important or less important for the packing. Finally, the exposed nature of the one-coordinate halogen atoms, combined with the large number of hydrogen atoms, means that some short H⋯*X* contacts are inevitable. Nevertheless, it is possible to obtain informative packing diagrams.

For compound **9a**, five H⋯Cl contacts (from H1, H2, H3, H22*A* and H11*C*) combine to form a layer structure parallel to (011) (Fig. 15 ▸). A short Cl3⋯Cl3 contact of 3.625 (1) Å, operator 1 − *x*, −*y*, 1 − *z*, is also observed. We have previously noted the tendency of tetra­halogenidoaurate(III) anions to display short *X*⋯*X* contacts (Döring & Jones, 2016 ▸), and these are also observed in some neutral trihalogenidogold(III) complexes such as tri­bromido­(piperidine)­gold(III) (Döring & Jones, 2023 ▸).

Compound **10a** also forms a layer structure, parallel to the *ac* plane, involving three H⋯Cl contacts (from H13*B*, H13*C* and H32*A*) and one S⋯Cl contact [S1⋯Cl3(1 − *x*, 1 − *y*, 1 − *z*) = 3.6746 (9) Å] (Fig. 16 ▸). The second polymorph **10aa** has a completely different packing; there are no H⋯Cl contacts < 2.94 Å, but instead the mol­ecules associate to form dimers (Fig. 17 ▸) with a short S1⋯S1 contact of 3.622 (2) Å (operator 1 − *x*, −*y*, 1 − *z*). The corresponding S1⋯Au1 distance is 4.0282 (8) Å, and there is a borderline H22*C*⋯Au1 contact of 3.18 Å. Dimers are linked to form a chain parallel to the *a* axis by the contact Cl2⋯Cl3 3.5885 (11) Å (operator −1 + *x*, *y*, *z*; Fig. 18 ▸). The packing of **11a** is similar to that of **10a**, again involving inversion-symmetric dimers [S1⋯S1 = 3.4257 (9), S1⋯Au1 = 4.0467 (5), H21*C*⋯Au1 = 3.03 Å, operator 1 − *x*, 1 − *y*, −*z*], but with these being linked by the contact H13⋯Cl2 to form chains parallel to the *c* axis (Fig. 19 ▸). Compound **14a** (isotypic to **10aa**) has contact distances Se1⋯Se1′ = 3.615 (2) and Cl2⋯Cl3′ = 3.511 (3) Å. Compounds **11b**, **15a** and **15b** (isotypic to **11a**) have distances Se1⋯Se1′ = 3.3447 (4), S1⋯S1′ = 3.4513 (11) and Se1⋯Se1′ = 3.3734 (5) Å, respectively. The primes indicate the operators given above for the parent structures.

For compound **11aa**, which is the deutero­chloro­form solvate of **11a**, the solvent mol­ecule is well-ordered, and its deuterium atom is involved in a three-centre hydrogen bond to Cl1 and Cl2. The residues are further linked by the short contact Cl3⋯Cl6 = 3.6706 (7) Å (operator *x*, −1 + *y*, *z*), forming chains parallel to the *b* axis (Fig. 20 ▸). There are no H⋯Cl contacts < 2.91 Å.

**Figure 6:**
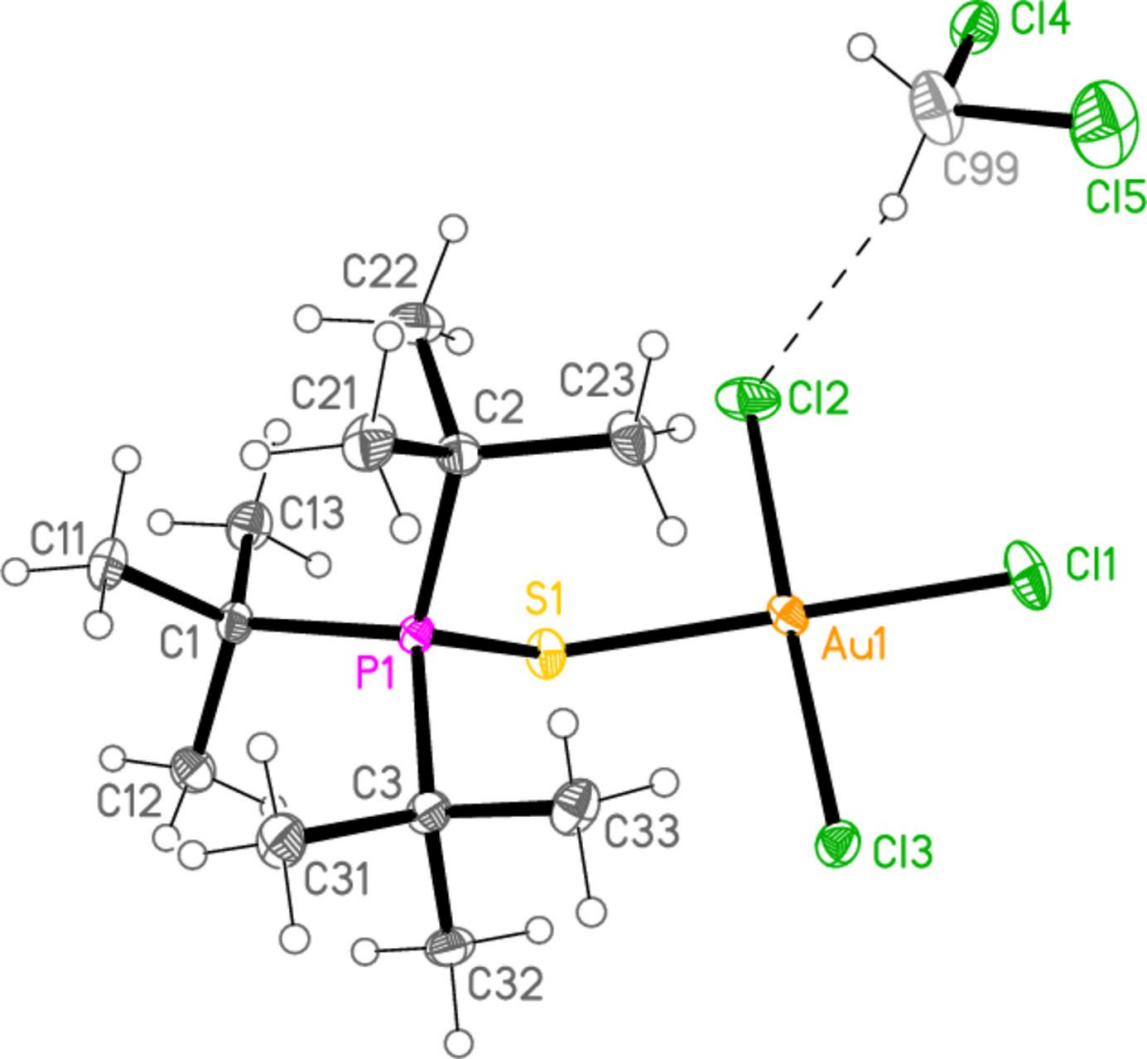
The structure of compound **12a** in the crystal. Ellipsoids represent 50% probability levels. The dashed line represents a ‘weak’ hydrogen bond.

Compound **12a**, which is a di­chloro­methane solvate, has a three-dimensional packing in which the most striking feature is the formation of dimers *via* the contact S1⋯S1(1 − *x*, 1 − *y*, 1 − *z*) = 3.4357 (10) Å. The packing involves layers parallel to (011); these include the solvent contacts H99*A*⋯Cl2 (also shown in Fig. 6 ▸) = 2.84, H99*B*⋯Cl3 = 2.96 and Cl5⋯Cl5(2 − *x*, 2 − *y*, −*z*) = 3.3990 (14) Å (Fig. 21 ▸). The alkyl groups of one layer project into the gaps of neighbouring layers. The contacts H22*A*⋯Cl3 are not shown in Fig. 21 ▸.

Compound **15aa**, the deutero­chloro­form solvate of **15a**, has few short contacts between the mol­ecules of the gold complex itself; the contact Cl1⋯Cl1(−*x*, 2 − *y*, 1 − *z*) = 3.5208 (13) Å links the mol­ecules into simple dimers. Instead it is the disordered solvent, occupying the region at z ≃ 0, that lies between and thus connects the mol­ecules of the gold complex. Fig. 22 ▸ shows this pattern for the major disorder component, with D99⋯Cl2 = 2.76, Au1⋯Cl5 = 3.547 (2) and Cl2⋯Cl5 = 3.603 (2) Å (both −*x*, 2 − *y*, −*z*) and Cl6⋯Cl6 = 3.546 (6) Å (−*x*, 1 − *y*, −*z*). The minor component, somewhat displaced from its major counterpart [C99⋯C99′ = 0.59 (1) Å, angle between C—D vectors = 9°], makes a similar series of contacts, which we do not discuss explicitly.

Compound **9b** has a short Br1⋯Br3(−*x*, 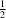 + *y*, 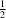 − *z*) contact of 3.7110 (4) Å. This combines with four H⋯Br contacts and one H⋯Au contact to form a layer structure parallel to (10 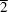 ) (Fig. 23 ▸).

The packing of compound **13b** involves the formation of striking inversion-symmetric dimers, with short contacts Au1⋯Se1′ = 3.7472 (5) and Se1⋯Br3′ = 3.4874 (6) Å, *via* the operator 1 − *x*, 1 − *y*, −*z* (Fig. 24 ▸). The corresponding Au1⋯Au1′ and Au1⋯Br2′ distances of 4.1897 (3) and 4.0038 (5) Å are probably too long to represent any significant inter­action. The dimer formation is reminiscent of the stacking of Au*X*_3_ moieties, as observed for example for the infinite stacks in four polymorphs of tri­chlorido­(tetra­hydro­thio­phene)­gold(III) (Upmann & Jones, 2017 ▸), but with the important difference that the Se atom of **13b** is also involved. The dimers are linked by a short Br2⋯Br3 contact of 3.5478 Å (operator 1 + *x*, *y*, *z*), forming a chain parallel to the *a* axis (Fig. 25 ▸).

## 4. Database survey

The searches employed the routine ConQuest (Bruno *et al.*, 2002 ▸), part of Version 2022.3.0 of the Cambridge Structural Database (Groom *et al.*, 2016 ▸).

A search for all structures containing the moiety *R*_3_P=S—*TM* (coordination numbers of 4 for P and 2 for S, bond orders unspecified, *TM* = any transition metal), excluding any structure in which the P=S or S—*TM* bonds were involved in rings, gave 83 hits. The 108 bond angles at sulfur ranged from 95.6–127.9°, so that this angle is clearly highly variable. The largest value of 127.88 (2)° was observed for the only structure involving ^*t*^Bu_3_P=S, namely [(^*t*^Bu_3_PS)Fe(CO)_2_Cp][PF_6_] (RIDJUK; Kuckmann *et al.*, 2007 ▸), *cf*. structure **12a** above. Similarly, 39 hits for *R*_3_P=Se—*TM* were registered, with 58 angles at selenium in the range 92.1–113.3°. One of the smallest angles, 92.91 (12)°, was observed for (9-phenanthr­yl)Ph_2_PSeAuCl (as its benzene solvate); the analogous sulfur derivative (solvent-free) had a P—S—Au angle of 100.85 (6)° (DUGSAB & DUGFOC; Breshears *et al.*, 2015 ▸).

Searches for other compounds of the type (*R*_3_P=*E)*Au*X*_3_ gave only our own structures, *i.e.* all four permutations (*E* = S or Se, *X* = Cl or Br) of (PCP)^*i*^Pr_2_ *E*Au*X*_3_ (PCP = [2.2]para­cyclo­phanyl; Upmann *et al.*, 2019 ▸).

## 5. Synthesis and crystallization

For several of the compounds, the syntheses can be found in the PhD thesis of D. Upmann (Upmann, 2015 ▸). The following do not appear there:

Compound **9a**. 125 mg (0.3 mmol) of ^*i*^Pr_3_PSAuCl were dissolved in 5 mL of di­chloro­methane, and a solution of iodo­benzene dichloride (82 mg, 0.3 mmol) in 5 mL of di­chloro­methane was added. The red solution was stirred for 30 min. The solvent was removed under vacuum. The product, a red solid, was precipitated with *n*-pentane and dried under vacuum. The yield was not recorded. ^31^P-NMR (200 MHz, CDCl_3_, 300 K): δ (ppm) 78.64 (*s*). Elemental analysis (%): calculated: C 21.81, H 4.27, S 6.47; found: C 22.06, H 4.07, S 6.26. Single crystals were obtained by liquid diffusion of *n*-pentane into a solution of **9a** in di­chloro­methane. Similar attempts to synthesize the selenium analogue (which would have been compound **13a**) were unsuccessful; the product was always an intra­ctable gum that decomposed.

Compound **9b**. 187.4 mg (0.399 mmol) of ^*i*^Pr_3_PSAuBr were dissolved in 3 mL of di­chloro­methane, and 4.16 mL of a 0.096 *M* solution of bromine in di­chloro­methane were added. The product, a red solid, was precipitated with *n*-pentane and dried under vacuum. Yield: 132.6 mg (0.211 mmol, 53%). ^31^P-NMR (81 MHz, CDCl_3_, 300 K): δ (ppm) 77.17 (*s*). Elemental analysis (%): calculated: C 17.19, H 3.37, S 5.10; found: C 17.42, H 3.40, S 5.31. Single crystals were obtained by liquid diffusion of *n*-pentane into a solution of **9b** in di­chloro­methane.

Compound **11b**. 336 mg (0.675 mmol) of ^*i*^Pr_2_^*t*^BuPSAuBr were dissolved in 3 mL of di­chloro­methane, and 6.7 mL of a 0.1 *M* solution of bromine in di­chloro­methane were added. The solution was overlayered with *n*-pentane and stored in a refrigerator (278 K) overnight. Crystals suitable for structure determination formed. After removal of the solvent under vacuum, the product was recrystallized from a mixture of di­chloro­methane and *n*-pentane as a dark-red solid. Yield: 341 mg (0.685 mmol, qu­anti­tative). ^31^P-NMR (81 MHz, CDCl_3_, 300 K): δ (ppm) 85.18 (*s*). Elemental analysis (%): calculated: C 20.11, H 3.84, S 4.88; found: C 20.98, H 4.01, S 4.97.

Compound **12a** was synthesized by the same general method as the other chloro derivatives (*e.g.* **9a**, see above), but the details have unfortunately been lost.

Compound **13b**. 194.7 mg (0.377 mmol) of ^*i*^Pr_3_PSeAuBr were dissolved in 3 mL of di­chloro­methane, and 3.93 mL of a 0.096 *M* solution of bromine in di­chloro­methane were added. The product, a red solid, was precipitated with *n*-pentane and dried under vacuum. Yield: 141.2 mg (0.209 mmol, 55%). ^31^P-NMR (81 MHz, CDCl_3_, 300 K): δ (ppm) 74.46 (*s*, ^1^ *J*_P–Se_ = 520 Hz). Single crystals were obtained by liquid diffusion of *n*-pentane into a solution of **13b** in di­chloro­methane. Elemental analysis (%): calculated: C 15.99, H 3.13; found: C 16.22, H 3.18.

Compound **15b**. 303 mg (0.557 mmol) of ^*i*^Pr^*t*^Bu_2_PSeAuBr were dissolved in 3 mL of di­chloro­methane, and 5.6 mL of a 0.1 *M* solution of bromine in di­chloro­methane were added. The solution was overlayered with *n*-pentane and stored in a refrigerator (278 K) overnight. After removal of the solvent under vacuum, the product was recrystallized twice from a mixture of di­chloro­methane and *n*-pentane as a dark red solid, from which a crystal was selected for measurement. The yield was only *ca* 20%, and neither the elemental analyses nor the ^31^P-NMR results were satisfactory (despite the successful structure determination). We suspect partial decomposition of the product.

The conditions under which the polymorph **10aa** arose were unfortunately not recorded. Crystals of the deutero­chloro­form solvates **11aa** and **15aa** were obtained fortuitously by evaporation from the corresponding NMR solutions.

## 6. Refinement

Details of the measurements and refinements are given in Table 28 ▸. Structures were refined anisotropically on *F* ^2^. Methine and methyl­ene hydrogens were included at calculated positions and refined using a riding model with C—H = 1.00 or 0.99 Å respectively and *U*_iso_(H) = 1.2 × *U*_eq_(C). Methyl groups were refined, using the command AFIX 137, as idealized rigid groups allowed to rotate but not tip, with C—H = 0.98 Å, H—C—H = 109.5° and *U*_iso_(H) = 1.5 × *U*_eq_(C). The use of this command determines the initial hydrogen positions (before refinement) by analysis of maxima in the residual electron density at suitable C—H distances, and these peaks may not be entirely reliable in the presence of a very heavy atom (although in general the refinement seemed to proceed satisfactorily), so that any postulated hydrogen bonds involving methyl hydrogen atoms should be inter­preted with caution.

**Figure 7:**
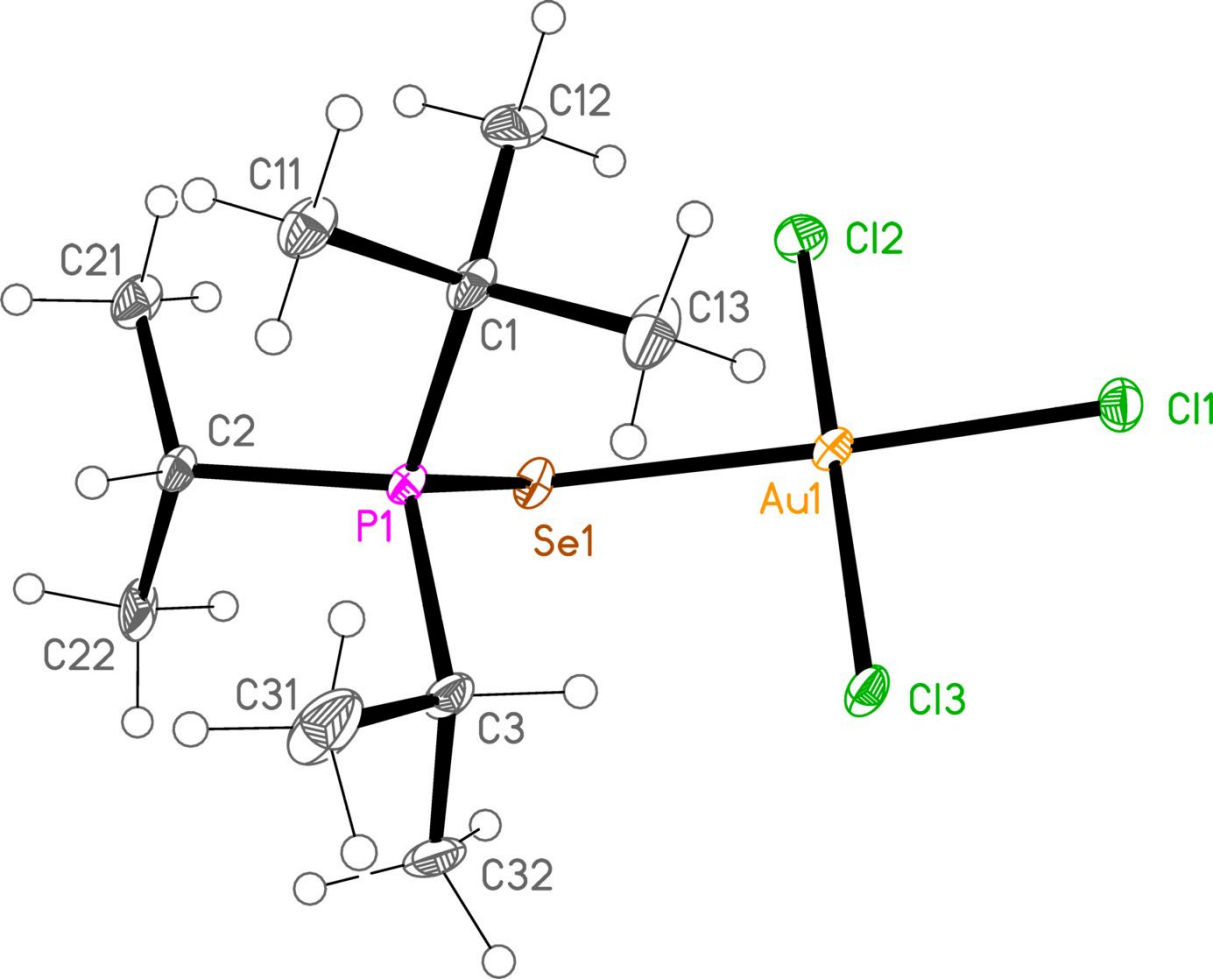
The structure of compound **14a** in the crystal. Ellipsoids represent 30% probability levels.

**Figure 14:**
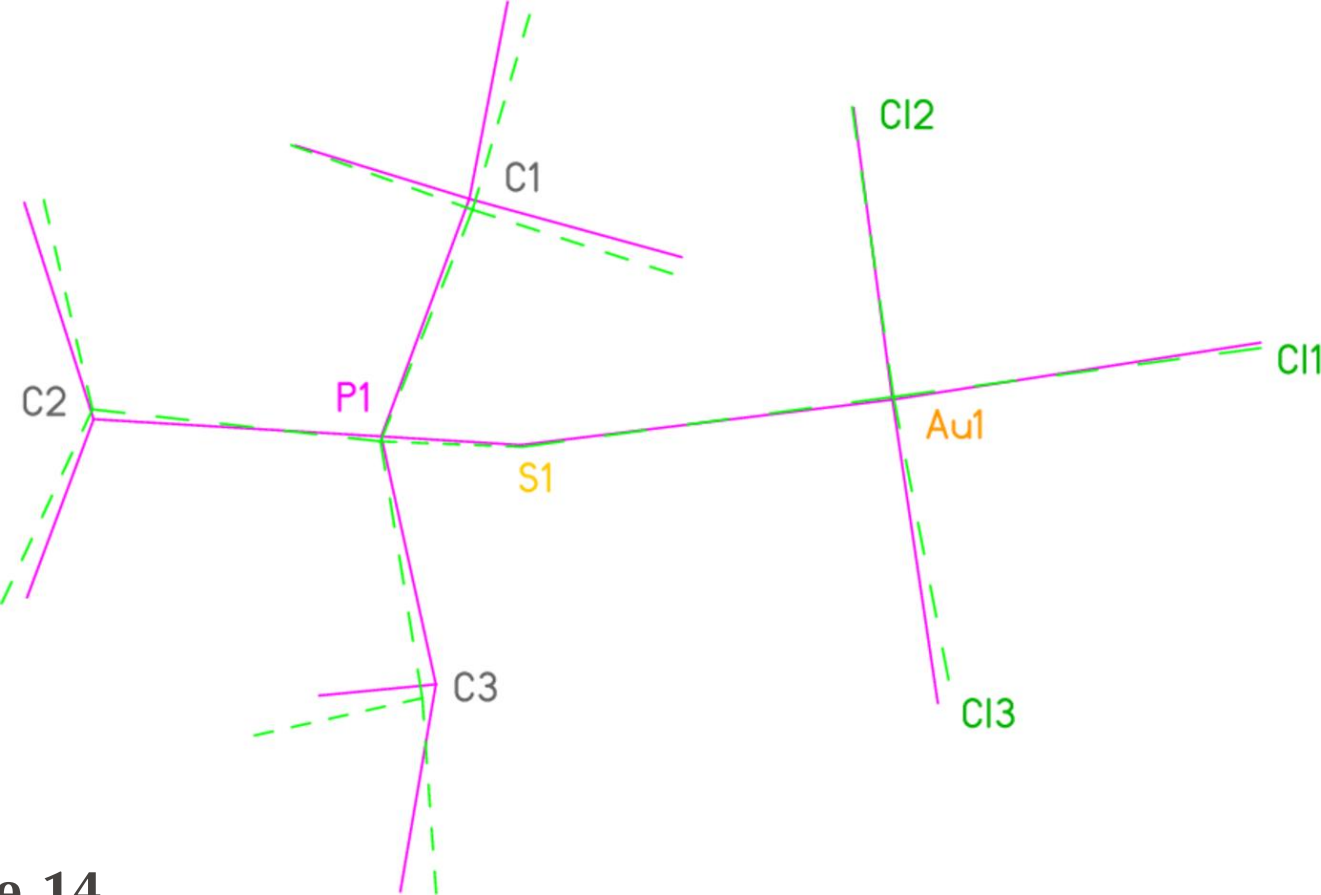
A least-squares fit of structures **10a**/**10aa** (the latter with dashed bonds). The fitted atoms (which exclude the methyl carbons) are labelled. The r.m.s. deviation is 0.10 Å for all fitted atoms (max. 0.23 Å for Cl3).

**Figure 15:**
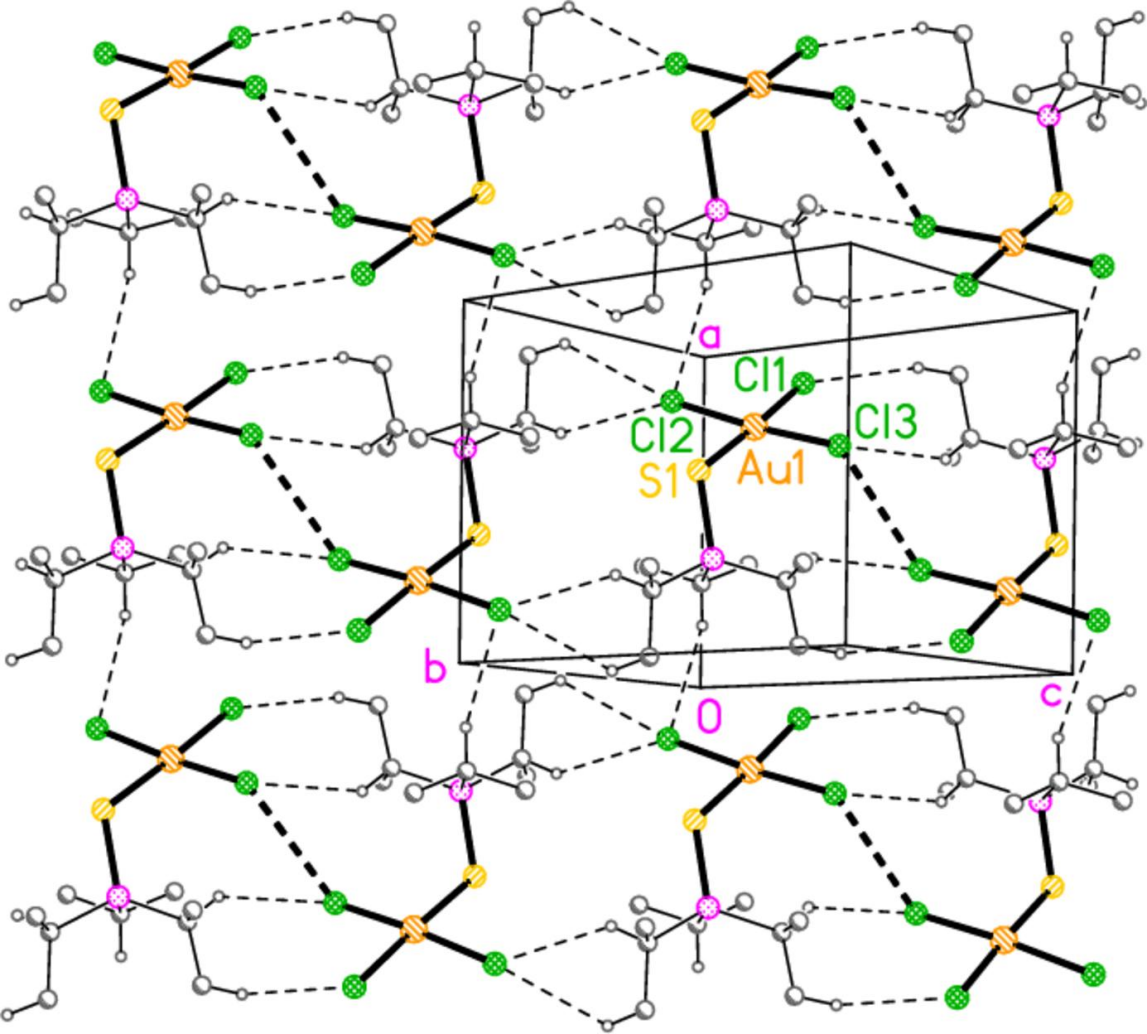
Packing diagram of **9a**, showing the formation of a layer structure parallel to (011) in the region *y* ≃ 0.25, *z* ≃ 0.25. Dashed lines indicate H⋯Cl contacts (thin) or Cl⋯Cl contacts (thick).

**Figure 16:**
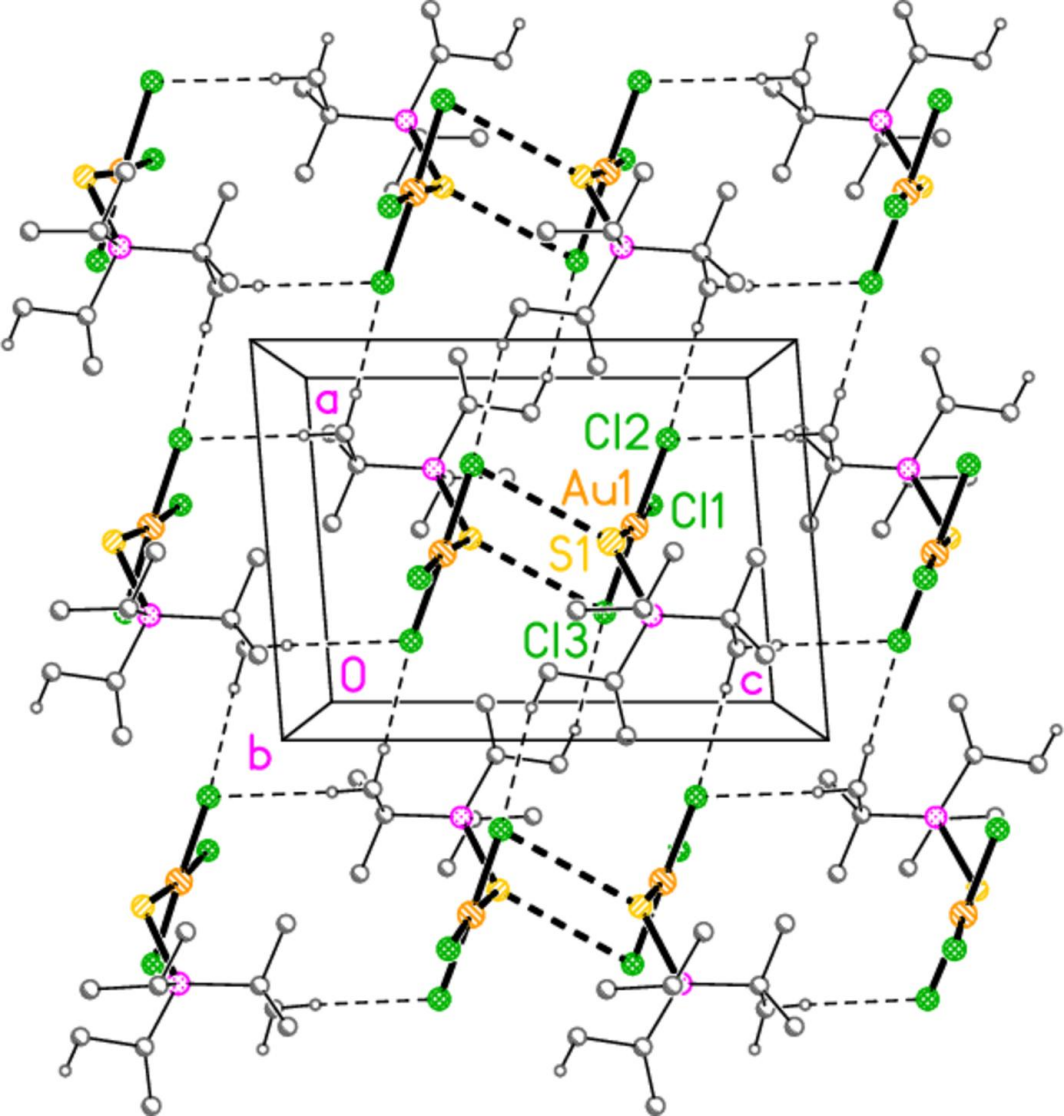
Packing diagram of **10a** viewed parallel to the *b* axis in the region *y* ≃ 0.5. Dashed lines indicate H⋯Cl contacts (thin) or S⋯Cl contacts (thick).

**Figure 17:**
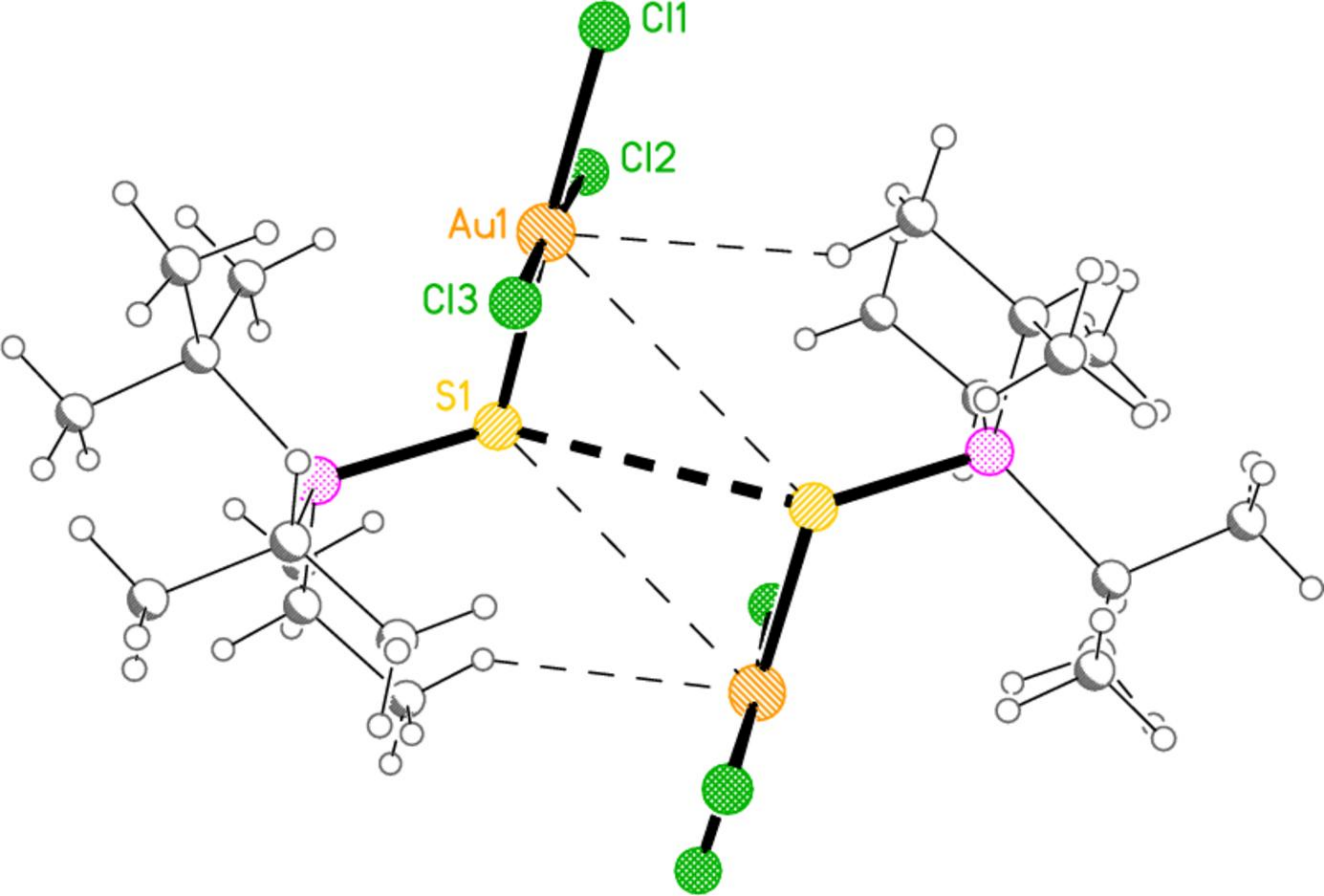
The inversion-symmetric dimer of **10aa**. The short S1⋯S1 contact is shown by the thick dashed line. Thin dashed lines indicate the borderline contacts S1⋯Au1 and H22*C*⋯Au1.

**Figure 18:**
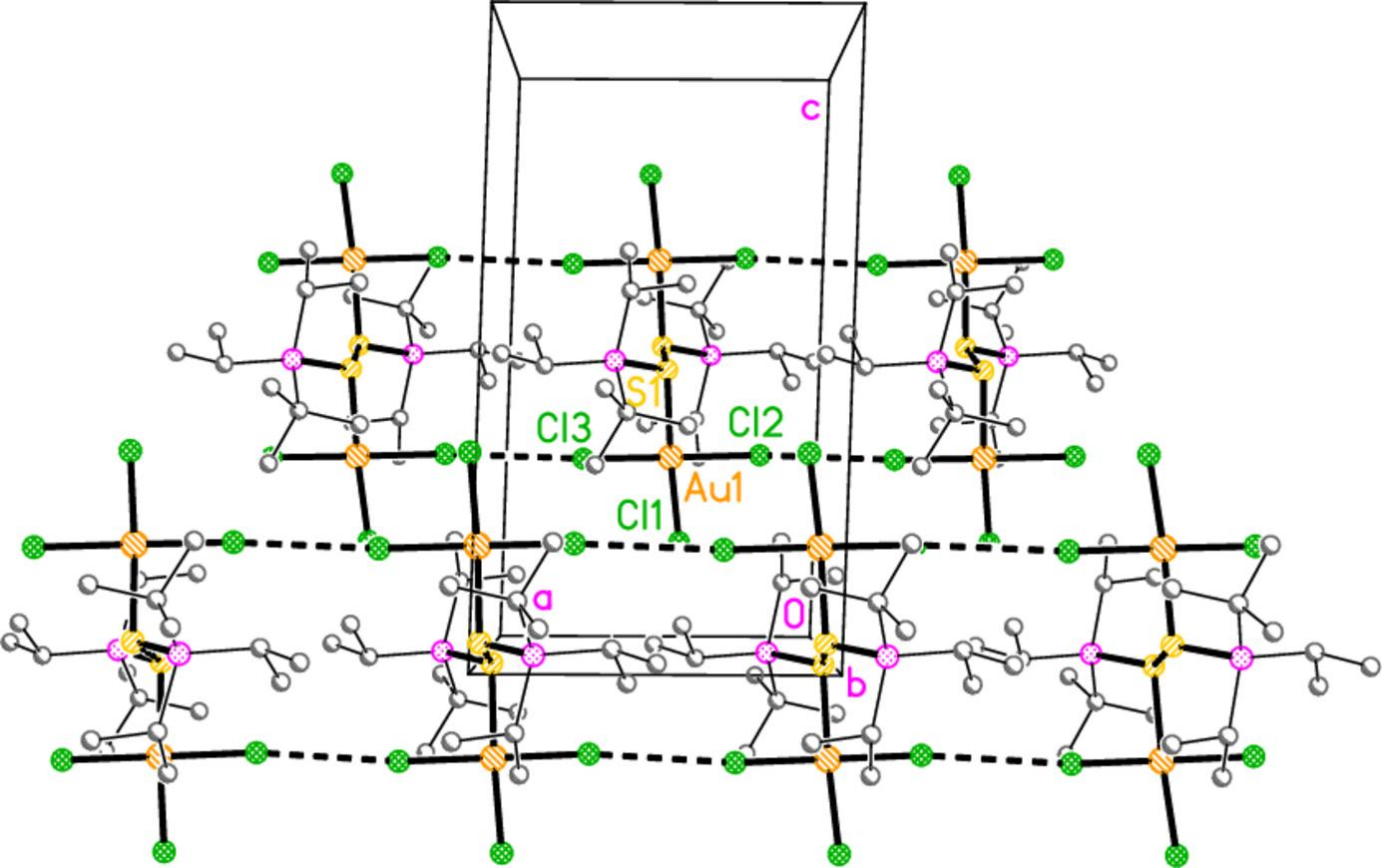
Structure **10aa**: Association of dimers to form chains parallel to the *a* axis. The two chains lie in the regions *y* ≃ 0.5, *z* ≃ 0 and *y* ≃ 0, *z* ≃ 0.5. Thick dashed lines indicate Cl⋯Cl and S⋯S contacts; the latter are viewed almost end-on. Hydrogen atoms are omitted.

**Figure 19:**
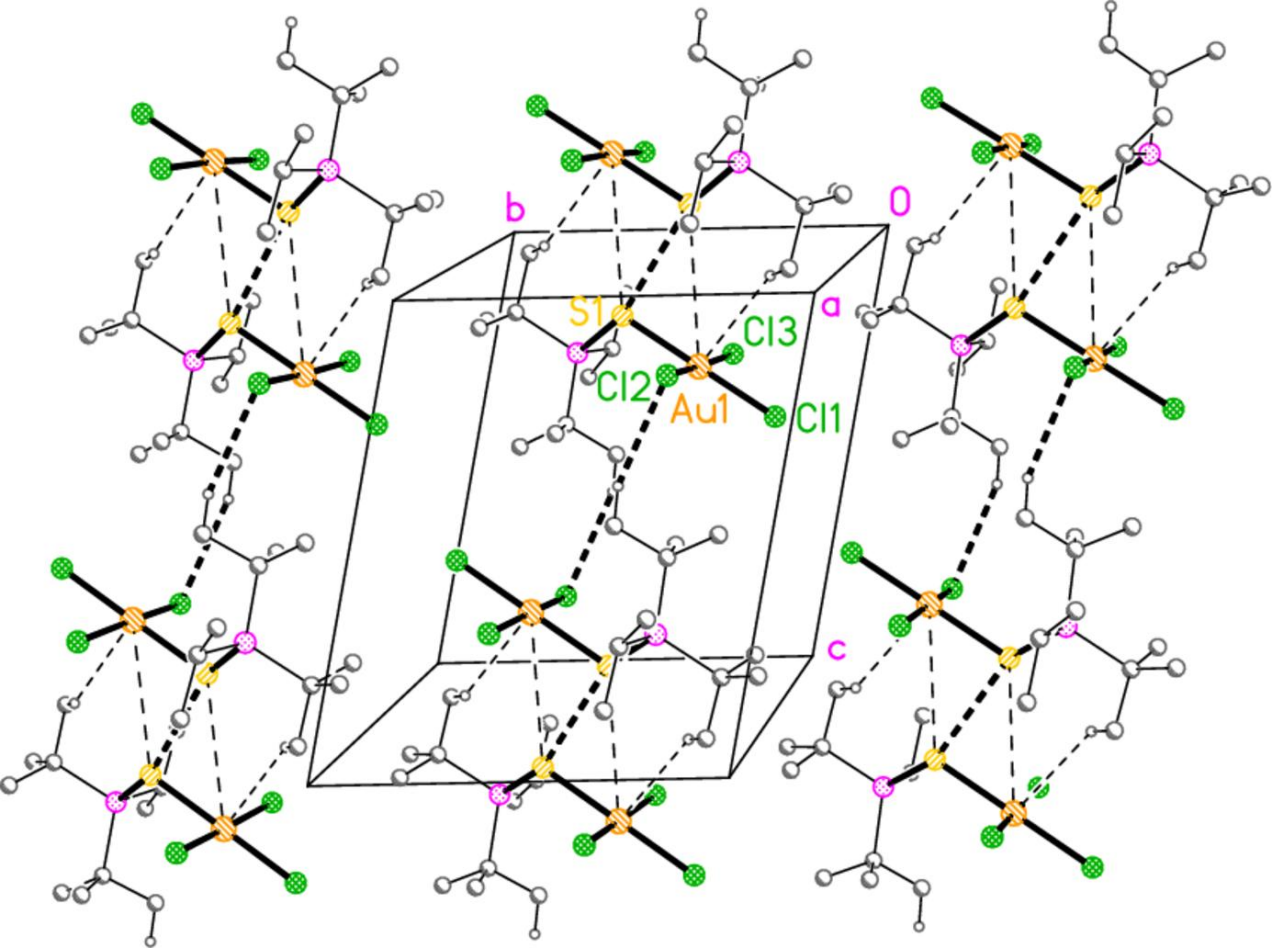
Compound **11a**: Association of dimers to form chains parallel to the *c* axis. The view direction is perpendicular to the *ab* plane. Dashed lines indicate H⋯Cl and S⋯S contacts (thick) or borderline S⋯Au and H⋯Au contacts (thin).

**Figure 20:**
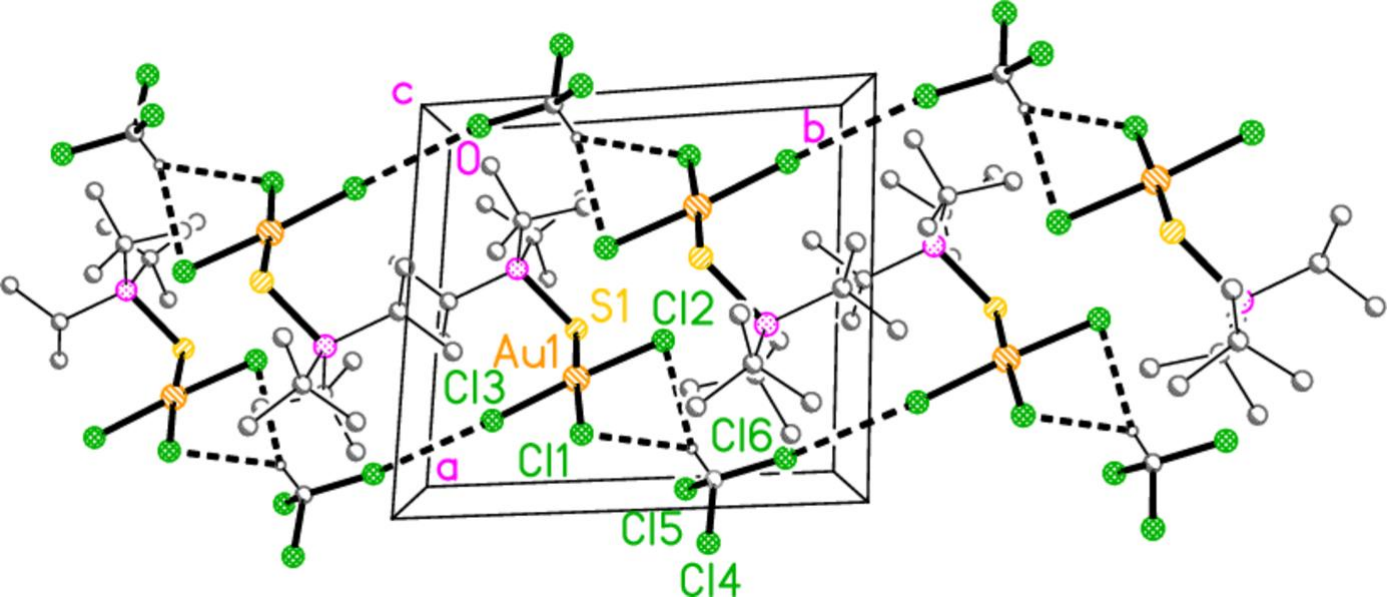
Compound **11aa**: Association of residues to form chains parallel to the *b* axis. The view direction is perpendicular to the *ab* plane. Dashed lines indicate hydrogen bonds or Cl⋯Cl contacts.

**Figure 21:**
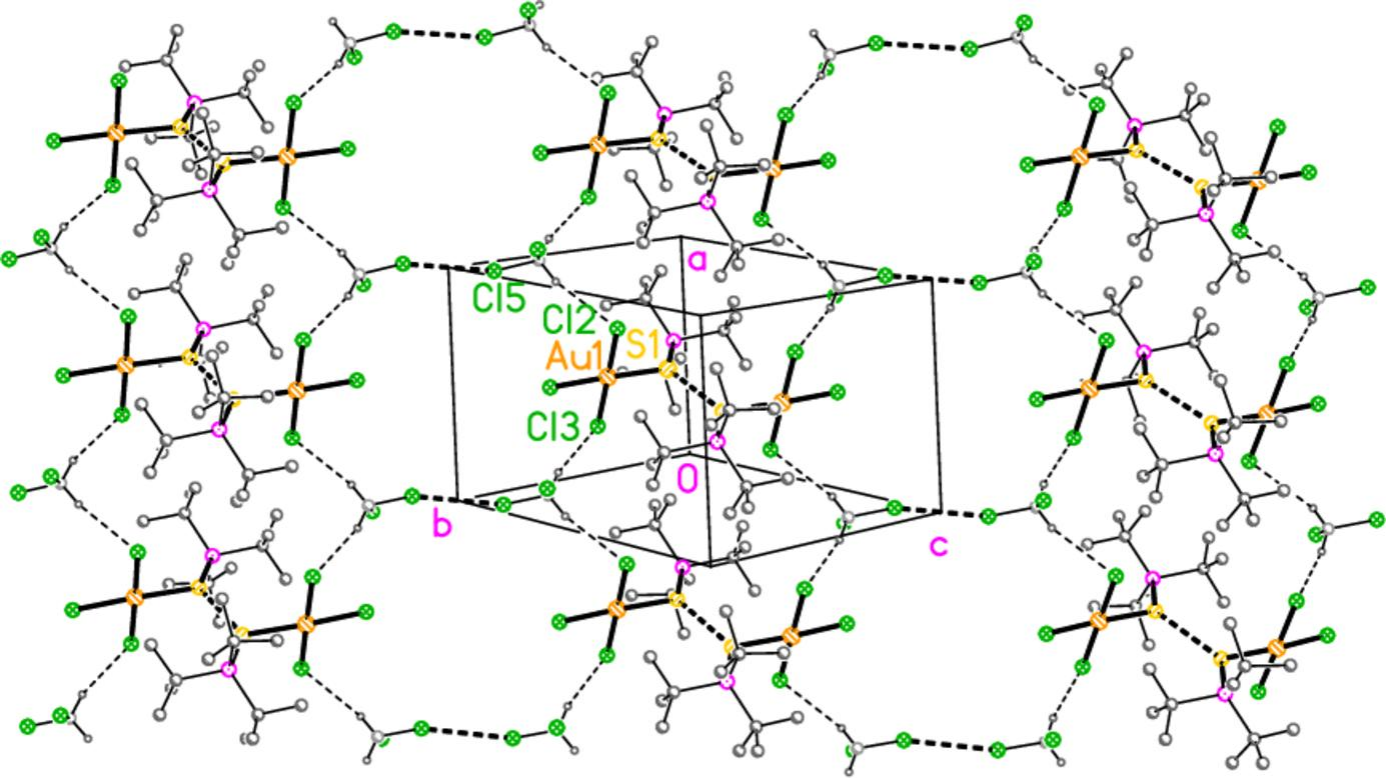
Compound **12a**: The layer structure parallel to (011), viewed perpendicular to this plane in the region *x* ≃ 1. Dashed lines indicate S⋯S and Cl⋯Cl contacts (thick) or H⋯Cl contacts (thin).

**Figure 22:**
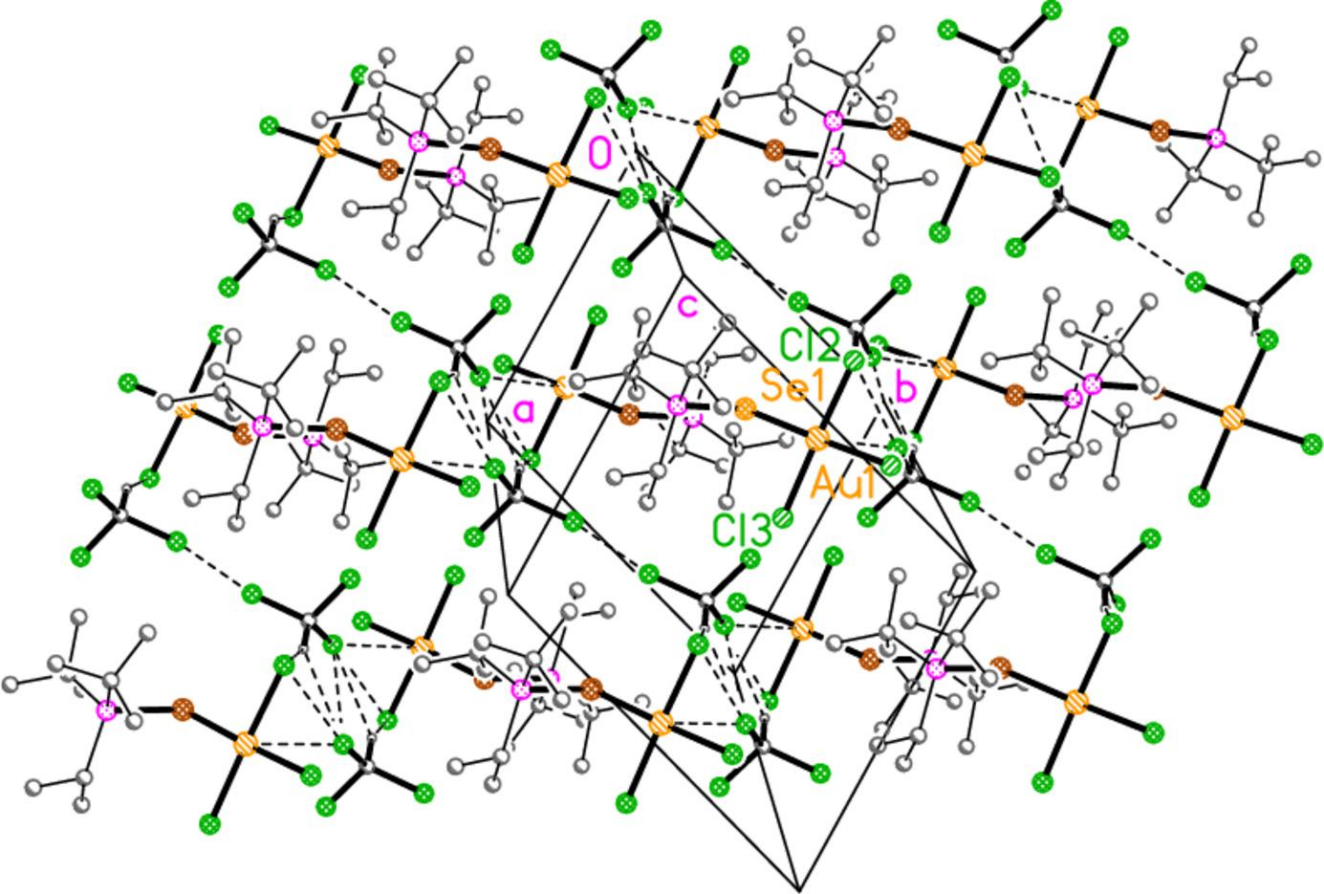
Compound **15aa**: The disordered solvent, only the major component of which is shown here, lies between the mol­ecules of the gold complex, forming a variety of short contacts Au⋯Cl, H⋯Cl and Cl⋯Cl (dashed lines). The view direction is perpendicular to the *ab* plane in the region *x* ≃ 0.

**Figure 23:**
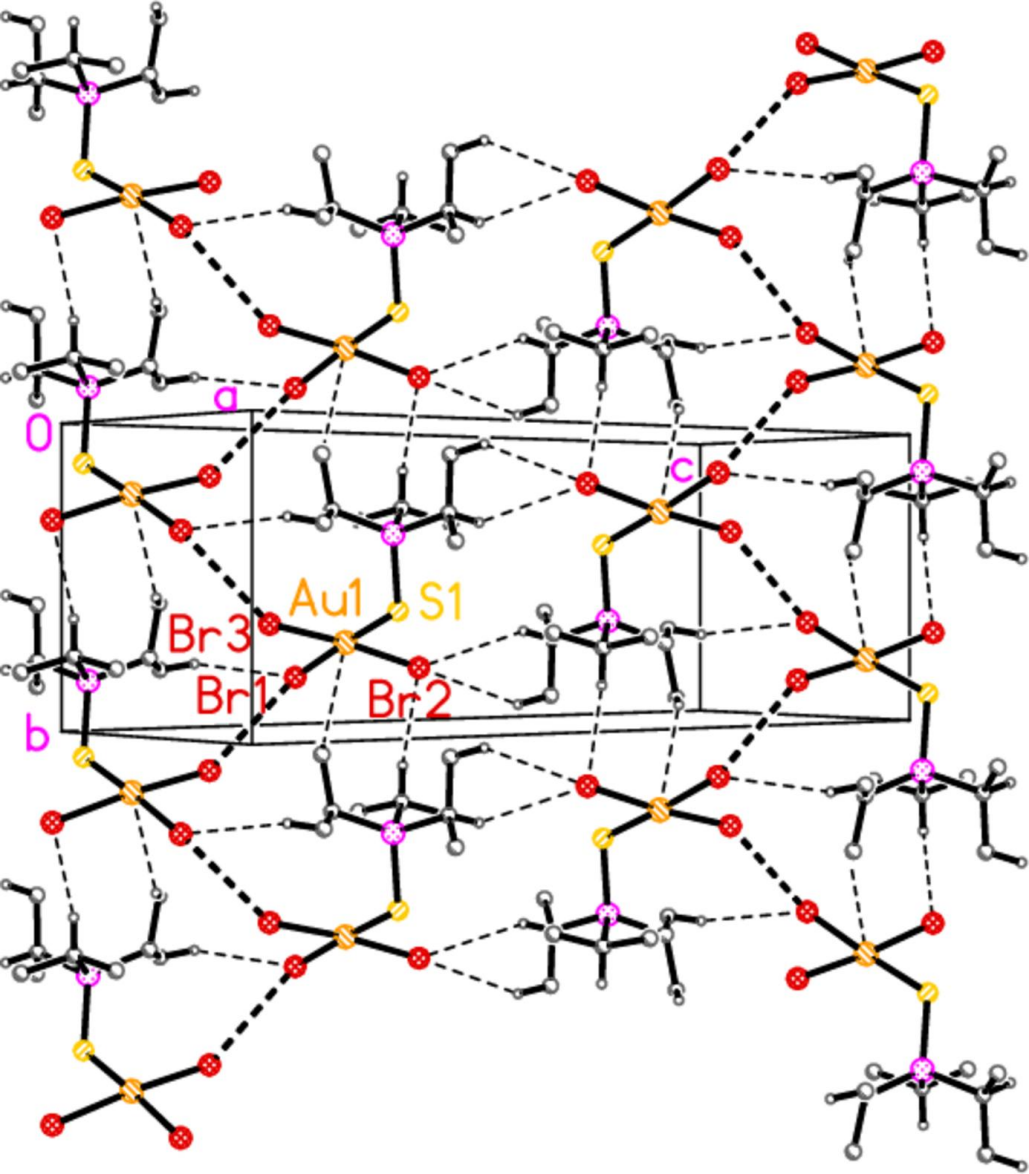
Compound **9b**: The layer structure parallel to (10 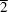 ), formed by Br⋯Br contacts (thick dashed lines) and five weak hydrogen bonds (thin dashed lines).

**Figure 24:**
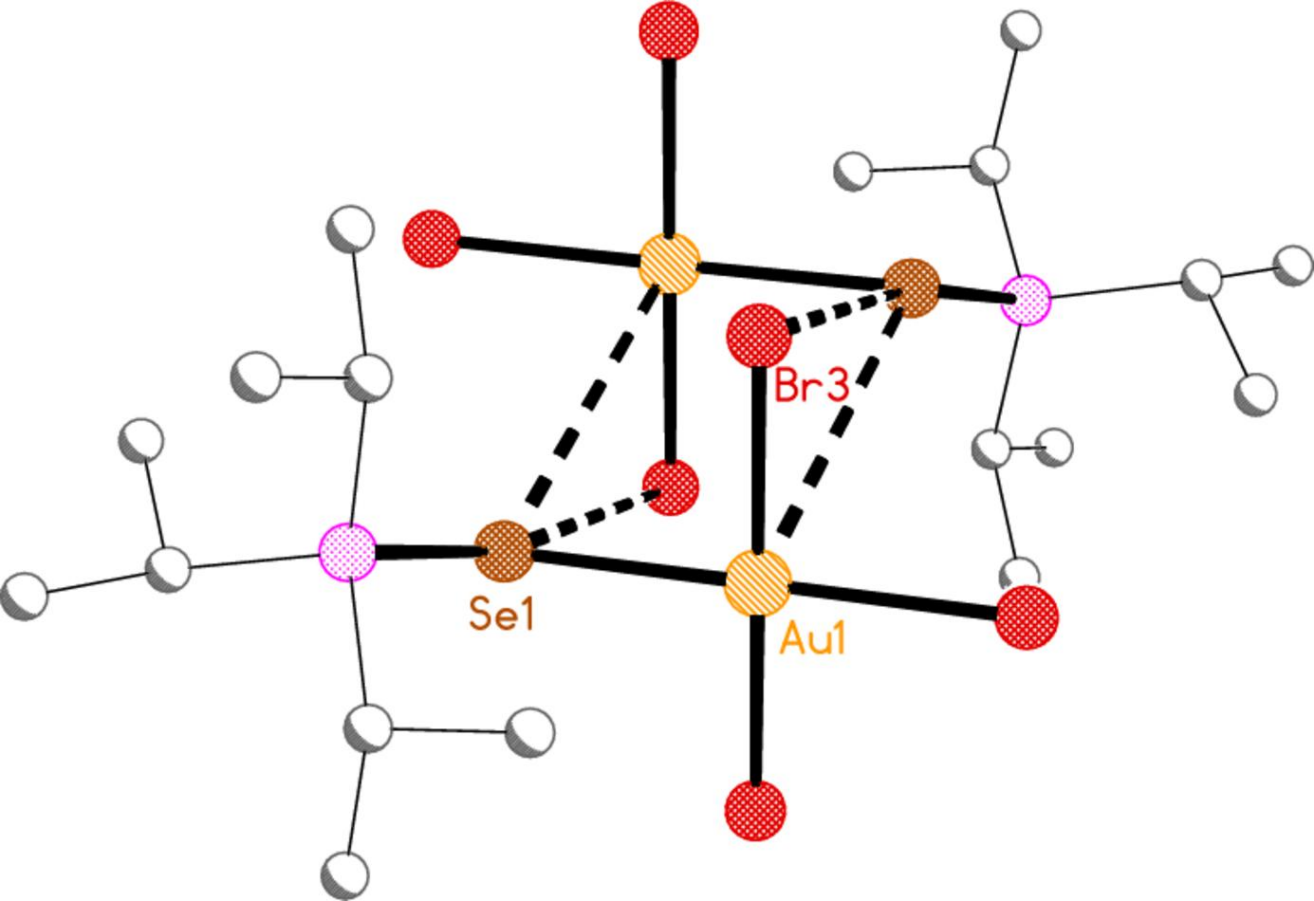
Compound **13b**: Dimer formation *via* short Se⋯Au and Se⋯Br contacts (thick dashed lines)

**Figure 25:**
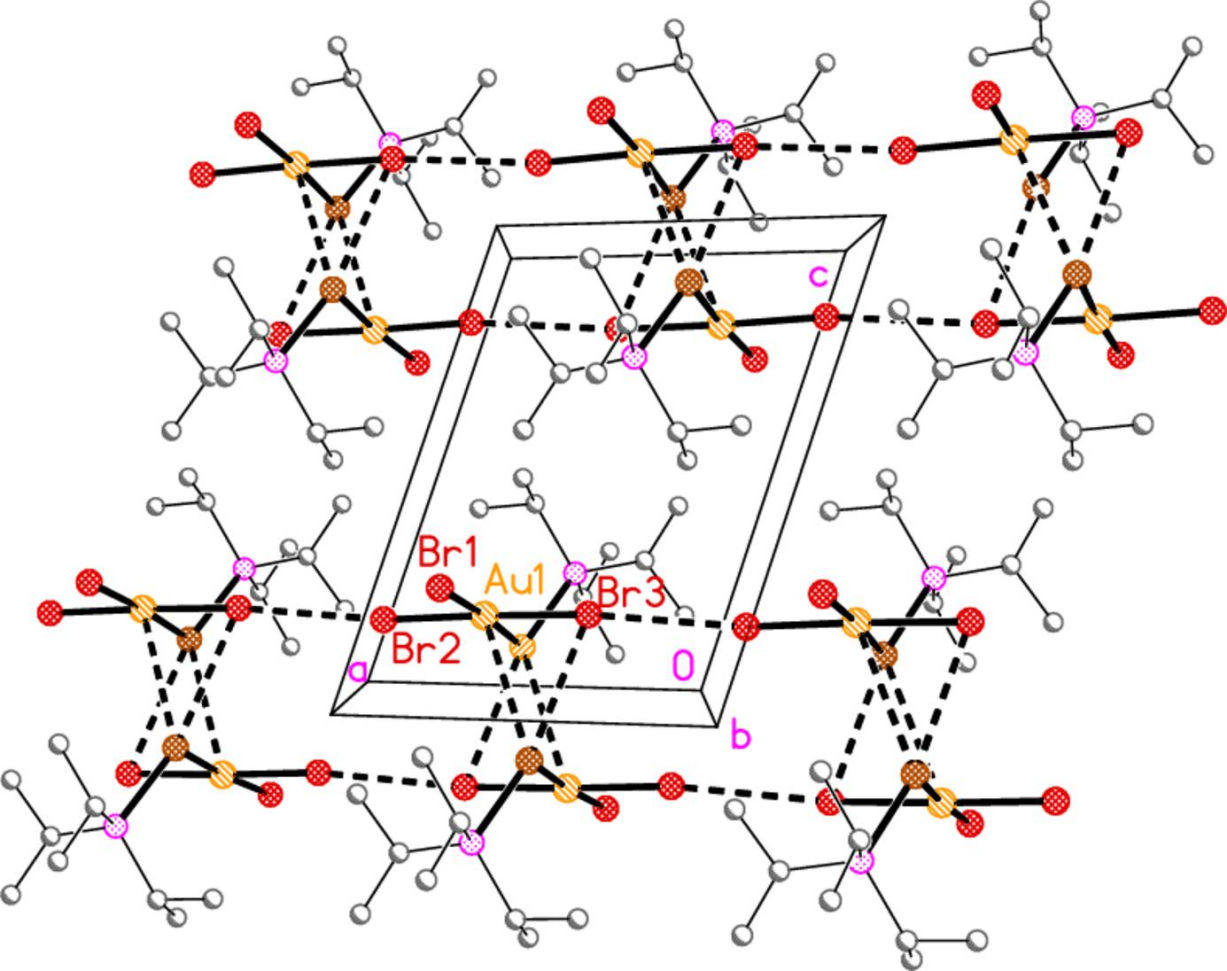
Compound **13b**: Linkage of dimers parallel to the *a* axis *via* a short Br⋯Br inter­action. The view direction is parallel to the *b* axis.

*Special features*: The deutero­chloro­form mol­ecule of **15aa** is disordered over two positions with occupation factors 0.525 (4) and 0.475 (4). Appropriate restraints were employed to improve refinement stability, but the dimensions of disordered groups should always be inter­preted with caution. The data for **14a** were significantly affected by the presence of a small (and at first undetected) satellite crystal, rotated by *ca* 5° from the main crystal. Attempts to treat the structure using procedures developed for non-merohedral twins did not lead to any improvement, and no better crystals were found. The *U* values are significantly higher than for the other structures, and the ellipsoid plot (Fig. 7 ▸) is drawn at 30% rather than 50% levels.

## Supplementary Material

Crystal structure: contains datablock(s) 9a, 10a, 10aa, 11a, 11aa, 12a, 14a, 15a, 15aa, 9b, 11b, 13b, 15b, global. DOI: 10.1107/S2056989024002032/yz2051sup1.cif

Structure factors: contains datablock(s) 9a. DOI: 10.1107/S2056989024002032/yz20519asup2.hkl

Structure factors: contains datablock(s) 10a. DOI: 10.1107/S2056989024002032/yz205110asup3.hkl

Structure factors: contains datablock(s) 10aa. DOI: 10.1107/S2056989024002032/yz205110aasup4.hkl

Structure factors: contains datablock(s) 11a. DOI: 10.1107/S2056989024002032/yz205111asup5.hkl

Structure factors: contains datablock(s) 11aa. DOI: 10.1107/S2056989024002032/yz205111aasup6.hkl

Structure factors: contains datablock(s) 12a. DOI: 10.1107/S2056989024002032/yz205112asup7.hkl

Structure factors: contains datablock(s) 14a. DOI: 10.1107/S2056989024002032/yz205114asup8.hkl

Structure factors: contains datablock(s) 15a. DOI: 10.1107/S2056989024002032/yz205115asup9.hkl

Structure factors: contains datablock(s) 15aa. DOI: 10.1107/S2056989024002032/yz205115aasup10.hkl

Structure factors: contains datablock(s) 9b. DOI: 10.1107/S2056989024002032/yz20519bsup11.hkl

Structure factors: contains datablock(s) 11b. DOI: 10.1107/S2056989024002032/yz205111bsup12.hkl

Structure factors: contains datablock(s) 13b. DOI: 10.1107/S2056989024002032/yz205113bsup13.hkl

Structure factors: contains datablock(s) 15b. DOI: 10.1107/S2056989024002032/yz205115bsup14.hkl

CCDC references: 2156390, 2156786, 2156787, 2156789, 2156790, 2156867, 2156868, 2156869, 2156870, 2338821, 2338822, 2338823, 2338824

Additional supporting information:  crystallographic information; 3D view; checkCIF report

## Figures and Tables

**Figure 2:**
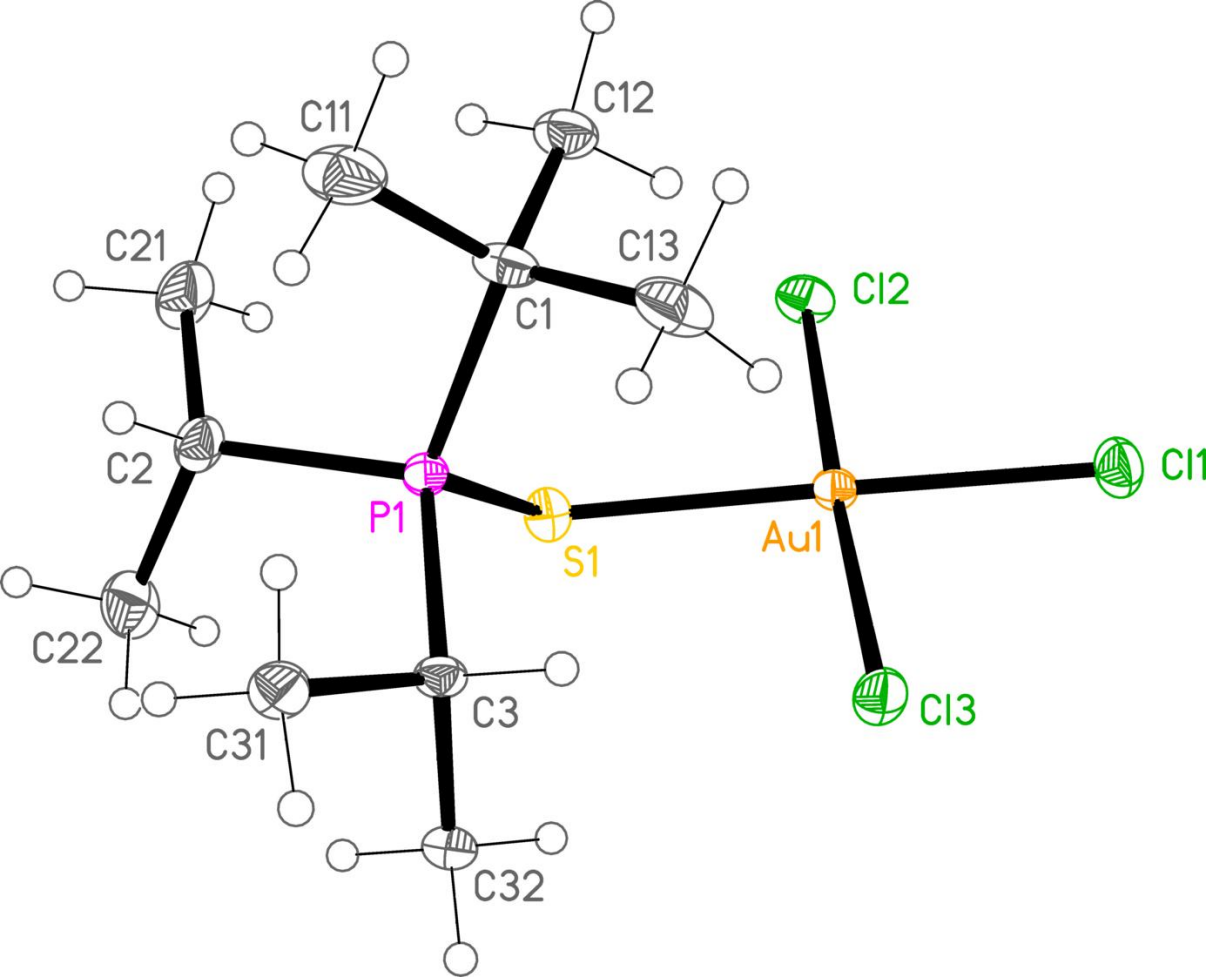
The structure of compound **10a** (the first polymorph) in the crystal. Ellipsoids represent 50% probability levels.

**Figure 3:**
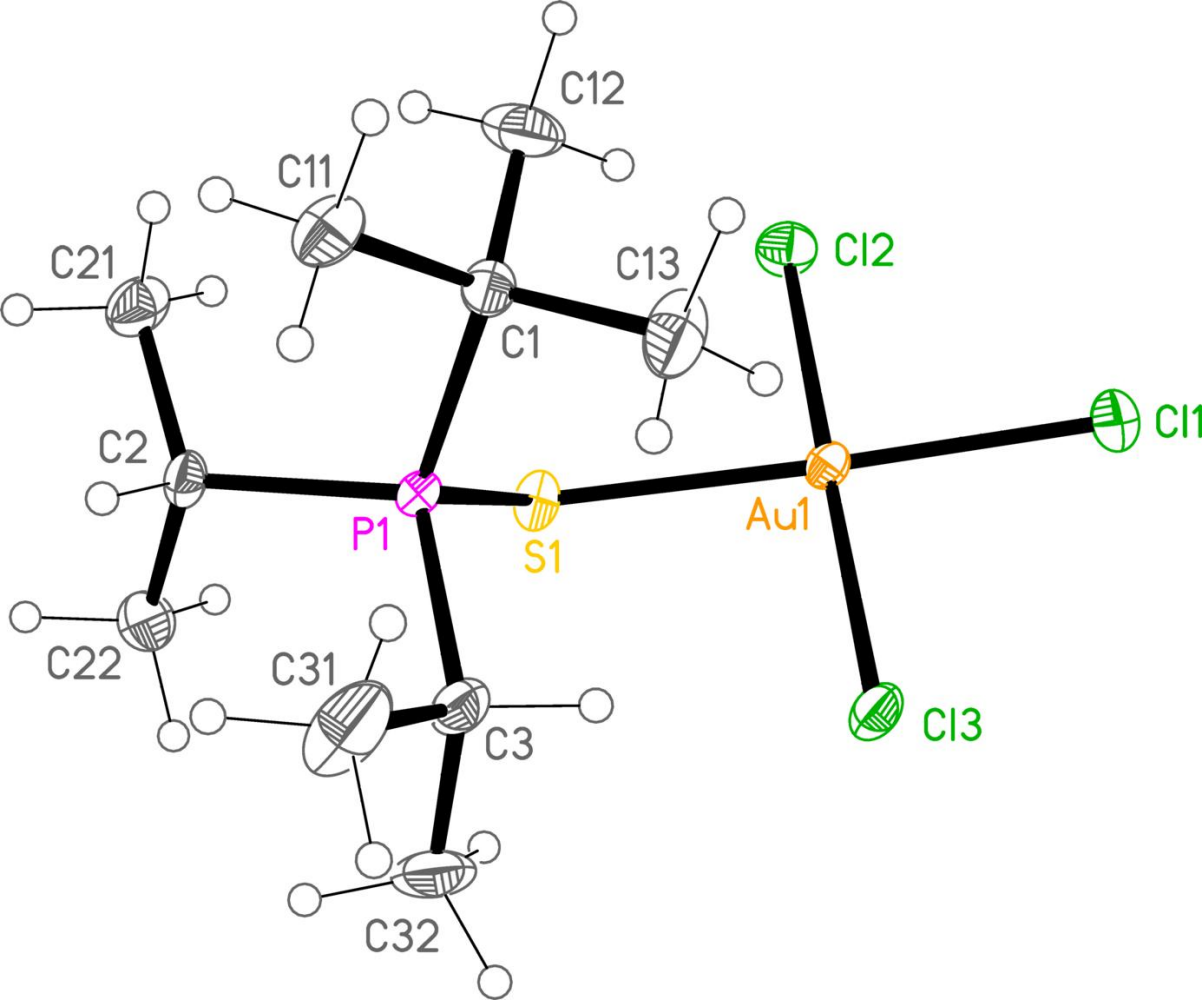
Structure **10aa** (the second polymorph of **10a**) in the crystal. Ellipsoids represent 50% probability levels.

**Figure 4:**
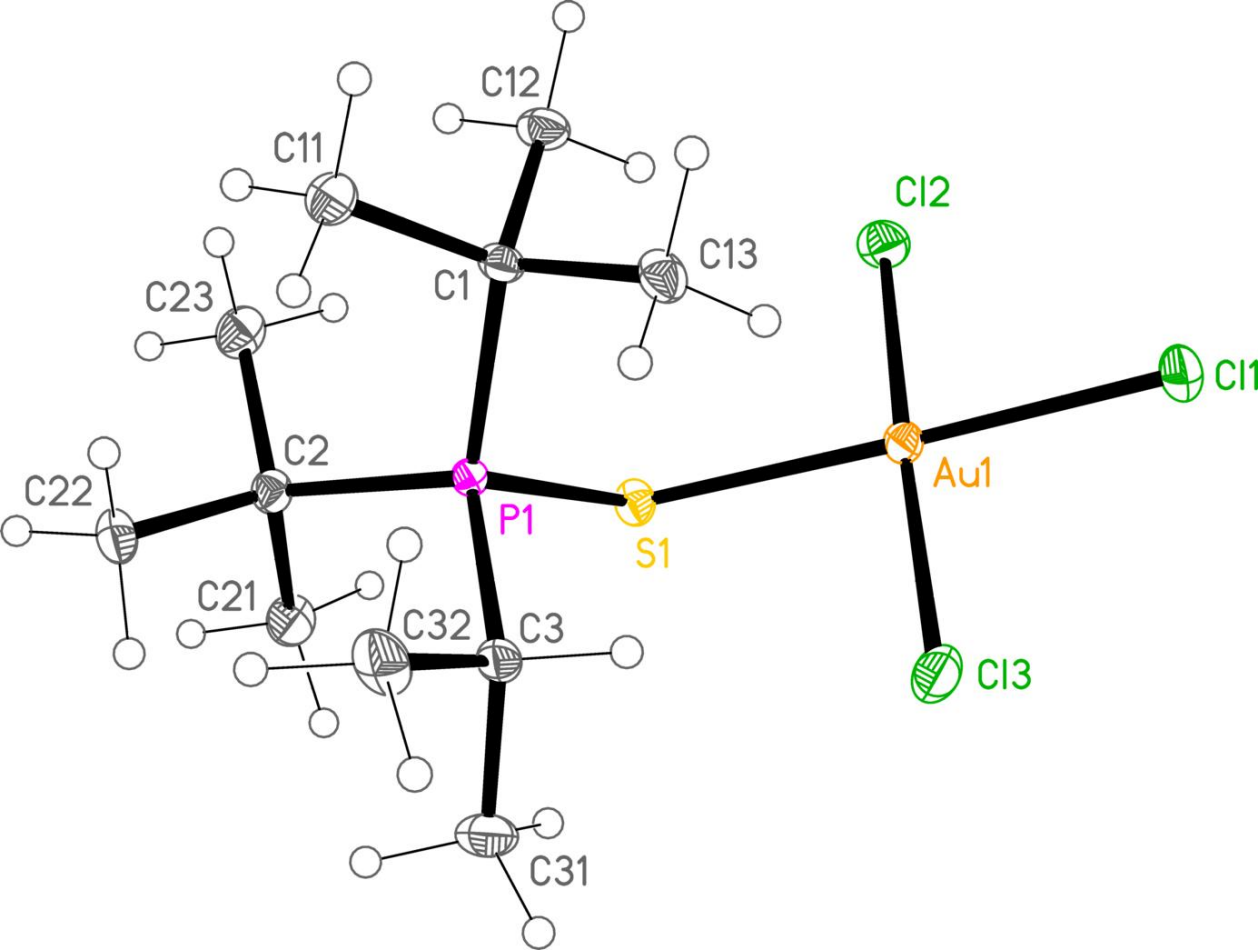
The structure of compound **11a** (the solvent-free form) in the crystal. Ellipsoids represent 50% probability levels.

**Figure 5:**
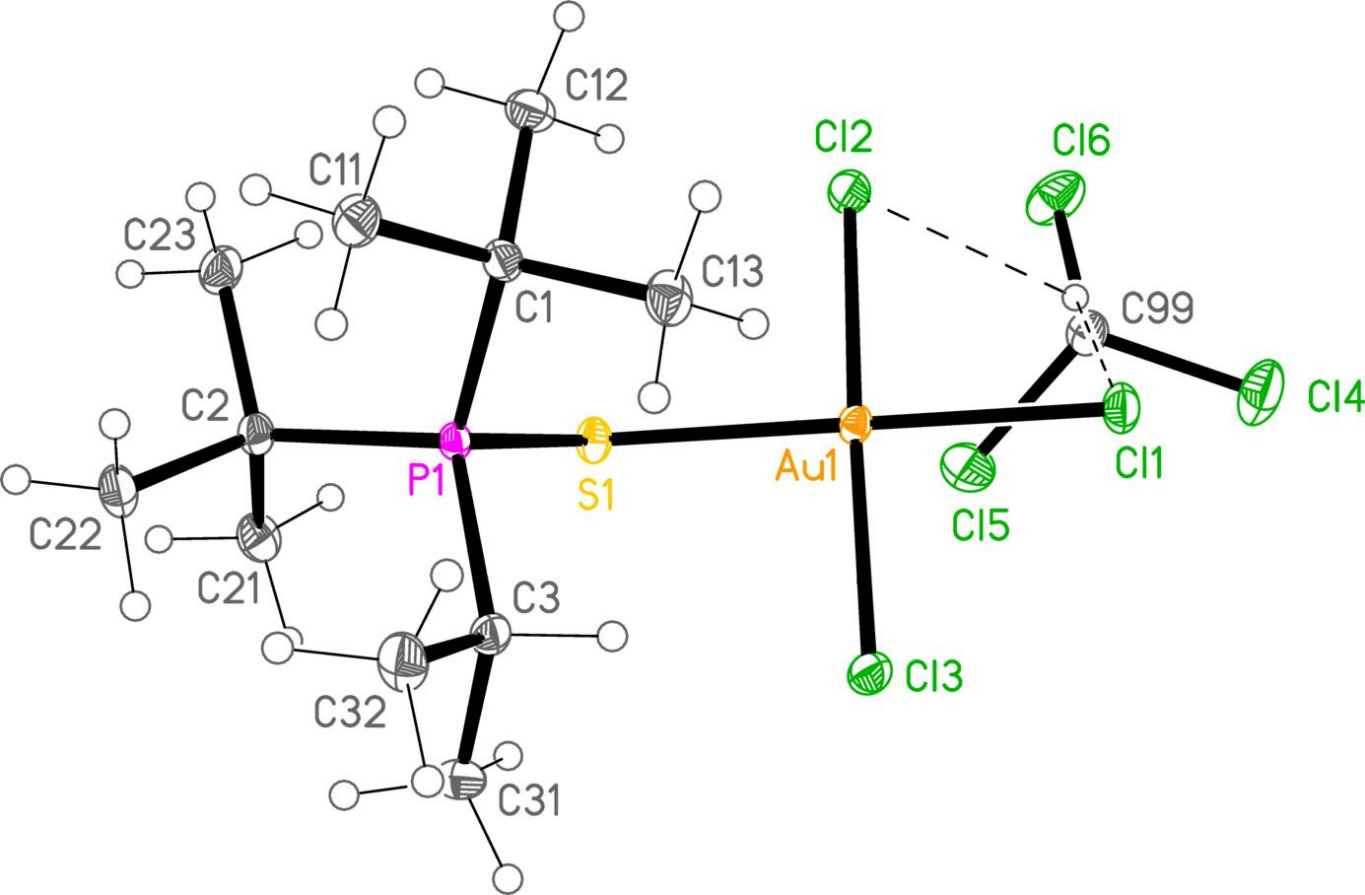
Structure **11aa** (the CDCl_3_ solvate of **11a**) in the crystal. Ellipsoids represent 50% probability levels. The dashed lines represent ‘weak’ hydrogen bonds.

**Figure 8:**
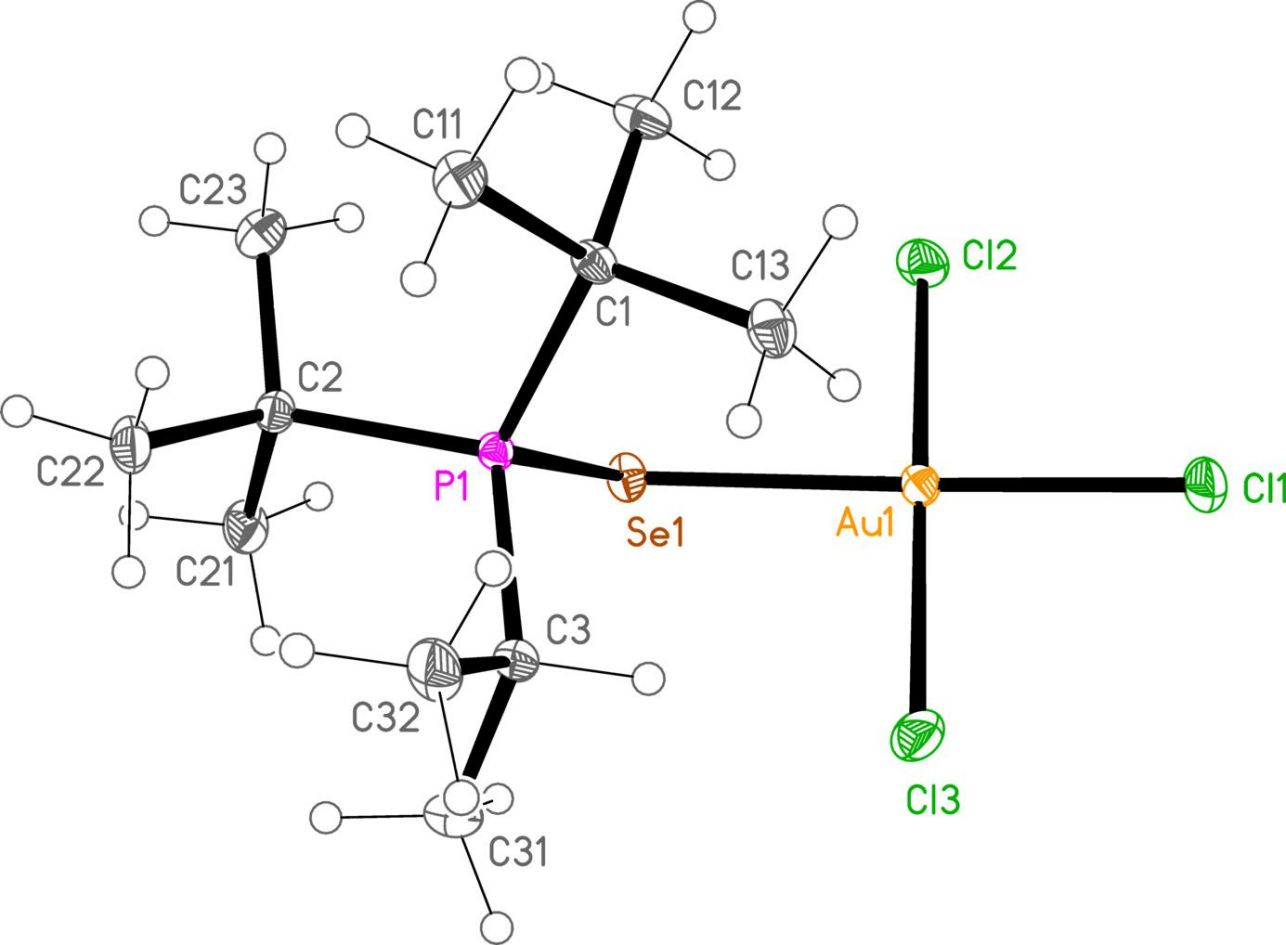
The structure of compound **15a** (the solvent-free form) in the crystal. Ellipsoids represent 50% probability levels.

**Figure 9:**
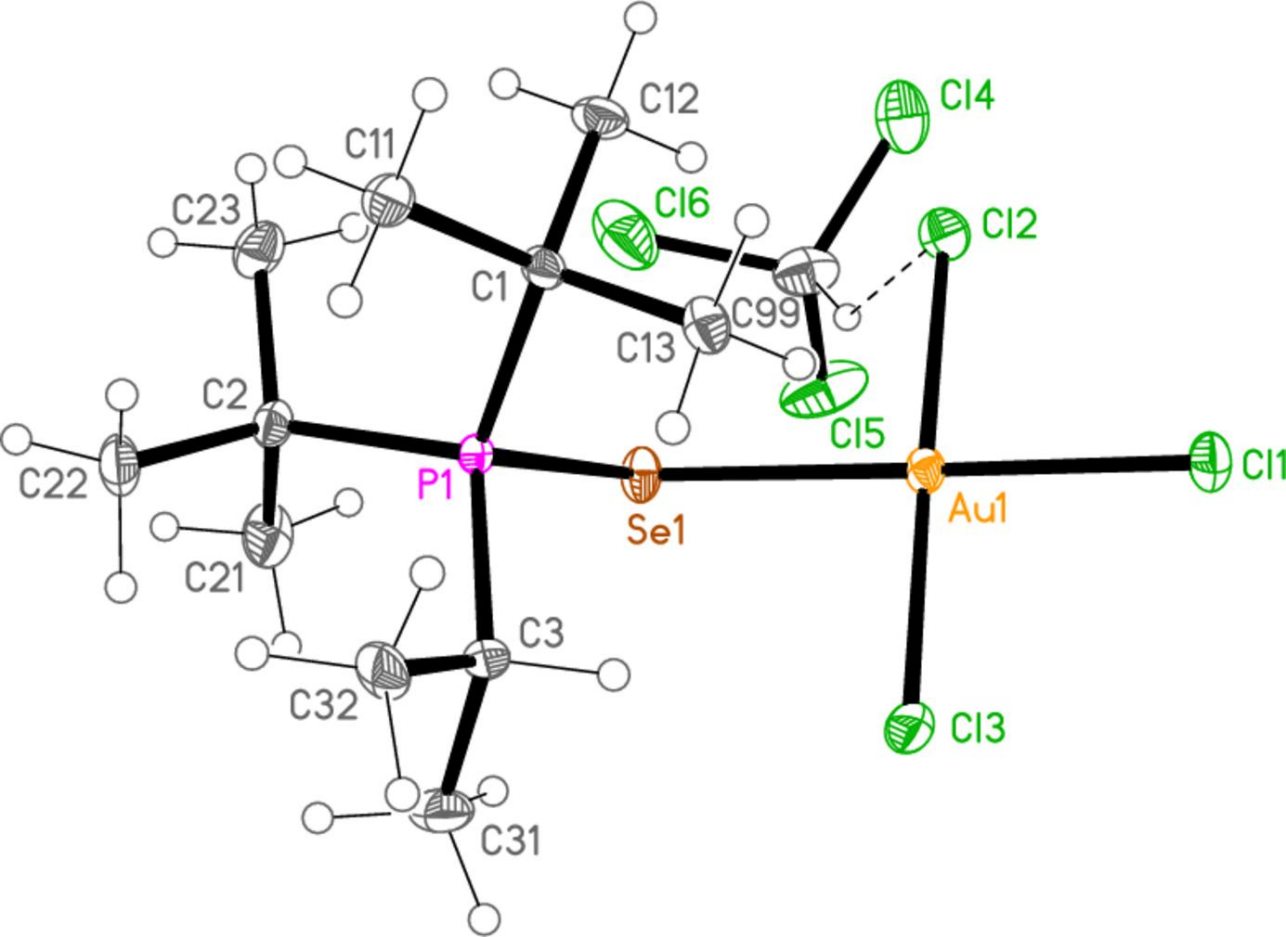
The structure of **15aa** (the CDCl_3_ solvate of **15a**) in the crystal. Ellipsoids represent 50% probability levels. Only one position of the disordered solvent is shown. The dashed line indicates a weak D⋯Cl hydrogen bond.

**Figure 10:**
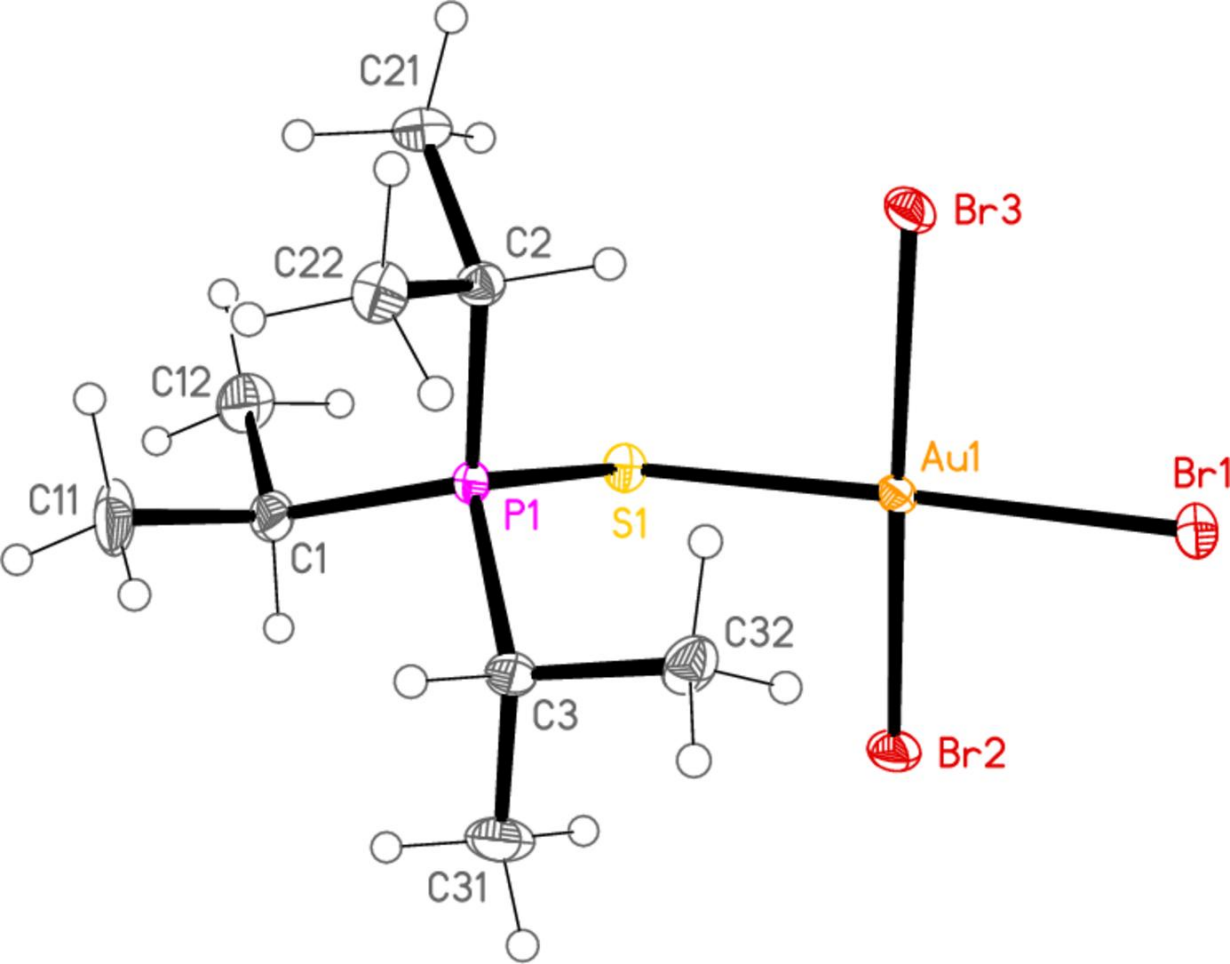
The structure of compound **9b** in the crystal. Ellipsoids represent 50% probability levels.

**Figure 11:**
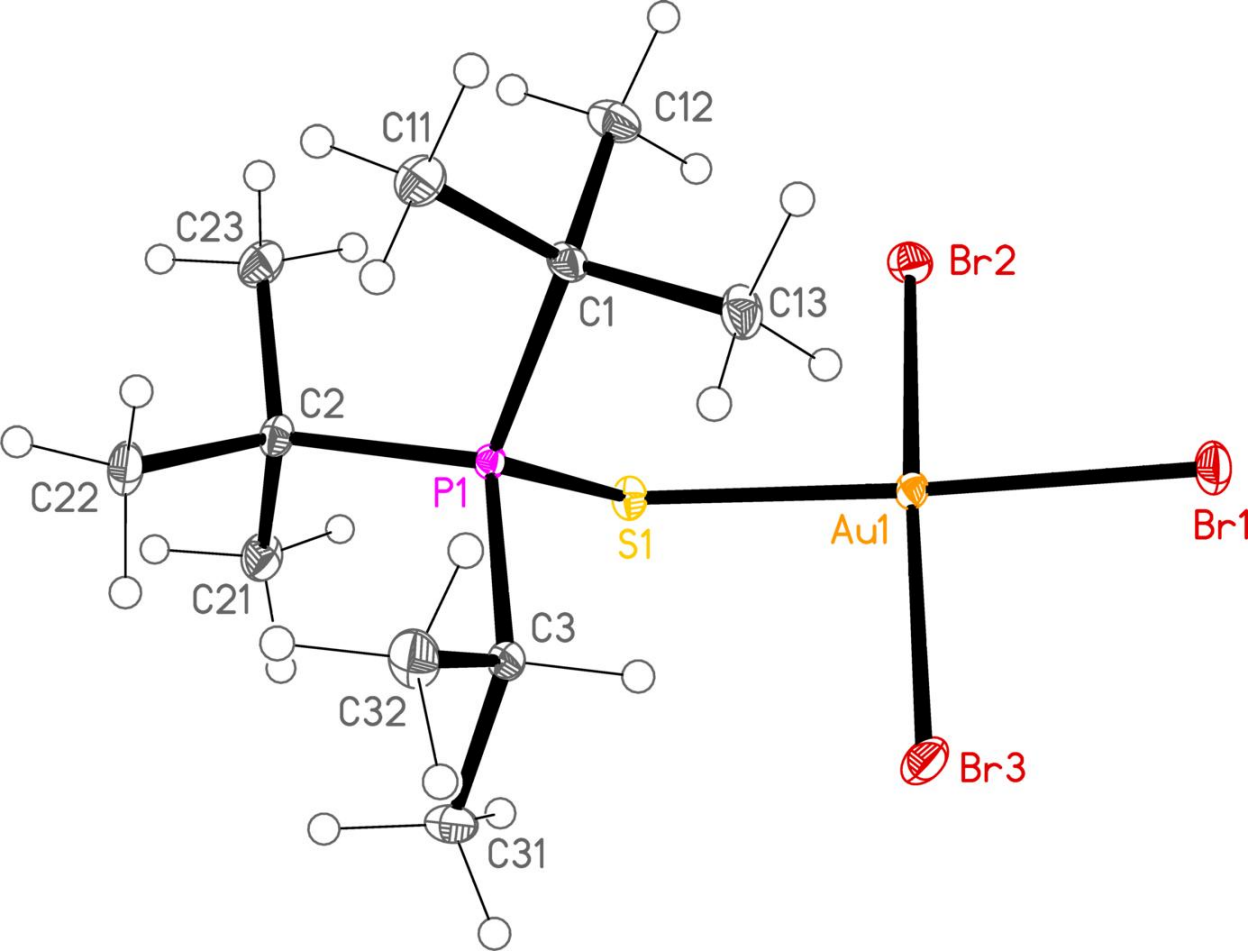
The structure of compound **11b** in the crystal. Ellipsoids represent 50% probability levels.

**Figure 12:**
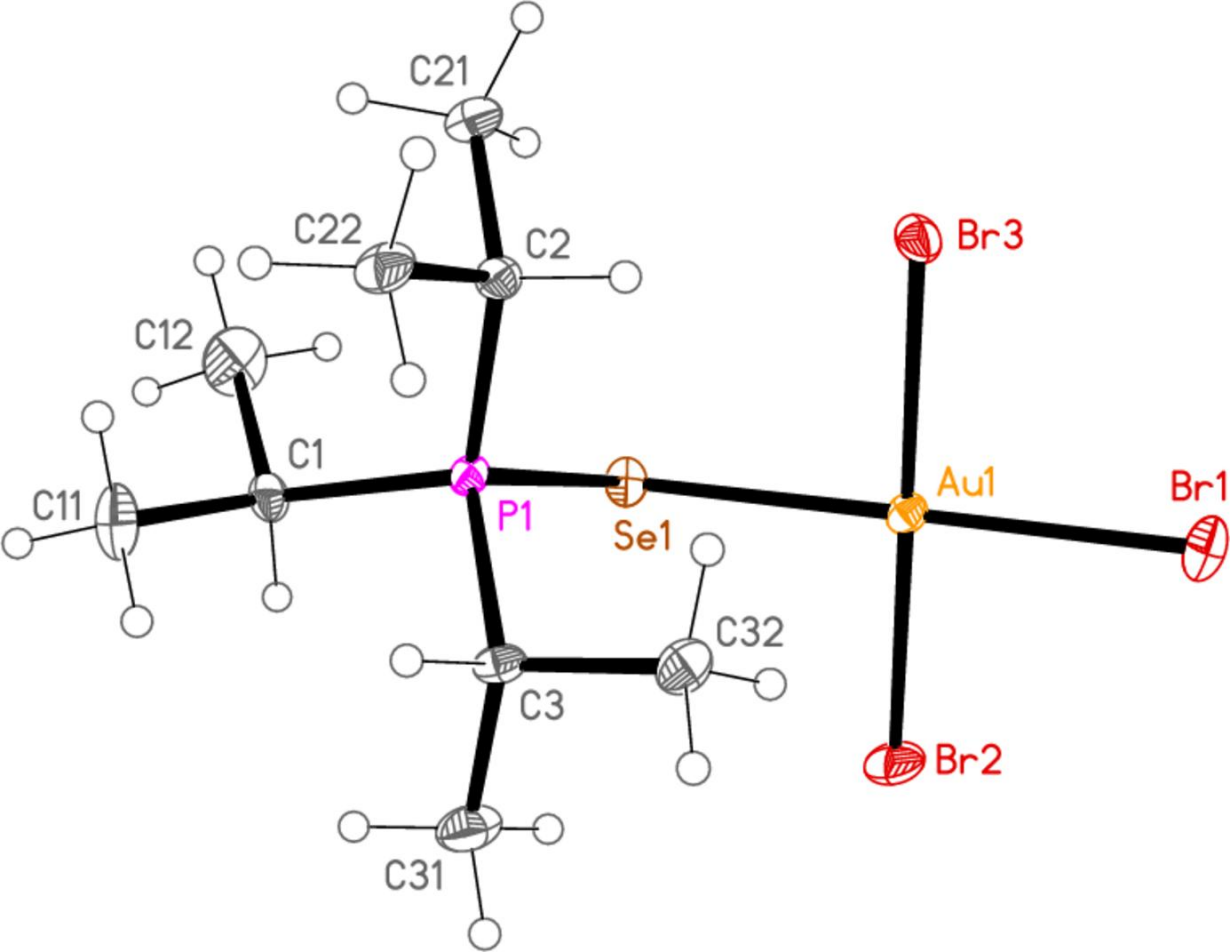
The structure of compound **13b** in the crystal. Ellipsoids represent 50% probability levels.

**Figure 13:**
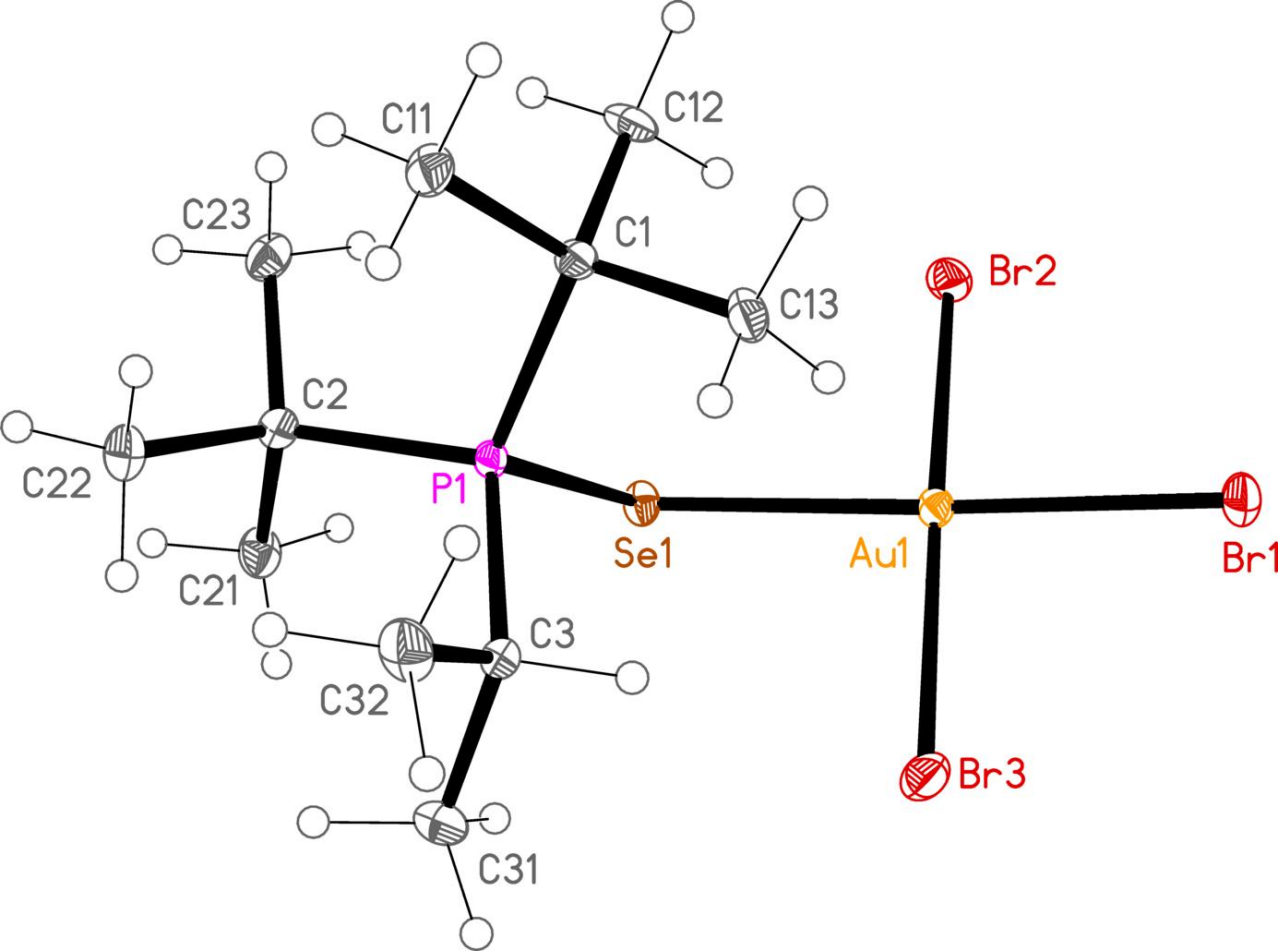
The structure of compound **15b** in the crystal. Ellipsoids represent 50% probability levels.

**Table 1:** Compositions of the *R* ^1^ *R* ^2^ *R* ^3^P*E*Au*X*_3_ structures presented in this paper (see Scheme)

Compound	*R* ^1^	*R* ^2^	*R* ^3^	*E*	*X*	Comments
**9a**	^*i*^Pr	^*i*^Pr	^*i*^Pr	S	Cl	
**10a**	^*i*^Pr	^*i*^Pr	^*t*^Bu	S	Cl	
**10aa**	^*i*^Pr	^*i*^Pr	^*t*^Bu	S	Cl	Second polymorph of **10*a***
**11a**	^*i*^Pr	^*t*^Bu	^*t*^Bu	S	Cl	
**11aa**	^*i*^Pr	^*t*^Bu	^*t*^Bu	S	Cl	CDCl_3_ solvate of **11*a***
**12a**	^*t*^Bu	^*t*^Bu	^*t*^Bu	S	Cl	CH_2_Cl_2_ solvate
**14a**	^*i*^Pr	^*i*^Pr	^*t*^Bu	Se	Cl	
**15a**	^*i*^Pr	^*t*^Bu	^*t*^Bu	Se	Cl	
**15aa**	^*i*^Pr	^*t*^Bu	^*t*^Bu	Se	Cl	CDCl_3_ solvate of **15*a***
**9b**	^*i*^Pr	^*i*^Pr	^*i*^Pr	S	Br	
**11b**	^*i*^Pr	^*t*^Bu	^*t*^Bu	S	Br	
**13b**	^*i*^Pr	^*i*^Pr	^*i*^Pr	Se	Br	
**15b**	^*i*^Pr	^*t*^Bu	^*t*^Bu	Se	Br	

**Table 2:** Selected geometric parameters (Å, °) for **9a** [Chem scheme1]

Au1—Cl3	2.2818 (5)	S1—P1	2.0574 (7)
Au1—Cl2	2.2846 (5)	P1—C2	1.829 (2)
Au1—Cl1	2.3064 (5)	P1—C3	1.8317 (19)
Au1—S1	2.3250 (5)	P1—C1	1.8387 (19)
			
Cl3—Au1—Cl2	175.341 (18)	C2—P1—C3	108.01 (9)
Cl3—Au1—Cl1	90.151 (18)	C2—P1—C1	115.39 (9)
Cl2—Au1—Cl1	88.999 (19)	C3—P1—C1	106.80 (9)
Cl3—Au1—S1	92.249 (18)	C2—P1—S1	111.92 (7)
Cl2—Au1—S1	88.545 (18)	C3—P1—S1	113.32 (7)
Cl1—Au1—S1	177.472 (17)	C1—P1—S1	101.34 (7)
P1—S1—Au1	106.70 (2)		
			
Cl3—Au1—S1—P1	−72.41 (3)	Au1—S1—P1—C3	−51.87 (8)
Cl2—Au1—S1—P1	112.19 (3)	Au1—S1—P1—C1	−165.92 (7)
Au1—S1—P1—C2	70.57 (7)		

**Table 3:** Selected geometric parameters (Å, °) for **10a** [Chem scheme1]

Au1—Cl3	2.2818 (6)	S1—P1	2.0538 (9)
Au1—Cl2	2.2837 (6)	P1—C2	1.843 (3)
Au1—Cl1	2.2989 (7)	P1—C3	1.845 (2)
Au1—S1	2.3294 (7)	P1—C1	1.875 (3)
			
Cl3—Au1—Cl2	177.46 (2)	C2—P1—C3	109.58 (12)
Cl3—Au1—Cl1	88.54 (2)	C2—P1—C1	109.67 (13)
Cl2—Au1—Cl1	89.23 (3)	C3—P1—C1	109.94 (12)
Cl3—Au1—S1	94.93 (2)	C2—P1—S1	102.70 (10)
Cl2—Au1—S1	87.39 (2)	C3—P1—S1	111.45 (9)
Cl1—Au1—S1	174.30 (2)	C1—P1—S1	113.25 (9)
P1—S1—Au1	111.29 (3)		
			
Cl3—Au1—S1—P1	56.17 (4)	Au1—S1—P1—C3	−77.45 (10)
Cl2—Au1—S1—P1	−122.80 (4)	Au1—S1—P1—C1	47.12 (11)
Au1—S1—P1—C2	165.31 (9)		

**Table 4:** Selected geometric parameters (Å, °) for **10aa** [Chem scheme1]

Au1—Cl3	2.2815 (8)	S1—P1	2.0592 (11)
Au1—Cl2	2.2851 (8)	P1—C3	1.836 (3)
Au1—Cl1	2.3116 (8)	P1—C2	1.839 (3)
Au1—S1	2.3281 (8)	P1—C1	1.871 (3)
			
Cl3—Au1—Cl2	176.43 (3)	C3—P1—C2	110.36 (15)
Cl3—Au1—Cl1	90.10 (3)	C3—P1—C1	108.88 (16)
Cl2—Au1—Cl1	89.18 (3)	C2—P1—C1	111.39 (14)
Cl3—Au1—S1	93.11 (3)	C3—P1—S1	111.66 (12)
Cl2—Au1—S1	87.51 (3)	C2—P1—S1	101.38 (11)
Cl1—Au1—S1	176.43 (3)	C1—P1—S1	113.03 (11)
P1—S1—Au1	111.18 (4)		
			
Cl3—Au1—S1—P1	61.27 (5)	Au1—S1—P1—C2	168.88 (11)
Cl2—Au1—S1—P1	−122.25 (5)	Au1—S1—P1—C1	49.55 (12)
Au1—S1—P1—C3	−73.62 (13)		

**Table 5:** Selected geometric parameters (Å, °) for **11a** [Chem scheme1]

Au1—Cl2	2.2881 (5)	S1—P1	2.0665 (6)
Au1—Cl3	2.2889 (5)	P1—C3	1.8442 (19)
Au1—Cl1	2.3080 (5)	P1—C2	1.8741 (18)
Au1—S1	2.3346 (5)	P1—C1	1.8765 (18)
			
Cl2—Au1—Cl3	175.769 (17)	C3—P1—C2	112.66 (9)
Cl2—Au1—Cl1	89.44 (2)	C3—P1—C1	108.88 (9)
Cl3—Au1—Cl1	89.35 (2)	C2—P1—C1	113.61 (8)
Cl2—Au1—S1	87.963 (19)	C3—P1—S1	109.17 (7)
Cl3—Au1—S1	93.175 (19)	C2—P1—S1	101.48 (6)
Cl1—Au1—S1	177.237 (19)	C1—P1—S1	110.81 (6)
P1—S1—Au1	111.35 (2)		
			
Cl2—Au1—S1—P1	−117.55 (3)	Au1—S1—P1—C2	169.16 (6)
Cl3—Au1—S1—P1	66.53 (3)	Au1—S1—P1—C1	48.21 (7)
Au1—S1—P1—C3	−71.70 (7)		

**Table 6:** Selected geometric parameters (Å, °) for **11aa** [Chem scheme1]

Au1—Cl3	2.2871 (5)	S1—P1	2.0622 (6)
Au1—Cl2	2.2903 (5)	P1—C3	1.8465 (19)
Au1—Cl1	2.3060 (4)	P1—C1	1.8737 (18)
Au1—S1	2.3312 (4)	P1—C2	1.8778 (18)
			
Cl3—Au1—Cl2	177.050 (16)	C3—P1—C1	108.94 (8)
Cl3—Au1—Cl1	88.505 (17)	C3—P1—C2	112.62 (8)
Cl2—Au1—Cl1	89.783 (17)	C1—P1—C2	114.39 (8)
Cl3—Au1—S1	92.936 (17)	C3—P1—S1	108.73 (6)
Cl2—Au1—S1	88.609 (16)	C1—P1—S1	110.75 (6)
Cl1—Au1—S1	175.816 (16)	C2—P1—S1	101.09 (6)
P1—S1—Au1	111.96 (2)		
			
Cl3—Au1—S1—P1	69.79 (3)	Au1—S1—P1—C1	52.74 (7)
Cl2—Au1—S1—P1	−112.73 (3)	Au1—S1—P1—C2	174.37 (6)
Au1—S1—P1—C3	−66.93 (7)		

**Table 7:** Selected geometric parameters (Å, °) for **12a** [Chem scheme1]

Au1—Cl3	2.2860 (5)	P1—C3	1.888 (2)
Au1—Cl2	2.2894 (6)	P1—C2	1.8906 (19)
Au1—Cl1	2.3013 (5)	P1—C1	1.906 (2)
Au1—S1	2.3323 (5)	P1—S1	2.0658 (6)
			
Cl3—Au1—Cl2	176.996 (18)	C3—P1—C1	111.29 (9)
Cl3—Au1—Cl1	88.53 (2)	C2—P1—C1	111.38 (9)
Cl2—Au1—Cl1	89.55 (2)	C3—P1—S1	110.17 (6)
Cl3—Au1—S1	93.402 (18)	C2—P1—S1	111.66 (6)
Cl2—Au1—S1	88.30 (2)	C1—P1—S1	99.33 (6)
Cl1—Au1—S1	174.441 (19)	P1—S1—Au1	117.50 (3)
C3—P1—C2	112.33 (9)		
			
C3—P1—S1—Au1	−78.75 (7)	Cl3—Au1—S1—P1	82.19 (3)
C2—P1—S1—Au1	46.80 (8)	Cl2—Au1—S1—P1	−100.28 (3)
C1—P1—S1—Au1	164.36 (6)		

**Table 8:** Selected geometric parameters (Å, °) for **14a** [Chem scheme1]

Au1—Cl3	2.2803 (19)	Se1—P1	2.211 (2)
Au1—Cl2	2.283 (2)	P1—C3	1.832 (8)
Au1—Cl1	2.326 (2)	P1—C2	1.845 (8)
Au1—Se1	2.4393 (8)	P1—C1	1.864 (8)
			
Cl3—Au1—Cl2	176.18 (8)	C3—P1—C2	110.8 (4)
Cl3—Au1—Cl1	90.53 (7)	C3—P1—C1	108.9 (4)
Cl2—Au1—Cl1	89.72 (8)	C2—P1—C1	111.3 (4)
Cl3—Au1—Se1	92.70 (5)	C3—P1—Se1	111.7 (3)
Cl2—Au1—Se1	86.98 (6)	C2—P1—Se1	101.0 (3)
Cl1—Au1—Se1	176.59 (6)	C1—P1—Se1	113.0 (3)
P1—Se1—Au1	108.25 (6)		
			
Cl3—Au1—Se1—P1	58.66 (8)	Au1—Se1—P1—C2	169.6 (3)
Cl2—Au1—Se1—P1	−125.16 (8)	Au1—Se1—P1—C1	50.7 (3)
Au1—Se1—P1—C3	−72.6 (3)		

**Table 9:** Selected geometric parameters (Å, °) for **15a** [Chem scheme1]

Au1—Cl2	2.2871 (5)	Se1—P1	2.2240 (5)
Au1—Cl3	2.2889 (5)	P1—C3	1.8435 (18)
Au1—Cl1	2.3207 (5)	P1—C2	1.8762 (18)
Au1—Se1	2.4460 (2)	P1—C1	1.8802 (17)
			
Cl2—Au1—Cl3	176.794 (17)	C3—P1—C2	112.81 (8)
Cl2—Au1—Cl1	89.966 (18)	C3—P1—C1	108.96 (8)
Cl3—Au1—Cl1	89.946 (18)	C2—P1—C1	114.08 (8)
Cl2—Au1—Se1	87.383 (14)	C3—P1—Se1	109.45 (6)
Cl3—Au1—Se1	92.617 (13)	C2—P1—Se1	101.19 (6)
Cl1—Au1—Se1	176.951 (14)	C1—P1—Se1	110.07 (5)
P1—Se1—Au1	108.487 (14)		
			
Cl2—Au1—Se1—P1	−118.493 (18)	Au1—Se1—P1—C2	170.76 (6)
Cl3—Au1—Se1—P1	64.717 (19)	Au1—Se1—P1—C1	49.76 (6)
Au1—Se1—P1—C3	−69.97 (6)		

**Table 10:** Selected geometric parameters (Å, °) for **15aa** [Chem scheme1]

Au1—Cl3	2.2825 (7)	Se1—P1	2.2232 (6)
Au1—Cl2	2.2889 (6)	P1—C3	1.844 (3)
Au1—Cl1	2.3172 (6)	P1—C2	1.874 (2)
Au1—Se1	2.4476 (3)	P1—C1	1.878 (2)
			
Cl3—Au1—Cl2	177.64 (2)	C3—P1—C2	112.97 (12)
Cl3—Au1—Cl1	89.67 (2)	C3—P1—C1	108.44 (11)
Cl2—Au1—Cl1	89.66 (2)	C2—P1—C1	114.23 (11)
Cl3—Au1—Se1	92.332 (18)	C3—P1—Se1	108.64 (9)
Cl2—Au1—Se1	88.238 (18)	C2—P1—Se1	101.78 (8)
Cl1—Au1—Se1	176.855 (18)	C1—P1—Se1	110.54 (8)
P1—Se1—Au1	107.617 (18)		
			
Cl3—Au1—Se1—P1	68.16 (3)	Au1—Se1—P1—C2	170.69 (8)
Cl2—Au1—Se1—P1	−114.14 (3)	Au1—Se1—P1—C1	48.97 (9)
Au1—Se1—P1—C3	−69.90 (9)		

**Table 11:** Selected geometric parameters (Å, °) for **9b** [Chem scheme1]

Au1—S1	2.3413 (7)	P1—C2	1.828 (3)
Au1—Br3	2.4233 (3)	P1—C3	1.830 (3)
Au1—Br2	2.4333 (3)	P1—C1	1.832 (3)
Au1—Br1	2.4341 (3)	P1—S1	2.0523 (10)
			
S1—Au1—Br3	92.317 (19)	C2—P1—C1	114.26 (14)
S1—Au1—Br2	88.43 (2)	C3—P1—C1	106.86 (14)
Br3—Au1—Br2	175.188 (12)	C2—P1—S1	111.90 (10)
S1—Au1—Br1	177.35 (2)	C3—P1—S1	113.79 (11)
Br3—Au1—Br1	89.771 (11)	C1—P1—S1	102.26 (11)
Br2—Au1—Br1	89.350 (11)	P1—S1—Au1	107.77 (4)
C2—P1—C3	107.76 (14)		
			
C2—P1—S1—Au1	72.07 (10)	Br3—Au1—S1—P1	−74.36 (4)
C3—P1—S1—Au1	−50.38 (12)	Br2—Au1—S1—P1	110.40 (4)
C1—P1—S1—Au1	−165.21 (10)		

**Table 12:** Selected geometric parameters (Å, °) for **11b** [Chem scheme1]

Au1—S1	2.3477 (6)	P1—C3	1.847 (2)
Au1—Br3	2.4310 (3)	P1—C2	1.872 (2)
Au1—Br2	2.4330 (3)	P1—C1	1.877 (2)
Au1—Br1	2.4399 (4)	P1—S1	2.0640 (8)
			
S1—Au1—Br3	93.532 (19)	C3—P1—C1	108.53 (11)
S1—Au1—Br2	87.959 (19)	C2—P1—C1	113.56 (10)
Br3—Au1—Br2	172.720 (9)	C3—P1—S1	109.09 (8)
S1—Au1—Br1	177.293 (16)	C2—P1—S1	101.70 (7)
Br3—Au1—Br1	89.099 (14)	C1—P1—S1	111.23 (7)
Br2—Au1—Br1	89.510 (14)	P1—S1—Au1	111.56 (3)
C3—P1—C2	112.57 (10)		
			
C3—P1—S1—Au1	−70.82 (8)	Br3—Au1—S1—P1	70.22 (3)
C2—P1—S1—Au1	170.07 (8)	Br2—Au1—S1—P1	−116.91 (3)
C1—P1—S1—Au1	48.86 (9)		

**Table 13:** Selected geometric parameters (Å, °) for **13b** [Chem scheme1]

Au1—Br2	2.4241 (4)	P1—C2	1.831 (4)
Au1—Br3	2.4321 (4)	P1—C1	1.835 (4)
Au1—Se1	2.4535 (4)	P1—C3	1.835 (4)
Au1—Br1	2.4597 (5)	P1—Se1	2.2085 (11)
			
Br2—Au1—Br3	178.373 (15)	C2—P1—C3	107.15 (18)
Br2—Au1—Se1	87.616 (16)	C1—P1—C3	107.53 (19)
Br3—Au1—Se1	91.709 (14)	C2—P1—Se1	112.81 (14)
Br2—Au1—Br1	90.566 (17)	C1—P1—Se1	101.08 (14)
Br3—Au1—Br1	90.031 (16)	C3—P1—Se1	112.75 (13)
Se1—Au1—Br1	176.522 (15)	P1—Se1—Au1	107.24 (3)
C2—P1—C1	115.50 (19)		
			
C2—P1—Se1—Au1	67.97 (14)	Br2—Au1—Se1—P1	114.96 (3)
C1—P1—Se1—Au1	−168.10 (13)	Br3—Au1—Se1—P1	−66.53 (3)
C3—P1—Se1—Au1	−53.58 (15)		

**Table 14:** Selected geometric parameters (Å, °) for **15b** [Chem scheme1]

Au1—Br2	2.4302 (3)	P1—C3	1.847 (2)
Au1—Br3	2.4320 (3)	P1—C2	1.875 (3)
Au1—Br1	2.4549 (3)	P1—C1	1.883 (3)
Au1—Se1	2.4606 (3)	P1—Se1	2.2247 (7)
			
Br2—Au1—Br3	174.078 (10)	C3—P1—C1	108.84 (11)
Br2—Au1—Br1	90.066 (11)	C2—P1—C1	114.02 (11)
Br3—Au1—Br1	89.729 (12)	C3—P1—Se1	109.35 (9)
Br2—Au1—Se1	87.317 (11)	C2—P1—Se1	101.46 (8)
Br3—Au1—Se1	92.892 (11)	C1—P1—Se1	110.34 (8)
Br1—Au1—Se1	177.377 (9)	P1—Se1—Au1	108.81 (2)
C3—P1—C2	112.60 (11)		
			
C3—P1—Se1—Au1	−69.41 (9)	Br2—Au1—Se1—P1	−117.58 (2)
C2—P1—Se1—Au1	171.47 (8)	Br3—Au1—Se1—P1	68.34 (2)
C1—P1—Se1—Au1	50.27 (9)		

**Table 15:** Hydrogen-bond geometry (Å, °) for **9a** [Chem scheme1]

*D*—H⋯*A*	*D*—H	H⋯*A*	*D*⋯*A*	*D*—H⋯*A*
C32—H32*C*⋯Au1	0.98	2.69	3.485 (2)	138
C2—H2⋯Cl3	1.00	2.68	3.437 (2)	133
C12—H12*B*⋯S1	0.98	2.73	3.310 (2)	118
C1—H1⋯Cl2^i^	1.00	2.82	3.506 (2)	126
C11—H11*C*⋯Cl2^i^	0.98	2.91	3.582 (2)	127
C22—H22*A*⋯Cl1^ii^	0.98	2.86	3.803 (2)	161
C2—H2⋯Cl3^ii^	1.00	2.86	3.688 (2)	141
C32—H32*A*⋯Cl2^iii^	0.98	2.87	3.847 (2)	180
C3—H3⋯Cl2^iv^	1.00	2.95	3.881 (2)	155

Symmetry codes: (i) 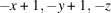 ; (ii) 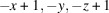 ; (iii) 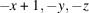 ; (iv) 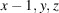 .

**Table 16:** Hydrogen-bond geometry (Å, °) for **10a** [Chem scheme1]

*D*—H⋯*A*	*D*—H	H⋯*A*	*D*⋯*A*	*D*—H⋯*A*
C32—H32*B*⋯Cl3	0.98	2.75	3.452 (3)	130
C3—H3⋯Cl3	1.00	2.89	3.476 (3)	118
C13—H13*A*⋯Cl3	0.98	2.85	3.729 (3)	150
C13—H13*B*⋯Cl2^i^	0.98	2.77	3.704 (3)	160
C31—H31*A*⋯Cl1^ii^	0.98	2.87	3.372 (3)	113
C13—H13*C*⋯Cl2^iii^	0.98	2.91	3.878 (3)	170
C32—H32*A*⋯Cl3^iv^	0.98	2.91	3.800 (3)	152

Symmetry codes: (i) 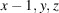 ; (ii) 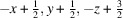 ; (iii) 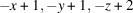 ; (iv) 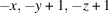 .

**Table 17:** Hydrogen-bond geometry (Å, °) for **10aa** [Chem scheme1]

*D*—H⋯*A*	*D*—H	H⋯*A*	*D*⋯*A*	*D*—H⋯*A*
C3—H3⋯Cl3	1.00	2.70	3.427 (3)	130
C3—H3⋯Cl2^i^	1.00	2.95	3.665 (3)	129
C21—H21*C*⋯Cl2^ii^	0.98	2.99	3.706 (4)	131
C22—H22*A*⋯Cl2^ii^	0.98	2.94	3.675 (4)	133
C13—H13*C*⋯Cl3^iii^	0.98	2.99	3.832 (4)	145

Symmetry codes: (i) 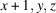 ; (ii) 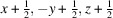 ; (iii) 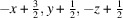 .

**Table 18:** Hydrogen-bond geometry (Å, °) for **11a** [Chem scheme1]

*D*—H⋯*A*	*D*—H	H⋯*A*	*D*⋯*A*	*D*—H⋯*A*
C13—H13*A*⋯Au1	0.98	2.71	3.6142 (19)	154
C12—H12*C*⋯S1	0.98	2.86	3.391 (2)	115
C3—H3⋯Cl3	1.00	2.62	3.451 (2)	140
C12—H12*C*⋯Cl2	0.98	2.81	3.788 (2)	174
C13—H13*C*⋯Cl2^i^	0.98	2.91	3.851 (2)	161

Symmetry code: (i) 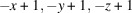 .

**Table 19:** Hydrogen-bond geometry (Å, °) for **11aa** [Chem scheme1]

*D*—H⋯*A*	*D*—H	H⋯*A*	*D*⋯*A*	*D*—H⋯*A*
C13—H13*B*⋯Au1	0.98	2.68	3.6027 (19)	156
C21—H21*A*⋯S1	0.98	2.63	3.1082 (19)	110
C12—H12*C*⋯S1	0.98	2.86	3.411 (2)	116
C3—H3⋯Cl3	1.00	2.65	3.4471 (18)	137
C12—H12*C*⋯Cl2	0.98	2.78	3.755 (2)	171
C99—D99⋯Cl1	1.00	2.74	3.537 (2)	137
C99—D99⋯Cl2	1.00	2.69	3.489 (2)	137
C12—H12*A*⋯Cl1^i^	0.98	2.91	3.596 (2)	128

Symmetry code: (i) 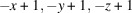 .

**Table 20:** Hydrogen-bond geometry (Å, °) for **12a** [Chem scheme1]

*D*—H⋯*A*	*D*—H	H⋯*A*	*D*⋯*A*	*D*—H⋯*A*
C23—H23*A*⋯Au1	0.98	2.69	3.438 (2)	134
C13—H13*B*⋯S1	0.98	2.61	3.131 (2)	114
C32—H32*A*⋯S1	0.98	2.83	3.323 (2)	112
C23—H23*A*⋯Cl2	0.98	2.82	3.778 (2)	168
C32—H32*A*⋯Cl3	0.98	2.88	3.759 (2)	150
C33—H33*B*⋯Cl3	0.98	2.73	3.623 (2)	152
C99—H99*A*⋯Cl2	0.99	2.84	3.749 (3)	153
C99—H99*B*⋯Cl3^i^	0.99	2.96	3.903 (3)	160
C22—H22*A*⋯Cl3^i^	0.98	2.82	3.791 (2)	171

Symmetry code: (i) 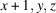 .

**Table 21:** Hydrogen-bond geometry (Å, °) for **14a** [Chem scheme1]

*D*—H⋯*A*	*D*—H	H⋯*A*	*D*⋯*A*	*D*—H⋯*A*
C3—H3⋯Cl3	1.00	2.74	3.445 (9)	128
C3—H3⋯Cl2^i^	1.00	2.99	3.714 (8)	130
C21—H21*C*⋯Cl2^ii^	0.98	2.98	3.730 (10)	135
C22—H22*A*⋯Cl2^ii^	0.98	2.94	3.646 (11)	130
C13—H13*C*⋯Cl3^iii^	0.98	2.98	3.860 (10)	151

Symmetry codes: (i) 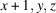 ; (ii) 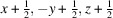 ; (iii) 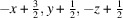 .

**Table 22:** Hydrogen-bond geometry (Å, °) for **15a** [Chem scheme1]

*D*—H⋯*A*	*D*—H	H⋯*A*	*D*⋯*A*	*D*—H⋯*A*
C13—H13*A*⋯Au1	0.98	2.76	3.6818 (18)	158
C21—H21*C*⋯Se1	0.98	2.68	3.1887 (19)	113
C12—H12*C*⋯Se1	0.98	2.92	3.4566 (18)	116
C3—H3⋯Cl3	1.00	2.65	3.4842 (19)	141
C12—H12*C*⋯Cl2	0.98	2.94	3.9145 (19)	174
C13—H13*C*⋯Cl2^i^	0.98	2.93	3.8489 (19)	157

Symmetry code: (i) 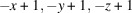 .

**Table 23:** Hydrogen-bond geometry (Å, °) for **15aa** [Chem scheme1]

*D*—H⋯*A*	*D*—H	H⋯*A*	*D*⋯*A*	*D*—H⋯*A*
C99—D99⋯Cl2	1.00	2.76	3.580 (7)	139
C13—H13*A*⋯Au1	0.98	2.69	3.618 (3)	158
C21—H21*C*⋯Se1	0.98	2.70	3.208 (3)	112
C3—H3⋯Cl3	1.00	2.65	3.497 (3)	142
C13—H13*C*⋯Cl2^i^	0.98	2.92	3.866 (3)	163

Symmetry code: (i) 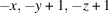 .

**Table 24:** Hydrogen-bond geometry (Å, °) for **9b** [Chem scheme1]

*D*—H⋯*A*	*D*—H	H⋯*A*	*D*⋯*A*	*D*—H⋯*A*
C32—H32*C*⋯Au1	0.98	2.76	3.473 (3)	131
C12—H12*B*⋯S1	0.98	2.68	3.261 (3)	118
C2—H2⋯Br3	1.00	2.71	3.560 (3)	143
C22—H22*B*⋯Au1^i^	0.98	2.98	3.551 (3)	119
C21—H21*C*⋯Br1^ii^	0.98	3.02	3.829 (3)	141
C3—H3⋯Br2^i^	1.00	2.91	3.796 (3)	148
C32—H32*A*⋯Br2^iii^	0.98	3.06	3.900 (3)	145
C11—H11*C*⋯Br2^iv^	0.98	2.91	3.661 (3)	134

Symmetry codes: (i) 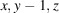 ; (ii) 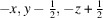 ; (iii) 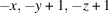 ; (iv) 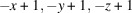 .

**Table 25:** Hydrogen-bond geometry (Å, °) for **11b** [Chem scheme1]

*D*—H⋯*A*	*D*—H	H⋯*A*	*D*⋯*A*	*D*—H⋯*A*
C13—H13*B*⋯Au1	0.98	2.69	3.607 (2)	156
C21—H21*A*⋯S1	0.98	2.63	3.109 (2)	110
C12—H12*A*⋯S1	0.98	2.89	3.417 (2)	114
C3—H3⋯Br3	1.00	2.71	3.546 (2)	141
C12—H12*A*⋯Br2	0.98	2.89	3.863 (2)	174
C13—H13*A*⋯Br2^i^	0.98	3.00	3.931 (2)	159

Symmetry code: (i) 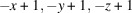 .

**Table 26:** Hydrogen-bond geometry (Å, °) for **13b** [Chem scheme1]

*D*—H⋯*A*	*D*—H	H⋯*A*	*D*⋯*A*	*D*—H⋯*A*
C32—H32*C*⋯Au1	0.98	2.77	3.578 (5)	140
C12—H12*B*⋯Se1	0.98	2.90	3.479 (5)	119
C2—H2⋯Br3	1.00	2.71	3.497 (4)	136
C11—H11*C*⋯Br3^i^	0.98	2.78	3.752 (5)	171
C32—H32*A*⋯Br2^ii^	0.98	2.99	3.933 (4)	163

Symmetry codes: (i) 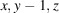 ; (ii) 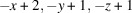 .

**Table 27:** Hydrogen-bond geometry (Å, °) for **15b** [Chem scheme1]

*D*—H⋯*A*	*D*—H	H⋯*A*	*D*⋯*A*	*D*—H⋯*A*
C13—H13*B*⋯Au1	0.98	2.74	3.679 (3)	160
C21—H21*A*⋯Se1	0.98	2.69	3.197 (3)	113
C12—H12*A*⋯Se1	0.98	2.96	3.484 (3)	115
C3—H3⋯Br3	1.00	2.75	3.585 (3)	142
C12—H12*A*⋯Br2	0.98	3.01	3.980 (3)	173
C13—H13*A*⋯Br2^i^	0.98	3.02	3.929 (3)	156

Symmetry code: (i) 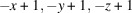 .

**Table:** 

	**9a**	**10a**	**10aa**	**11a**	**11aa**
Crystal data					
Chemical formula	[AuCl_3_(C_9_H_21_PS)]	[AuCl_3_(C_10_H_23_PS)]	[AuCl_3_(C_10_H_23_PS)]	[AuCl_3_(C_11_H_25_PS)]	[AuCl_3_(C_11_H_25_PS)]·CDCl_3_
*M*_r_	495.60	509.63	509.63	523.66	644.03
Crystal system, space group	Triclinic, *P* 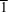	Monoclinic, *P*2_1_/*n*	Monoclinic, *P*2_1_/*n*	Triclinic, *P* 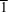	Triclinic, *P* 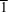
Temperature (K)	100	100	100	100	101
*a*, *b*, *c* (Å)	8.0262 (3), 9.0839 (3), 10.7162 (3)	8.4533 (2), 17.0563 (4), 11.4826 (3)	7.9363 (3), 14.4096 (4), 14.2851 (4)	8.6034 (4), 9.7779 (4), 11.4231 (4)	9.6382 (4), 10.2787 (3), 11.8483 (5)
α, β, γ (°)	86.185 (2), 85.730 (3), 84.468 (3)	90, 94.525 (2), 90	90, 91.774 (3), 90	78.876 (3), 71.456 (4), 72.702 (4)	75.115 (3), 68.875 (4), 89.728 (3)
*V* (Å^3^)	774.13 (4)	1650.43 (7)	1632.85 (9)	864.69 (7)	1053.13 (8)
*Z*	2	4	4	2	2
Radiation type	Mo *K*α	Mo *K*α	Mo *K*α	Mo *K*α	Mo *K*α
μ (mm^−1^)	10.23	9.60	9.70	9.16	7.91
Crystal size (mm)	0.22 × 0.05 × 0.01	0.3 × 0.2 × 0.02	0.3 × 0.2 × 0.2	0.15 × 0.15 × 0.08	0.15 × 0.06 × 0.05

Data collection					
Diffractometer	Oxford Diffraction Xcalibur, Eos	Oxford Diffraction Xcalibur, Eos	Oxford Diffraction Xcalibur, Eos	Oxford Diffraction Xcalibur, Eos	Oxford Diffraction Xcalibur, Eos
Absorption correction	Multi-scan (*CrysAlis PRO*; Rigaku OD, 2020 ▸)	Multi-scan (*CrysAlis PRO*; Rigaku OD, 2020 ▸)	Multi-scan (*CrysAlis PRO*; Rigaku OD, 2020 ▸)	Multi-scan (*CrysAlis PRO*; Rigaku OD, 2020 ▸)	Multi-scan (*CrysAlis PRO*; Rigaku OD, 2020 ▸)
*T*_min_, *T*_max_	0.421, 1.000	0.192, 1.000	0.159, 0.247	0.577, 1.000	0.454, 1.000
No. of measured, independent and observed [*I* > 2σ(*I*)] reflections	56439, 4632, 4428	40041, 4863, 4483	42542, 4881, 4296	68815, 5155, 4939	76947, 6247, 5976
*R*_int_	0.037	0.040	0.046	0.038	0.038
(sin θ/λ)_max_ (Å^−1^)	0.720	0.722	0.721	0.721	0.723

Refinement					
*R*[*F* ^2^ > 2σ(*F* ^2^)], *wR*(*F* ^2^), *S*	0.015, 0.033, 1.05	0.021, 0.046, 1.07	0.025, 0.045, 1.12	0.016, 0.036, 1.08	0.016, 0.033, 1.05
No. of reflections	4632	4863	4881	5155	6247
No. of parameters	142	152	152	163	199
No. of restraints	0	0	0	0	0
H-atom treatment	H-atom parameters constrained	H-atom parameters constrained	H-atom parameters constrained	H-atom parameters constrained	H-atom parameters constrained
Δρ_max_, Δρ_min_ (e Å^−3^)	1.05, −0.96	2.15, −1.41	2.52, −1.50	1.64, −0.93	1.02, −0.95
Extinction method	None	None	None	*F*_c_^*^ = *kF*_c_[1 + 0.001 *F*_c_^2^λ^3^/sin(2θ)]^-1/4^ (*SHELXL2019/3*; Sheldrick, 2015 ▸)	*F*_c_^*^ = *kF*_c_[1 + 0.001 *F*_c_^2^λ^3^/sin(2θ)]^-1/4^ (*SHELXL2019/3*; Sheldrick, 2015 ▸)
Extinction coefficient	–	–	–	0.00097 (14)	0.00133 (9)

**Table:** 

	**12a**	**14a**	**15a**	**15aa**	**9b**
Crystal data					
Chemical formula	[AuCl_3_(C_12_H_27_PS)]·CH_2_Cl_2_	[AuCl_3_(C_10_H_23_PSe)]	[AuCl_3_(C_11_H_25_PSe)]	[AuCl_3_(C_11_H_25_PSe)]·CDCl_3_	[AuBr_3_(C_10_H_23_PS)]
*M*_r_	622.61	556.53	570.55	690.93	628.98
Crystal system, space group	Triclinic, *P* 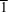	Monoclinic, *P*2_1_/*n*	Triclinic, *P* 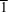	Triclinic, *P* 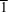	Monoclinic, *P*2_1_/*c*
Temperature (K)	100	100	100	100	100
*a*, *b*, *c* (Å)	8.4202 (3), 11.2194 (4), 11.8355 (4)	7.92516 (18), 14.5559 (4), 14.3635 (4)	8.5878 (4), 9.8435 (4), 11.5022 (5)	8.5343 (2), 9.7185 (3), 14.0759 (4)	9.1341 (2), 7.9039 (2), 22.6420 (4)
α, β, γ (°)	98.398 (3), 101.174 (3), 95.991 (3)	90, 91.264 (2), 90	78.391 (3), 71.168 (4), 73.463 (4)	74.398 (2), 78.121 (2), 73.257 (2)	90, 94.519 (2), 90
*V* (Å^3^)	1074.95 (7)	1656.54 (7)	875.78 (7)	1066.31 (5)	1629.56 (6)
*Z*	2	4	2	2	4
Radiation type	Mo *K*α	Mo *K*α	Mo *K*α	Mo *K*α	Mo *K*α
μ (mm^−1^)	7.63	11.64	11.01	9.42	16.58
Crystal size (mm)	0.15 × 0.1 × 0.1	0.15 × 0.15 × 0.1	0.18 × 0.15 × 0.12	0.4 × 0.25 × 0.08	0.15 × 0.1 × 0.1

Data collection					
Diffractometer	Oxford Diffraction Xcalibur, Eos	Oxford Diffraction Xcalibur, Eos	Oxford Diffraction Xcalibur, Eos	Oxford Diffraction Xcalibur, Eos	Oxford Diffraction Xcalibur, Eos
Absorption correction	Multi-scan (*CrysAlis PRO*; Rigaku OD, 2020 ▸)	Multi-scan (*CrysAlis PRO*; Rigaku OD, 2020 ▸)	Multi-scan (*CrysAlis PRO*; Rigaku OD, 2020 ▸)	Multi-scan (*CrysAlis PRO*; Rigaku OD, 2020 ▸)	Multi-scan (*CrysAlis PRO*; Rigaku OD, 2020 ▸)
*T*_min_, *T*_max_	0.783, 1.000	0.483, 1.000	0.700, 1.000	0.151, 1.000	0.486, 1.000
No. of measured, independent and observed [*I* > 2σ(*I*)] reflections	157605, 6558, 6124	196706, 4112, 3878	54532, 5231, 4984	76069, 6287, 6060	64266, 4964, 4633
*R*_int_	0.054	0.084	0.032	0.040	0.037
(sin θ/λ)_max_ (Å^−1^)	0.727	0.667	0.722	0.721	0.722

Refinement					
*R*[*F* ^2^ > 2σ(*F* ^2^)], *wR*(*F* ^2^), *S*	0.021, 0.037, 1.05	0.043, 0.093, 1.32	0.014, 0.030, 1.10	0.021, 0.048, 1.10	0.022, 0.038, 1.23
No. of reflections	6558	4112	5231	6287	4964
No. of parameters	199	152	163	235	143
No. of restraints	0	0	0	39	0
H-atom treatment	H-atom parameters constrained	H-atom parameters constrained	H-atom parameters constrained	H-atom parameters constrained	H-atom parameters constrained
Δρ_max_, Δρ_min_ (e Å^−3^)	1.76, −1.01	3.81, −2.41	0.84, −0.73	1.88, −1.30	1.58, −0.95
Extinction method	None	None	*F*_c_^*^ = *kF*_c_[1 + 0.001 *F*_c_^2^λ^3^/sin(2θ)]^-1/4^ (*SHELXL2019/3*; Sheldrick, 2015 ▸)	None	*F*_c_^*^ = *kF*_c_[1 + 0.001 *F*_c_^2^λ^3^/sin(2θ)]^-1/4^ (*SHELXL2019/3*; Sheldrick, 2015 ▸)
Extinction coefficient	–	–	0.00113 (8)	–	0.00043 (2)

**Table:** 

	**11b**	**13b**	**15b**
Crystal data			
Chemical formula	[AuBr_3_(C_11_H_25_PS)]	[AuBr_3_(C_9_H_21_PSe)]	[AuBr_3_(C_11_H_25_PSe)]
*M*_r_	657.04	675.88	703.93
Crystal system, space group	Triclinic, *P* 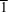	Triclinic, *P* 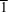	Triclinic, *P* 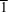
Temperature (K)	100	100	100
*a*, *b*, *c* (Å)	8.6067 (8), 10.1161 (12), 11.5123 (12)	8.3928 (2), 10.1417 (4), 10.7567 (4)	8.6000 (5), 10.2045 (7), 11.5987 (7)
α, β, γ (°)	77.873 (10), 70.257 (10), 71.867 (10)	94.419 (3), 105.612 (3), 110.113 (3)	77.475 (6), 69.764 (6), 72.601 (6)
*V* (Å^3^)	890.37 (18)	813.33 (5)	904.02 (11)
*Z*	2	2	2
Radiation type	Mo *K*α	Mo *K*α	Mo *K*α
μ (mm^−1^)	15.18	18.72	16.85
Crystal size (mm)	0.2 × 0.2 × 0.2	0.2 × 0.1 × 0.01	0.2 × 0.06 × 0.04

Data collection			
Diffractometer	Oxford Diffaction Xcalibur, Eos	Oxford Diffraction Xcalibur, Eos	Oxford Diffraction Xcalibur, Eos
Absorption correction	Multi-scan (*CrysAlis PRO*; Rigaku OD, 2020 ▸)	Multi-scan (*CrysAlis PRO*; Rigaku OD, 2020 ▸)	Multi-scan (*CrysAlis PRO*; Rigaku OD, 2020 ▸)
*T*_min_, *T*_max_	0.447, 1.000	0.200, 1.000	0.376, 1.000
No. of measured, independent and observed [*I* > 2σ(*I*)] reflections	65025, 5297, 5057	44535, 4825, 4278	25566, 5282, 4719
*R*_int_	0.038	0.059	0.033
(sin θ/λ)_max_ (Å^−1^)	0.721	0.724	0.721

Refinement			
*R*[*F* ^2^ > 2σ(*F* ^2^)], *wR*(*F* ^2^), *S*	0.017, 0.036, 1.14	0.028, 0.074, 1.05	0.020, 0.036, 1.05
No. of reflections	5297	4825	5282
No. of parameters	163	142	163
No. of restraints	0	0	0
H-atom treatment	H-atom parameters constrained	H-atom parameters constrained	H-atom parameters constrained
Δρ_max_, Δρ_min_ (e Å^−3^)	1.08, −1.43	1.43, −1.49	1.04, −0.80
Extinction method	*F*_c_^*^ = *kF*_c_[1 + 0.001 *F*_c_^2^λ^3^/sin(2θ)]^-1/4^ (*SHELXL2019/3*; Sheldrick, 2015 ▸)	None	*F*_c_^*^ = *kF*_c_[1 + 0.001 *F*_c_^2^λ^3^/sin(2θ)]^-1/4^ (*SHELXL2019/3*; Sheldrick, 2015 ▸)
Extinction coefficient	0.00557 (11)	–	0.00115 (6)

Computer programs: *CrysAlis PRO* (Rigaku OD, 2020 ▸), *SHELXS97* (Sheldrick, 2008 ▸), *SHELXL2019/3* (Sheldrick, 2015 ▸) and *XP* (Bruker, 1998 ▸).

## supplementary crystallographic information

### Trichlorido(tripropan-2-ylphosphane sulfide-κS)gold(III) (9a) Crystal data

**Table:**

[AuCl_3_(C_9_H_21_PS)]	*Z* = 2
*M**_r_* = 495.60	*F*(000) = 472
Triclinic, *P*1	*D*_x_ = 2.126 Mg m^−^^3^
*a* = 8.0262 (3) Å	Mo *K*α radiation, λ = 0.71073 Å
*b* = 9.0839 (3) Å	Cell parameters from 25652 reflections
*c* = 10.7162 (3) Å	θ = 2.3–30.7°
α = 86.185 (2)°	µ = 10.23 mm^−^^1^
β = 85.730 (3)°	*T* = 100 K
γ = 84.468 (3)°	Plate, dichroic orange / yellow
*V* = 774.13 (4) Å^3^	0.22 × 0.05 × 0.01 mm

### Trichlorido(tripropan-2-ylphosphane sulfide-κS)gold(III) (9a) Data collection

**Table:**

Oxford Diffraction Xcalibur, Eos diffractometer	4632 independent reflections
Radiation source: Enhance (Mo) X-ray source	4428 reflections with *I* > 2σ(*I*)
Detector resolution: 16.1419 pixels mm^-1^	*R*_int_ = 0.037
ω scans	θ_max_ = 30.8°, θ_min_ = 2.3°
Absorption correction: multi-scan (CrysAlisPro; Rigaku OD, 2020)	*h* = −11→11
*T*_min_ = 0.421, *T*_max_ = 1.000	*k* = −12→12
56439 measured reflections	*l* = −15→15

### Trichlorido(tripropan-2-ylphosphane sulfide-κS)gold(III) (9a) Refinement

**Table:**

Refinement on *F*^2^	Primary atom site location: structure-invariant direct methods
Least-squares matrix: full	Secondary atom site location: difference Fourier map
*R*[*F*^2^ > 2σ(*F*^2^)] = 0.015	Hydrogen site location: inferred from neighbouring sites
*wR*(*F*^2^) = 0.033	H-atom parameters constrained
*S* = 1.05	*w* = 1/[σ^2^(*F*_o_^2^) + (0.0167*P*)^2^ + 0.4566*P*] where *P* = (*F*_o_^2^ + 2*F*_c_^2^)/3
4632 reflections	(Δ/σ)_max_ = 0.002
142 parameters	Δρ_max_ = 1.05 e Å^−^^3^
0 restraints	Δρ_min_ = −0.96 e Å^−^^3^

### Trichlorido(tripropan-2-ylphosphane sulfide-κS)gold(III) (9a) Fractional atomic coordinates and isotropic or equivalent isotropic displacement parameters (Å^2^)

**Table:**

	*x*	*y*	*z*	*U*_iso_*/*U*_eq_	
Au1	0.74127 (2)	0.15601 (2)	0.24499 (2)	0.01058 (3)	
Cl1	0.90254 (7)	−0.06826 (5)	0.24307 (5)	0.02024 (10)	
Cl3	0.67060 (6)	0.11336 (6)	0.45357 (5)	0.01749 (9)	
Cl2	0.83231 (6)	0.20433 (6)	0.04060 (4)	0.01903 (10)	
S1	0.58505 (6)	0.38531 (5)	0.23869 (5)	0.01309 (9)	
P1	0.33654 (6)	0.34414 (5)	0.25253 (4)	0.01036 (9)	
C1	0.2343 (2)	0.5273 (2)	0.20553 (19)	0.0136 (4)	
H1	0.278877	0.550333	0.117475	0.016*	
C2	0.2705 (2)	0.2697 (2)	0.40925 (18)	0.0136 (4)	
H2	0.342680	0.174844	0.423351	0.016*	
C3	0.2864 (3)	0.2158 (2)	0.13891 (19)	0.0152 (4)	
H3	0.161322	0.217320	0.143959	0.018*	
C11	0.0440 (3)	0.5277 (2)	0.2004 (2)	0.0235 (5)	
H11A	−0.008072	0.516636	0.285805	0.035*	
H11B	0.019245	0.445182	0.152445	0.035*	
H11C	−0.001015	0.621446	0.159793	0.035*	
C12	0.2796 (3)	0.6527 (2)	0.2818 (2)	0.0257 (5)	
H12A	0.238861	0.748320	0.241944	0.039*	
H12B	0.401672	0.647672	0.285129	0.039*	
H12C	0.227092	0.642306	0.367069	0.039*	
C21	0.3038 (3)	0.3672 (2)	0.5135 (2)	0.0214 (4)	
H21A	0.225900	0.457144	0.510729	0.032*	
H21B	0.419396	0.394310	0.501883	0.032*	
H21C	0.287352	0.312734	0.594864	0.032*	
C22	0.0893 (3)	0.2277 (3)	0.4200 (2)	0.0211 (4)	
H22A	0.066095	0.175991	0.501716	0.032*	
H22B	0.073806	0.162680	0.353195	0.032*	
H22C	0.012040	0.317689	0.411899	0.032*	
C31	0.3395 (3)	0.2667 (3)	0.0039 (2)	0.0232 (5)	
H31A	0.462041	0.264402	−0.006264	0.035*	
H31B	0.289704	0.367907	−0.014347	0.035*	
H31C	0.300690	0.200277	−0.053941	0.035*	
C32	0.3543 (3)	0.0551 (2)	0.1703 (2)	0.0203 (4)	
H32A	0.306801	−0.010584	0.116174	0.030*	
H32B	0.322688	0.027578	0.258217	0.030*	
H32C	0.476851	0.045590	0.156468	0.030*	

### Trichlorido(tripropan-2-ylphosphane sulfide-κS)gold(III) (9a) Atomic displacement parameters (Å^2^)

**Table:**

	*U*^11^	*U*^22^	*U*^33^	*U*^12^	*U*^13^	*U*^23^
Au1	0.00962 (4)	0.01142 (4)	0.01073 (4)	−0.00084 (2)	−0.00111 (2)	−0.00061 (2)
Cl1	0.0213 (2)	0.0167 (2)	0.0215 (2)	0.00572 (18)	−0.00174 (19)	−0.00262 (19)
Cl3	0.0157 (2)	0.0213 (2)	0.0138 (2)	0.00148 (17)	0.00099 (17)	0.00419 (18)
Cl2	0.0198 (2)	0.0250 (2)	0.0116 (2)	−0.00060 (19)	0.00136 (17)	−0.00064 (18)
S1	0.0115 (2)	0.0108 (2)	0.0169 (2)	−0.00201 (16)	−0.00067 (17)	0.00050 (17)
P1	0.0103 (2)	0.0095 (2)	0.0114 (2)	−0.00047 (16)	−0.00153 (17)	−0.00085 (17)
C1	0.0149 (9)	0.0102 (8)	0.0152 (9)	0.0012 (7)	−0.0019 (7)	0.0012 (7)
C2	0.0120 (9)	0.0155 (9)	0.0131 (9)	−0.0022 (7)	−0.0006 (7)	0.0012 (7)
C3	0.0135 (9)	0.0157 (9)	0.0172 (9)	−0.0004 (7)	−0.0033 (7)	−0.0061 (7)
C11	0.0144 (10)	0.0197 (10)	0.0344 (13)	0.0032 (8)	−0.0019 (9)	0.0055 (9)
C12	0.0358 (13)	0.0118 (9)	0.0303 (12)	0.0047 (9)	−0.0124 (10)	−0.0050 (8)
C21	0.0293 (12)	0.0221 (10)	0.0131 (9)	−0.0061 (9)	0.0016 (8)	−0.0023 (8)
C22	0.0141 (9)	0.0290 (11)	0.0200 (10)	−0.0058 (8)	−0.0013 (8)	0.0062 (9)
C31	0.0294 (12)	0.0268 (11)	0.0147 (10)	−0.0027 (9)	−0.0048 (9)	−0.0057 (8)
C32	0.0228 (10)	0.0131 (9)	0.0264 (11)	−0.0026 (8)	−0.0044 (9)	−0.0070 (8)

### Trichlorido(tripropan-2-ylphosphane sulfide-κS)gold(III) (9a) Geometric parameters (Å, º)

**Table:**

Au1—Cl3	2.2818 (5)	C11—H11B	0.9800
Au1—Cl2	2.2846 (5)	C11—H11C	0.9800
Au1—Cl1	2.3064 (5)	C12—H12A	0.9800
Au1—S1	2.3250 (5)	C12—H12B	0.9800
S1—P1	2.0574 (7)	C12—H12C	0.9800
P1—C2	1.829 (2)	C21—H21A	0.9800
P1—C3	1.8317 (19)	C21—H21B	0.9800
P1—C1	1.8387 (19)	C21—H21C	0.9800
C1—C12	1.531 (3)	C22—H22A	0.9800
C1—C11	1.532 (3)	C22—H22B	0.9800
C1—H1	1.0000	C22—H22C	0.9800
C2—C21	1.525 (3)	C31—H31A	0.9800
C2—C22	1.534 (3)	C31—H31B	0.9800
C2—H2	1.0000	C31—H31C	0.9800
C3—C31	1.531 (3)	C32—H32A	0.9800
C3—C32	1.533 (3)	C32—H32B	0.9800
C3—H3	1.0000	C32—H32C	0.9800
C11—H11A	0.9800		
			
Cl3—Au1—Cl2	175.341 (18)	C1—C11—H11C	109.5
Cl3—Au1—Cl1	90.151 (18)	H11A—C11—H11C	109.5
Cl2—Au1—Cl1	88.999 (19)	H11B—C11—H11C	109.5
Cl3—Au1—S1	92.249 (18)	C1—C12—H12A	109.5
Cl2—Au1—S1	88.545 (18)	C1—C12—H12B	109.5
Cl1—Au1—S1	177.472 (17)	H12A—C12—H12B	109.5
P1—S1—Au1	106.70 (2)	C1—C12—H12C	109.5
C2—P1—C3	108.01 (9)	H12A—C12—H12C	109.5
C2—P1—C1	115.39 (9)	H12B—C12—H12C	109.5
C3—P1—C1	106.80 (9)	C2—C21—H21A	109.5
C2—P1—S1	111.92 (7)	C2—C21—H21B	109.5
C3—P1—S1	113.32 (7)	H21A—C21—H21B	109.5
C1—P1—S1	101.34 (7)	C2—C21—H21C	109.5
C12—C1—C11	111.53 (18)	H21A—C21—H21C	109.5
C12—C1—P1	113.93 (14)	H21B—C21—H21C	109.5
C11—C1—P1	112.89 (14)	C2—C22—H22A	109.5
C12—C1—H1	105.9	C2—C22—H22B	109.5
C11—C1—H1	105.9	H22A—C22—H22B	109.5
P1—C1—H1	105.9	C2—C22—H22C	109.5
C21—C2—C22	111.88 (17)	H22A—C22—H22C	109.5
C21—C2—P1	113.49 (14)	H22B—C22—H22C	109.5
C22—C2—P1	113.14 (14)	C3—C31—H31A	109.5
C21—C2—H2	105.8	C3—C31—H31B	109.5
C22—C2—H2	105.8	H31A—C31—H31B	109.5
P1—C2—H2	105.8	C3—C31—H31C	109.5
C31—C3—C32	111.51 (17)	H31A—C31—H31C	109.5
C31—C3—P1	112.32 (14)	H31B—C31—H31C	109.5
C32—C3—P1	113.08 (14)	C3—C32—H32A	109.5
C31—C3—H3	106.5	C3—C32—H32B	109.5
C32—C3—H3	106.5	H32A—C32—H32B	109.5
P1—C3—H3	106.5	C3—C32—H32C	109.5
C1—C11—H11A	109.5	H32A—C32—H32C	109.5
C1—C11—H11B	109.5	H32B—C32—H32C	109.5
H11A—C11—H11B	109.5		
			
Cl3—Au1—S1—P1	−72.41 (3)	C1—P1—C2—C21	−60.06 (17)
Cl2—Au1—S1—P1	112.19 (3)	S1—P1—C2—C21	55.13 (16)
Au1—S1—P1—C2	70.57 (7)	C3—P1—C2—C22	−50.58 (17)
Au1—S1—P1—C3	−51.87 (8)	C1—P1—C2—C22	68.81 (17)
Au1—S1—P1—C1	−165.92 (7)	S1—P1—C2—C22	−176.00 (13)
C2—P1—C1—C12	66.59 (18)	C2—P1—C3—C31	−179.04 (14)
C3—P1—C1—C12	−173.36 (16)	C1—P1—C3—C31	56.26 (17)
S1—P1—C1—C12	−54.51 (16)	S1—P1—C3—C31	−54.47 (16)
C2—P1—C1—C11	−61.96 (17)	C2—P1—C3—C32	−51.75 (17)
C3—P1—C1—C11	58.09 (17)	C1—P1—C3—C32	−176.45 (15)
S1—P1—C1—C11	176.93 (14)	S1—P1—C3—C32	72.83 (16)
C3—P1—C2—C21	−179.45 (15)		

### Trichlorido(tripropan-2-ylphosphane sulfide-κS)gold(III) (9a) Hydrogen-bond geometry (Å, º)

**Table:**

*D*—H···*A*	*D*—H	H···*A*	*D*···*A*	*D*—H···*A*
C32—H32*C*···Au1	0.98	2.69	3.485 (2)	138
C2—H2···Cl3	1.00	2.68	3.437 (2)	133
C12—H12*B*···S1	0.98	2.73	3.310 (2)	118
C1—H1···Cl2^i^	1.00	2.82	3.506 (2)	126
C11—H11*C*···Cl2^i^	0.98	2.91	3.582 (2)	127
C22—H22*A*···Cl1^ii^	0.98	2.86	3.803 (2)	161
C2—H2···Cl3^ii^	1.00	2.86	3.688 (2)	141
C32—H32*A*···Cl2^iii^	0.98	2.87	3.847 (2)	180
C3—H3···Cl2^iv^	1.00	2.95	3.881 (2)	155

Symmetry codes: (i) −*x*+1, −*y*+1, −*z*; (ii) −*x*+1, −*y*, −*z*+1; (iii) −*x*+1, −*y*, −*z*; (iv) *x*−1, *y*, *z*.

### (tert-Butyldipropan-2-ylphosphane sulfide-κS)trichloridogold(III) (10a) Crystal data

**Table:**

[AuCl_3_(C_10_H_23_PS)]	*F*(000) = 976
*M**_r_* = 509.63	*D*_x_ = 2.051 Mg m^−^^3^
Monoclinic, *P*2_1_/*n*	Mo *K*α radiation, λ = 0.71073 Å
*a* = 8.4533 (2) Å	Cell parameters from 16512 reflections
*b* = 17.0563 (4) Å	θ = 2.1–30.8°
*c* = 11.4826 (3) Å	µ = 9.60 mm^−^^1^
β = 94.525 (2)°	*T* = 100 K
*V* = 1650.43 (7) Å^3^	Plate, dichroic red / orange
*Z* = 4	0.3 × 0.2 × 0.02 mm

### (tert-Butyldipropan-2-ylphosphane sulfide-κS)trichloridogold(III) (10a) Data collection

**Table:**

Oxford Diffraction Xcalibur, Eos diffractometer	4863 independent reflections
Radiation source: Enhance (Mo) X-ray Source	4483 reflections with *I* > 2σ(*I*)
Detector resolution: 16.1419 pixels mm^-1^	*R*_int_ = 0.040
ω scan	θ_max_ = 30.9°, θ_min_ = 2.4°
Absorption correction: multi-scan (CrysAlisPro; Rigaku OD, 2020)	*h* = −12→11
*T*_min_ = 0.192, *T*_max_ = 1.000	*k* = −24→23
40041 measured reflections	*l* = −15→16

### (tert-Butyldipropan-2-ylphosphane sulfide-κS)trichloridogold(III) (10a) Refinement

**Table:**

Refinement on *F*^2^	Primary atom site location: structure-invariant direct methods
Least-squares matrix: full	Secondary atom site location: difference Fourier map
*R*[*F*^2^ > 2σ(*F*^2^)] = 0.021	Hydrogen site location: inferred from neighbouring sites
*wR*(*F*^2^) = 0.046	H-atom parameters constrained
*S* = 1.07	*w* = 1/[σ^2^(*F*_o_^2^) + (0.0188*P*)^2^ + 1.7957*P*] where *P* = (*F*_o_^2^ + 2*F*_c_^2^)/3
4863 reflections	(Δ/σ)_max_ = 0.004
152 parameters	Δρ_max_ = 2.15 e Å^−^^3^
0 restraints	Δρ_min_ = −1.41 e Å^−^^3^

### (tert-Butyldipropan-2-ylphosphane sulfide-κS)trichloridogold(III) (10a) Fractional atomic coordinates and isotropic or equivalent isotropic displacement parameters (Å^2^)

**Table:**

	*x*	*y*	*z*	*U*_iso_*/*U*_eq_	
Au1	0.53732 (2)	0.45811 (2)	0.69892 (2)	0.01252 (3)	
Cl1	0.60148 (8)	0.33007 (4)	0.74376 (6)	0.02142 (13)	
Cl2	0.78118 (7)	0.49571 (4)	0.77945 (6)	0.01953 (13)	
Cl3	0.29411 (8)	0.41592 (4)	0.62396 (6)	0.01961 (13)	
S1	0.49553 (7)	0.58842 (4)	0.64406 (6)	0.01545 (12)	
P1	0.29578 (8)	0.63290 (4)	0.71079 (5)	0.01203 (12)	
C1	0.2845 (3)	0.61053 (17)	0.8698 (2)	0.0175 (5)	
C2	0.3186 (3)	0.73939 (16)	0.6906 (2)	0.0181 (5)	
H2	0.225743	0.765191	0.723585	0.022*	
C3	0.1136 (3)	0.59893 (16)	0.6267 (2)	0.0136 (5)	
H3	0.086801	0.546807	0.659853	0.016*	
C11	0.1916 (4)	0.6760 (2)	0.9271 (3)	0.0302 (7)	
H11A	0.087035	0.681955	0.884754	0.045*	
H11B	0.250247	0.725398	0.924447	0.045*	
H11C	0.178412	0.662105	1.008626	0.045*	
C12	0.4522 (3)	0.60526 (18)	0.9316 (2)	0.0210 (6)	
H12A	0.444865	0.599219	1.015825	0.032*	
H12B	0.511144	0.653237	0.916622	0.032*	
H12C	0.507673	0.559959	0.901581	0.032*	
C13	0.2002 (4)	0.53228 (19)	0.8846 (3)	0.0255 (7)	
H13A	0.251077	0.491559	0.840353	0.038*	
H13B	0.088482	0.537125	0.855490	0.038*	
H13C	0.206934	0.517989	0.967533	0.038*	
C21	0.4686 (4)	0.77242 (18)	0.7570 (3)	0.0269 (7)	
H21A	0.562457	0.747474	0.728384	0.040*	
H21B	0.465404	0.761735	0.840654	0.040*	
H21C	0.473607	0.829162	0.744403	0.040*	
C22	0.3144 (4)	0.76266 (18)	0.5614 (3)	0.0255 (6)	
H22A	0.327366	0.819562	0.555111	0.038*	
H22B	0.212447	0.747082	0.521601	0.038*	
H22C	0.400784	0.736199	0.524910	0.038*	
C31	−0.0295 (3)	0.65284 (17)	0.6416 (2)	0.0181 (5)	
H31A	−0.008746	0.704791	0.609683	0.027*	
H31B	−0.046308	0.657606	0.724769	0.027*	
H31C	−0.124461	0.630425	0.599796	0.027*	
C32	0.1375 (3)	0.58531 (16)	0.4971 (2)	0.0165 (5)	
H32A	0.038294	0.566233	0.456976	0.025*	
H32B	0.221306	0.546296	0.490133	0.025*	
H32C	0.168064	0.634707	0.461534	0.025*	

### (tert-Butyldipropan-2-ylphosphane sulfide-κS)trichloridogold(III) (10a) Atomic displacement parameters (Å^2^)

**Table:**

	*U*^11^	*U*^22^	*U*^33^	*U*^12^	*U*^13^	*U*^23^
Au1	0.01036 (5)	0.01403 (6)	0.01301 (5)	−0.00005 (4)	−0.00001 (3)	−0.00017 (3)
Cl1	0.0212 (3)	0.0170 (3)	0.0257 (3)	0.0027 (3)	−0.0009 (2)	0.0029 (3)
Cl2	0.0117 (3)	0.0263 (4)	0.0201 (3)	−0.0019 (3)	−0.0024 (2)	0.0002 (3)
Cl3	0.0141 (3)	0.0168 (3)	0.0270 (3)	−0.0017 (2)	−0.0045 (2)	−0.0010 (3)
S1	0.0124 (3)	0.0152 (3)	0.0192 (3)	−0.0010 (2)	0.0032 (2)	0.0019 (2)
P1	0.0111 (3)	0.0123 (3)	0.0124 (3)	−0.0007 (2)	−0.0011 (2)	−0.0005 (2)
C1	0.0145 (12)	0.0266 (15)	0.0110 (11)	0.0018 (11)	−0.0019 (9)	0.0000 (10)
C2	0.0157 (12)	0.0137 (13)	0.0244 (13)	−0.0018 (10)	−0.0016 (10)	−0.0023 (10)
C3	0.0120 (11)	0.0154 (12)	0.0128 (11)	−0.0020 (10)	−0.0023 (9)	−0.0008 (9)
C11	0.0291 (16)	0.045 (2)	0.0170 (13)	0.0155 (15)	0.0014 (11)	−0.0051 (13)
C12	0.0196 (13)	0.0261 (15)	0.0165 (12)	0.0013 (12)	−0.0041 (10)	−0.0012 (11)
C13	0.0257 (15)	0.0347 (18)	0.0157 (13)	−0.0060 (13)	−0.0006 (11)	0.0074 (12)
C21	0.0222 (15)	0.0180 (14)	0.0392 (17)	−0.0055 (12)	−0.0059 (12)	−0.0050 (13)
C22	0.0287 (16)	0.0194 (15)	0.0284 (15)	−0.0054 (13)	0.0026 (12)	0.0058 (12)
C31	0.0119 (12)	0.0204 (14)	0.0214 (13)	0.0011 (11)	−0.0020 (9)	−0.0029 (10)
C32	0.0185 (13)	0.0181 (13)	0.0124 (11)	−0.0003 (11)	−0.0026 (9)	−0.0005 (10)

### (tert-Butyldipropan-2-ylphosphane sulfide-κS)trichloridogold(III) (10a) Geometric parameters (Å, º)

**Table:**

Au1—Cl3	2.2818 (6)	C11—H11C	0.9800
Au1—Cl2	2.2837 (6)	C12—H12A	0.9800
Au1—Cl1	2.2989 (7)	C12—H12B	0.9800
Au1—S1	2.3294 (7)	C12—H12C	0.9800
S1—P1	2.0538 (9)	C13—H13A	0.9800
P1—C2	1.843 (3)	C13—H13B	0.9800
P1—C3	1.845 (2)	C13—H13C	0.9800
P1—C1	1.875 (3)	C21—H21A	0.9800
C1—C13	1.529 (4)	C21—H21B	0.9800
C1—C12	1.537 (4)	C21—H21C	0.9800
C1—C11	1.542 (4)	C22—H22A	0.9800
C2—C21	1.534 (4)	C22—H22B	0.9800
C2—C22	1.534 (4)	C22—H22C	0.9800
C2—H2	1.0000	C31—H31A	0.9800
C3—C32	1.535 (3)	C31—H31B	0.9800
C3—C31	1.540 (4)	C31—H31C	0.9800
C3—H3	1.0000	C32—H32A	0.9800
C11—H11A	0.9800	C32—H32B	0.9800
C11—H11B	0.9800	C32—H32C	0.9800
			
Cl3—Au1—Cl2	177.46 (2)	C1—C12—H12A	109.5
Cl3—Au1—Cl1	88.54 (2)	C1—C12—H12B	109.5
Cl2—Au1—Cl1	89.23 (3)	H12A—C12—H12B	109.5
Cl3—Au1—S1	94.93 (2)	C1—C12—H12C	109.5
Cl2—Au1—S1	87.39 (2)	H12A—C12—H12C	109.5
Cl1—Au1—S1	174.30 (2)	H12B—C12—H12C	109.5
P1—S1—Au1	111.29 (3)	C1—C13—H13A	109.5
C2—P1—C3	109.58 (12)	C1—C13—H13B	109.5
C2—P1—C1	109.67 (13)	H13A—C13—H13B	109.5
C3—P1—C1	109.94 (12)	C1—C13—H13C	109.5
C2—P1—S1	102.70 (10)	H13A—C13—H13C	109.5
C3—P1—S1	111.45 (9)	H13B—C13—H13C	109.5
C1—P1—S1	113.25 (9)	C2—C21—H21A	109.5
C13—C1—C12	108.6 (2)	C2—C21—H21B	109.5
C13—C1—C11	109.1 (3)	H21A—C21—H21B	109.5
C12—C1—C11	109.1 (2)	C2—C21—H21C	109.5
C13—C1—P1	110.25 (18)	H21A—C21—H21C	109.5
C12—C1—P1	110.19 (19)	H21B—C21—H21C	109.5
C11—C1—P1	109.59 (19)	C2—C22—H22A	109.5
C21—C2—C22	109.9 (2)	C2—C22—H22B	109.5
C21—C2—P1	113.0 (2)	H22A—C22—H22B	109.5
C22—C2—P1	112.4 (2)	C2—C22—H22C	109.5
C21—C2—H2	107.0	H22A—C22—H22C	109.5
C22—C2—H2	107.0	H22B—C22—H22C	109.5
P1—C2—H2	107.0	C3—C31—H31A	109.5
C32—C3—C31	111.2 (2)	C3—C31—H31B	109.5
C32—C3—P1	112.74 (18)	H31A—C31—H31B	109.5
C31—C3—P1	112.65 (18)	C3—C31—H31C	109.5
C32—C3—H3	106.6	H31A—C31—H31C	109.5
C31—C3—H3	106.6	H31B—C31—H31C	109.5
P1—C3—H3	106.6	C3—C32—H32A	109.5
C1—C11—H11A	109.5	C3—C32—H32B	109.5
C1—C11—H11B	109.5	H32A—C32—H32B	109.5
H11A—C11—H11B	109.5	C3—C32—H32C	109.5
C1—C11—H11C	109.5	H32A—C32—H32C	109.5
H11A—C11—H11C	109.5	H32B—C32—H32C	109.5
H11B—C11—H11C	109.5		
			
Cl3—Au1—S1—P1	56.17 (4)	S1—P1—C1—C11	150.83 (18)
Cl2—Au1—S1—P1	−122.80 (4)	C3—P1—C2—C21	−179.5 (2)
Au1—S1—P1—C2	165.31 (9)	C1—P1—C2—C21	59.7 (2)
Au1—S1—P1—C3	−77.45 (10)	S1—P1—C2—C21	−60.9 (2)
Au1—S1—P1—C1	47.12 (11)	C3—P1—C2—C22	−54.4 (2)
C2—P1—C1—C13	156.8 (2)	C1—P1—C2—C22	−175.1 (2)
C3—P1—C1—C13	36.3 (2)	S1—P1—C2—C22	64.2 (2)
S1—P1—C1—C13	−89.1 (2)	C2—P1—C3—C32	81.7 (2)
C2—P1—C1—C12	−83.3 (2)	C1—P1—C3—C32	−157.68 (19)
C3—P1—C1—C12	156.11 (19)	S1—P1—C3—C32	−31.3 (2)
S1—P1—C1—C12	30.7 (2)	C2—P1—C3—C31	−45.2 (2)
C2—P1—C1—C11	36.8 (2)	C1—P1—C3—C31	75.4 (2)
C3—P1—C1—C11	−83.8 (2)	S1—P1—C3—C31	−158.18 (16)

### (tert-Butyldipropan-2-ylphosphane sulfide-κS)trichloridogold(III) (10a) Hydrogen-bond geometry (Å, º)

**Table:**

*D*—H···*A*	*D*—H	H···*A*	*D*···*A*	*D*—H···*A*
C32—H32*B*···Cl3	0.98	2.75	3.452 (3)	130
C3—H3···Cl3	1.00	2.89	3.476 (3)	118
C13—H13*A*···Cl3	0.98	2.85	3.729 (3)	150
C13—H13*B*···Cl2^i^	0.98	2.77	3.704 (3)	160
C31—H31*A*···Cl1^ii^	0.98	2.87	3.372 (3)	113
C13—H13*C*···Cl2^iii^	0.98	2.91	3.878 (3)	170
C32—H32*A*···Cl3^iv^	0.98	2.91	3.800 (3)	152

Symmetry codes: (i) *x*−1, *y*, *z*; (ii) −*x*+1/2, *y*+1/2, −*z*+3/2; (iii) −*x*+1, −*y*+1, −*z*+2; (iv) −*x*, −*y*+1, −*z*+1.

### (tert-Butyldipropan-2-ylphosphane sulfide-κS)trichloridogold(III) (10aa) Crystal data

**Table:**

[AuCl_3_(C_10_H_23_PS)]	*F*(000) = 976
*M**_r_* = 509.63	*D*_x_ = 2.073 Mg m^−^^3^
Monoclinic, *P*2_1_/*n*	Mo *K*α radiation, λ = 0.71073 Å
*a* = 7.9363 (3) Å	Cell parameters from 14677 reflections
*b* = 14.4096 (4) Å	θ = 2.8–30.4°
*c* = 14.2851 (4) Å	µ = 9.70 mm^−^^1^
β = 91.774 (3)°	*T* = 100 K
*V* = 1632.85 (9) Å^3^	Block, red
*Z* = 4	0.3 × 0.2 × 0.2 mm

### (tert-Butyldipropan-2-ylphosphane sulfide-κS)trichloridogold(III) (10aa) Data collection

**Table:**

Oxford Diffraction Xcalibur, Eos diffractometer	4881 independent reflections
Radiation source: Enhance (Mo) X-ray Source	4296 reflections with *I* > 2σ(*I*)
Detector resolution: 16.1419 pixels mm^-1^	*R*_int_ = 0.046
ω scan	θ_max_ = 30.8°, θ_min_ = 2.8°
Absorption correction: multi-scan (CrysAlisPro; Rigaku OD, 2020)	*h* = −11→11
*T*_min_ = 0.159, *T*_max_ = 0.247	*k* = −20→19
42542 measured reflections	*l* = −20→20

### (tert-Butyldipropan-2-ylphosphane sulfide-κS)trichloridogold(III) (10aa) Refinement

**Table:**

Refinement on *F*^2^	Primary atom site location: structure-invariant direct methods
Least-squares matrix: full	Secondary atom site location: difference Fourier map
*R*[*F*^2^ > 2σ(*F*^2^)] = 0.025	Hydrogen site location: inferred from neighbouring sites
*wR*(*F*^2^) = 0.045	H-atom parameters constrained
*S* = 1.12	*w* = 1/[σ^2^(*F*_o_^2^) + (0.0115*P*)^2^ + 2.5557*P*] where *P* = (*F*_o_^2^ + 2*F*_c_^2^)/3
4881 reflections	(Δ/σ)_max_ = 0.001
152 parameters	Δρ_max_ = 2.52 e Å^−^^3^
0 restraints	Δρ_min_ = −1.50 e Å^−^^3^

### (tert-Butyldipropan-2-ylphosphane sulfide-κS)trichloridogold(III) (10aa) Fractional atomic coordinates and isotropic or equivalent isotropic displacement parameters (Å^2^)

**Table:**

	*x*	*y*	*z*	*U*_iso_*/*U*_eq_	
Au1	0.47137 (2)	0.07045 (2)	0.32157 (2)	0.01326 (4)	
Cl1	0.43370 (11)	0.01606 (6)	0.17013 (5)	0.02278 (17)	
Cl2	0.18806 (10)	0.09762 (6)	0.32608 (6)	0.02314 (17)	
Cl3	0.75081 (10)	0.03377 (6)	0.31862 (6)	0.02016 (16)	
S1	0.49165 (10)	0.12326 (5)	0.47552 (5)	0.01625 (16)	
P1	0.65482 (10)	0.23397 (5)	0.48821 (5)	0.01177 (15)	
C1	0.6113 (4)	0.3267 (2)	0.3991 (2)	0.0177 (7)	
C2	0.6125 (4)	0.2749 (2)	0.6072 (2)	0.0164 (6)	
H2	0.701534	0.321315	0.624880	0.020*	
C3	0.8755 (4)	0.1971 (2)	0.4808 (2)	0.0217 (7)	
H3	0.889522	0.177394	0.414348	0.026*	
C11	0.6778 (4)	0.4205 (2)	0.4368 (2)	0.0226 (7)	
H11A	0.664136	0.467885	0.387920	0.034*	
H11B	0.797448	0.414501	0.454791	0.034*	
H11C	0.614084	0.438648	0.491501	0.034*	
C12	0.4234 (5)	0.3349 (3)	0.3759 (3)	0.0324 (9)	
H12A	0.363374	0.349423	0.433018	0.049*	
H12B	0.381545	0.276083	0.349810	0.049*	
H12C	0.404201	0.384591	0.329903	0.049*	
C13	0.7034 (6)	0.3016 (2)	0.3089 (2)	0.0331 (9)	
H13A	0.669156	0.239351	0.288111	0.050*	
H13B	0.825429	0.302658	0.321490	0.050*	
H13C	0.673948	0.346738	0.259745	0.050*	
C21	0.4421 (5)	0.3221 (2)	0.6167 (2)	0.0247 (8)	
H21A	0.351900	0.277980	0.600347	0.037*	
H21B	0.434612	0.375598	0.574483	0.037*	
H21C	0.430237	0.342953	0.681460	0.037*	
C22	0.6245 (5)	0.1950 (3)	0.6794 (2)	0.0278 (8)	
H22A	0.612684	0.220219	0.742566	0.042*	
H22B	0.734042	0.164198	0.675259	0.042*	
H22C	0.534206	0.150064	0.666093	0.042*	
C31	1.0042 (5)	0.2758 (3)	0.4984 (4)	0.0467 (13)	
H31A	0.987941	0.302640	0.560531	0.070*	
H31B	0.987610	0.323988	0.450586	0.070*	
H31C	1.118712	0.250844	0.495347	0.070*	
C32	0.9210 (5)	0.1124 (3)	0.5405 (3)	0.0389 (11)	
H32A	1.025232	0.084618	0.518326	0.058*	
H32B	0.829555	0.066760	0.535428	0.058*	
H32C	0.937441	0.131286	0.606022	0.058*	

### (tert-Butyldipropan-2-ylphosphane sulfide-κS)trichloridogold(III) (10aa) Atomic displacement parameters (Å^2^)

**Table:**

	*U*^11^	*U*^22^	*U*^33^	*U*^12^	*U*^13^	*U*^23^
Au1	0.01406 (6)	0.01090 (6)	0.01491 (6)	−0.00074 (5)	0.00199 (4)	−0.00097 (4)
Cl1	0.0267 (4)	0.0249 (4)	0.0166 (4)	0.0004 (3)	−0.0017 (3)	−0.0040 (3)
Cl2	0.0141 (4)	0.0280 (4)	0.0274 (4)	0.0012 (3)	0.0008 (3)	−0.0010 (3)
Cl3	0.0158 (4)	0.0216 (4)	0.0232 (4)	0.0025 (3)	0.0025 (3)	−0.0077 (3)
S1	0.0198 (4)	0.0138 (4)	0.0154 (4)	−0.0050 (3)	0.0055 (3)	−0.0016 (3)
P1	0.0117 (4)	0.0104 (4)	0.0132 (4)	0.0005 (3)	0.0017 (3)	−0.0007 (3)
C1	0.0267 (17)	0.0126 (15)	0.0141 (15)	0.0007 (13)	0.0026 (13)	0.0024 (12)
C2	0.0171 (15)	0.0186 (16)	0.0136 (14)	0.0001 (12)	0.0031 (12)	−0.0049 (12)
C3	0.0162 (16)	0.0229 (18)	0.0261 (18)	0.0047 (13)	0.0000 (13)	−0.0079 (14)
C11	0.0268 (18)	0.0126 (16)	0.0287 (18)	−0.0035 (13)	0.0069 (14)	0.0010 (13)
C12	0.036 (2)	0.0202 (19)	0.040 (2)	0.0030 (16)	−0.0183 (18)	0.0052 (16)
C13	0.064 (3)	0.0171 (18)	0.0192 (17)	0.0021 (18)	0.0139 (18)	0.0036 (14)
C21	0.0299 (19)	0.0241 (18)	0.0205 (17)	0.0074 (15)	0.0094 (15)	0.0023 (14)
C22	0.036 (2)	0.033 (2)	0.0152 (16)	0.0104 (17)	0.0005 (15)	0.0009 (15)
C31	0.0168 (19)	0.046 (3)	0.077 (3)	−0.0011 (18)	−0.001 (2)	−0.037 (2)
C32	0.032 (2)	0.054 (3)	0.031 (2)	0.029 (2)	0.0032 (17)	0.0061 (19)

### (tert-Butyldipropan-2-ylphosphane sulfide-κS)trichloridogold(III) (10aa) Geometric parameters (Å, º)

**Table:**

Au1—Cl3	2.2815 (8)	C11—H11C	0.9800
Au1—Cl2	2.2851 (8)	C12—H12A	0.9800
Au1—Cl1	2.3116 (8)	C12—H12B	0.9800
Au1—S1	2.3281 (8)	C12—H12C	0.9800
S1—P1	2.0592 (11)	C13—H13A	0.9800
P1—C3	1.836 (3)	C13—H13B	0.9800
P1—C2	1.839 (3)	C13—H13C	0.9800
P1—C1	1.871 (3)	C21—H21A	0.9800
C1—C12	1.522 (5)	C21—H21B	0.9800
C1—C11	1.541 (4)	C21—H21C	0.9800
C1—C13	1.544 (4)	C22—H22A	0.9800
C2—C21	1.524 (4)	C22—H22B	0.9800
C2—C22	1.546 (5)	C22—H22C	0.9800
C2—H2	1.0000	C31—H31A	0.9800
C3—C32	1.527 (5)	C31—H31B	0.9800
C3—C31	1.542 (5)	C31—H31C	0.9800
C3—H3	1.0000	C32—H32A	0.9800
C11—H11A	0.9800	C32—H32B	0.9800
C11—H11B	0.9800	C32—H32C	0.9800
			
Cl3—Au1—Cl2	176.43 (3)	C1—C12—H12A	109.5
Cl3—Au1—Cl1	90.10 (3)	C1—C12—H12B	109.5
Cl2—Au1—Cl1	89.18 (3)	H12A—C12—H12B	109.5
Cl3—Au1—S1	93.11 (3)	C1—C12—H12C	109.5
Cl2—Au1—S1	87.51 (3)	H12A—C12—H12C	109.5
Cl1—Au1—S1	176.43 (3)	H12B—C12—H12C	109.5
P1—S1—Au1	111.18 (4)	C1—C13—H13A	109.5
C3—P1—C2	110.36 (15)	C1—C13—H13B	109.5
C3—P1—C1	108.88 (16)	H13A—C13—H13B	109.5
C2—P1—C1	111.39 (14)	C1—C13—H13C	109.5
C3—P1—S1	111.66 (12)	H13A—C13—H13C	109.5
C2—P1—S1	101.38 (11)	H13B—C13—H13C	109.5
C1—P1—S1	113.03 (11)	C2—C21—H21A	109.5
C12—C1—C11	109.3 (3)	C2—C21—H21B	109.5
C12—C1—C13	108.8 (3)	H21A—C21—H21B	109.5
C11—C1—C13	109.3 (3)	C2—C21—H21C	109.5
C12—C1—P1	111.3 (2)	H21A—C21—H21C	109.5
C11—C1—P1	109.5 (2)	H21B—C21—H21C	109.5
C13—C1—P1	108.6 (2)	C2—C22—H22A	109.5
C21—C2—C22	108.0 (3)	C2—C22—H22B	109.5
C21—C2—P1	114.4 (2)	H22A—C22—H22B	109.5
C22—C2—P1	111.6 (2)	C2—C22—H22C	109.5
C21—C2—H2	107.5	H22A—C22—H22C	109.5
C22—C2—H2	107.5	H22B—C22—H22C	109.5
P1—C2—H2	107.5	C3—C31—H31A	109.5
C32—C3—C31	110.7 (3)	C3—C31—H31B	109.5
C32—C3—P1	114.1 (3)	H31A—C31—H31B	109.5
C31—C3—P1	113.9 (2)	C3—C31—H31C	109.5
C32—C3—H3	105.8	H31A—C31—H31C	109.5
C31—C3—H3	105.8	H31B—C31—H31C	109.5
P1—C3—H3	105.8	C3—C32—H32A	109.5
C1—C11—H11A	109.5	C3—C32—H32B	109.5
C1—C11—H11B	109.5	H32A—C32—H32B	109.5
H11A—C11—H11B	109.5	C3—C32—H32C	109.5
C1—C11—H11C	109.5	H32A—C32—H32C	109.5
H11A—C11—H11C	109.5	H32B—C32—H32C	109.5
H11B—C11—H11C	109.5		
			
Cl3—Au1—S1—P1	61.27 (5)	S1—P1—C1—C13	−85.6 (3)
Cl2—Au1—S1—P1	−122.25 (5)	C3—P1—C2—C21	170.2 (2)
Au1—S1—P1—C3	−73.62 (13)	C1—P1—C2—C21	49.1 (3)
Au1—S1—P1—C2	168.88 (11)	S1—P1—C2—C21	−71.3 (2)
Au1—S1—P1—C1	49.55 (12)	C3—P1—C2—C22	−66.8 (3)
C3—P1—C1—C12	158.8 (2)	C1—P1—C2—C22	172.2 (2)
C2—P1—C1—C12	−79.2 (3)	S1—P1—C2—C22	51.7 (2)
S1—P1—C1—C12	34.1 (3)	C2—P1—C3—C32	65.5 (3)
C3—P1—C1—C11	−80.2 (2)	C1—P1—C3—C32	−172.0 (3)
C2—P1—C1—C11	41.7 (3)	S1—P1—C3—C32	−46.5 (3)
S1—P1—C1—C11	155.07 (19)	C2—P1—C3—C31	−63.0 (3)
C3—P1—C1—C13	39.1 (3)	C1—P1—C3—C31	59.6 (3)
C2—P1—C1—C13	161.0 (2)	S1—P1—C3—C31	−174.9 (3)

### (tert-Butyldipropan-2-ylphosphane sulfide-κS)trichloridogold(III) (10aa) Hydrogen-bond geometry (Å, º)

**Table:**

*D*—H···*A*	*D*—H	H···*A*	*D*···*A*	*D*—H···*A*
C3—H3···Cl3	1.00	2.70	3.427 (3)	130
C3—H3···Cl2^i^	1.00	2.95	3.665 (3)	129
C21—H21*C*···Cl2^ii^	0.98	2.99	3.706 (4)	131
C22—H22*A*···Cl2^ii^	0.98	2.94	3.675 (4)	133
C13—H13*C*···Cl3^iii^	0.98	2.99	3.832 (4)	145

Symmetry codes: (i) *x*+1, *y*, *z*; (ii) *x*+1/2, −*y*+1/2, *z*+1/2; (iii) −*x*+3/2, *y*+1/2, −*z*+1/2.

### Trichlorido[di-tert-butyl(propan-2-yl)phosphane sulfide-κS]gold(III) (11a) Crystal data

**Table:**

[AuCl_3_(C_11_H_25_PS)]	*Z* = 2
*M**_r_* = 523.66	*F*(000) = 504
Triclinic, *P*1	*D*_x_ = 2.011 Mg m^−^^3^
*a* = 8.6034 (4) Å	Mo *K*α radiation, λ = 0.71073 Å
*b* = 9.7779 (4) Å	Cell parameters from 31596 reflections
*c* = 11.4231 (4) Å	θ = 2.2–30.8°
α = 78.876 (3)°	µ = 9.16 mm^−^^1^
β = 71.456 (4)°	*T* = 100 K
γ = 72.702 (4)°	Plate, red
*V* = 864.69 (7) Å^3^	0.15 × 0.15 × 0.08 mm

### Trichlorido[di-tert-butyl(propan-2-yl)phosphane sulfide-κS]gold(III) (11a) Data collection

**Table:**

Oxford Diffraction Xcalibur, Eos diffractometer	5155 independent reflections
Radiation source: Enhance (Mo) X-ray Source	4939 reflections with *I* > 2σ(*I*)
Detector resolution: 16.1419 pixels mm^-1^	*R*_int_ = 0.038
ω scan	θ_max_ = 30.8°, θ_min_ = 2.6°
Absorption correction: multi-scan (CrysAlisPro; Rigaku OD, 2020)	*h* = −12→12
*T*_min_ = 0.577, *T*_max_ = 1.000	*k* = −14→14
68815 measured reflections	*l* = −16→16

### Trichlorido[di-tert-butyl(propan-2-yl)phosphane sulfide-κS]gold(III) (11a) Refinement

**Table:**

Refinement on *F*^2^	Secondary atom site location: difference Fourier map
Least-squares matrix: full	Hydrogen site location: inferred from neighbouring sites
*R*[*F*^2^ > 2σ(*F*^2^)] = 0.016	H-atom parameters constrained
*wR*(*F*^2^) = 0.036	*w* = 1/[σ^2^(*F*_o_^2^) + (0.0168*P*)^2^ + 0.7385*P*] where *P* = (*F*_o_^2^ + 2*F*_c_^2^)/3
*S* = 1.08	(Δ/σ)_max_ = 0.003
5155 reflections	Δρ_max_ = 1.64 e Å^−^^3^
163 parameters	Δρ_min_ = −0.93 e Å^−^^3^
0 restraints	Extinction correction: SHELXL-2019/3 (Sheldrick, 2015), *F*_c_^*^ = *kF*_c_[1 + 0.001 *F*_c_^2^λ^3^/sin(2θ)]^-1/4^
Primary atom site location: structure-invariant direct methods	Extinction coefficient: 0.00097 (14)

### Trichlorido[di-tert-butyl(propan-2-yl)phosphane sulfide-κS]gold(III) (11a) Fractional atomic coordinates and isotropic or equivalent isotropic displacement parameters (Å^2^)

**Table:**

	*x*	*y*	*z*	*U*_iso_*/*U*_eq_	
Au1	0.46476 (2)	0.35014 (2)	0.24072 (2)	0.01322 (3)	
Cl1	0.53637 (8)	0.12179 (5)	0.34127 (5)	0.02358 (11)	
Cl2	0.73242 (6)	0.37151 (5)	0.21222 (5)	0.02016 (10)	
Cl3	0.20542 (7)	0.31734 (5)	0.25815 (5)	0.02182 (10)	
S1	0.40624 (6)	0.58036 (5)	0.13465 (4)	0.01345 (9)	
P1	0.21575 (6)	0.72281 (5)	0.24712 (4)	0.00958 (8)	
C1	0.2568 (2)	0.7093 (2)	0.40129 (16)	0.0120 (3)	
C2	0.2277 (2)	0.8996 (2)	0.15054 (16)	0.0129 (3)	
C3	0.0119 (2)	0.6799 (2)	0.27290 (18)	0.0144 (4)	
H3	0.028473	0.577669	0.311603	0.017*	
C11	0.1566 (3)	0.8445 (2)	0.46903 (18)	0.0166 (4)	
H11A	0.175463	0.830339	0.551228	0.025*	
H11B	0.035482	0.860654	0.478365	0.025*	
H11C	0.195308	0.928404	0.420505	0.025*	
C12	0.4458 (2)	0.6884 (2)	0.38625 (18)	0.0160 (4)	
H12A	0.464884	0.674279	0.468368	0.024*	
H12B	0.481411	0.773860	0.338457	0.024*	
H12C	0.511838	0.603622	0.342337	0.024*	
C13	0.2023 (3)	0.5771 (2)	0.48235 (17)	0.0154 (4)	
H13A	0.261577	0.491274	0.438534	0.023*	
H13B	0.079670	0.592422	0.499118	0.023*	
H13C	0.230881	0.563523	0.560995	0.023*	
C21	0.2573 (3)	0.8834 (2)	0.01298 (18)	0.0187 (4)	
H21A	0.249579	0.978272	−0.035494	0.028*	
H21B	0.170971	0.841459	0.005595	0.028*	
H21C	0.370012	0.820130	−0.018603	0.028*	
C22	0.0615 (3)	1.0154 (2)	0.19359 (19)	0.0172 (4)	
H22A	0.073803	1.109141	0.148052	0.026*	
H22B	0.036261	1.020447	0.282805	0.026*	
H22C	−0.031281	0.990337	0.177297	0.026*	
C23	0.3768 (3)	0.9485 (2)	0.15964 (19)	0.0179 (4)	
H23A	0.481893	0.873138	0.135293	0.027*	
H23B	0.357471	0.966281	0.245317	0.027*	
H23C	0.386403	1.037364	0.104132	0.027*	
C31	−0.0281 (3)	0.6790 (2)	0.1513 (2)	0.0231 (4)	
H31A	−0.116373	0.627857	0.168074	0.035*	
H31B	0.074582	0.630193	0.092018	0.035*	
H31C	−0.067901	0.778349	0.116337	0.035*	
C32	−0.1431 (3)	0.7650 (2)	0.3650 (2)	0.0204 (4)	
H32A	−0.166224	0.867347	0.333210	0.031*	
H32B	−0.120054	0.753022	0.445560	0.031*	
H32C	−0.241804	0.728860	0.374972	0.031*	

### Trichlorido[di-tert-butyl(propan-2-yl)phosphane sulfide-κS]gold(III) (11a) Atomic displacement parameters (Å^2^)

**Table:**

	*U*^11^	*U*^22^	*U*^33^	*U*^12^	*U*^13^	*U*^23^
Au1	0.01600 (5)	0.01034 (4)	0.01333 (4)	0.00004 (3)	−0.00561 (3)	−0.00404 (2)
Cl1	0.0333 (3)	0.0125 (2)	0.0272 (2)	−0.00151 (19)	−0.0165 (2)	−0.00033 (18)
Cl2	0.0157 (2)	0.0205 (2)	0.0245 (2)	0.00003 (18)	−0.00744 (18)	−0.00704 (18)
Cl3	0.0195 (2)	0.0144 (2)	0.0338 (3)	−0.00423 (18)	−0.0088 (2)	−0.00533 (18)
S1	0.0157 (2)	0.0119 (2)	0.01053 (18)	−0.00038 (16)	−0.00253 (16)	−0.00305 (15)
P1	0.0100 (2)	0.00913 (19)	0.00972 (19)	−0.00157 (16)	−0.00349 (16)	−0.00143 (15)
C1	0.0121 (9)	0.0137 (8)	0.0104 (7)	−0.0006 (7)	−0.0049 (6)	−0.0026 (6)
C2	0.0159 (9)	0.0111 (8)	0.0114 (8)	−0.0040 (7)	−0.0039 (7)	0.0006 (6)
C3	0.0118 (9)	0.0122 (8)	0.0209 (9)	−0.0040 (7)	−0.0074 (7)	0.0008 (7)
C11	0.0174 (10)	0.0167 (9)	0.0146 (8)	0.0000 (7)	−0.0041 (7)	−0.0067 (7)
C12	0.0121 (9)	0.0194 (9)	0.0176 (9)	−0.0008 (7)	−0.0066 (7)	−0.0053 (7)
C13	0.0185 (10)	0.0145 (9)	0.0115 (8)	−0.0015 (7)	−0.0050 (7)	−0.0003 (6)
C21	0.0268 (11)	0.0170 (9)	0.0128 (8)	−0.0073 (8)	−0.0065 (8)	0.0017 (7)
C22	0.0186 (10)	0.0113 (8)	0.0205 (9)	−0.0012 (7)	−0.0074 (8)	0.0004 (7)
C23	0.0198 (10)	0.0167 (9)	0.0179 (9)	−0.0086 (8)	−0.0034 (7)	0.0000 (7)
C31	0.0244 (12)	0.0246 (11)	0.0282 (11)	−0.0105 (9)	−0.0169 (9)	0.0023 (8)
C32	0.0096 (9)	0.0199 (10)	0.0283 (10)	−0.0027 (7)	−0.0033 (8)	0.0007 (8)

### Trichlorido[di-tert-butyl(propan-2-yl)phosphane sulfide-κS]gold(III) (11a) Geometric parameters (Å, º)

**Table:**

Au1—Cl2	2.2881 (5)	C12—H12B	0.9800
Au1—Cl3	2.2889 (5)	C12—H12C	0.9800
Au1—Cl1	2.3080 (5)	C13—H13A	0.9800
Au1—S1	2.3346 (5)	C13—H13B	0.9800
S1—P1	2.0665 (6)	C13—H13C	0.9800
P1—C3	1.8442 (19)	C21—H21A	0.9800
P1—C2	1.8741 (18)	C21—H21B	0.9800
P1—C1	1.8765 (18)	C21—H21C	0.9800
C1—C12	1.534 (3)	C22—H22A	0.9800
C1—C13	1.540 (3)	C22—H22B	0.9800
C1—C11	1.543 (3)	C22—H22C	0.9800
C2—C23	1.535 (3)	C23—H23A	0.9800
C2—C22	1.541 (3)	C23—H23B	0.9800
C2—C21	1.542 (3)	C23—H23C	0.9800
C3—C31	1.535 (3)	C31—H31A	0.9800
C3—C32	1.537 (3)	C31—H31B	0.9800
C3—H3	1.0000	C31—H31C	0.9800
C11—H11A	0.9800	C32—H32A	0.9800
C11—H11B	0.9800	C32—H32B	0.9800
C11—H11C	0.9800	C32—H32C	0.9800
C12—H12A	0.9800		
			
Cl2—Au1—Cl3	175.769 (17)	C1—C12—H12C	109.5
Cl2—Au1—Cl1	89.44 (2)	H12A—C12—H12C	109.5
Cl3—Au1—Cl1	89.35 (2)	H12B—C12—H12C	109.5
Cl2—Au1—S1	87.963 (19)	C1—C13—H13A	109.5
Cl3—Au1—S1	93.175 (19)	C1—C13—H13B	109.5
Cl1—Au1—S1	177.237 (19)	H13A—C13—H13B	109.5
P1—S1—Au1	111.35 (2)	C1—C13—H13C	109.5
C3—P1—C2	112.66 (9)	H13A—C13—H13C	109.5
C3—P1—C1	108.88 (9)	H13B—C13—H13C	109.5
C2—P1—C1	113.61 (8)	C2—C21—H21A	109.5
C3—P1—S1	109.17 (7)	C2—C21—H21B	109.5
C2—P1—S1	101.48 (6)	H21A—C21—H21B	109.5
C1—P1—S1	110.81 (6)	C2—C21—H21C	109.5
C12—C1—C13	108.33 (16)	H21A—C21—H21C	109.5
C12—C1—C11	108.22 (16)	H21B—C21—H21C	109.5
C13—C1—C11	108.84 (15)	C2—C22—H22A	109.5
C12—C1—P1	111.17 (13)	C2—C22—H22B	109.5
C13—C1—P1	108.09 (13)	H22A—C22—H22B	109.5
C11—C1—P1	112.11 (13)	C2—C22—H22C	109.5
C23—C2—C22	109.55 (16)	H22A—C22—H22C	109.5
C23—C2—C21	107.67 (16)	H22B—C22—H22C	109.5
C22—C2—C21	108.83 (16)	C2—C23—H23A	109.5
C23—C2—P1	110.43 (13)	C2—C23—H23B	109.5
C22—C2—P1	110.44 (13)	H23A—C23—H23B	109.5
C21—C2—P1	109.85 (13)	C2—C23—H23C	109.5
C31—C3—C32	110.93 (17)	H23A—C23—H23C	109.5
C31—C3—P1	112.85 (14)	H23B—C23—H23C	109.5
C32—C3—P1	116.89 (14)	C3—C31—H31A	109.5
C31—C3—H3	105.0	C3—C31—H31B	109.5
C32—C3—H3	105.0	H31A—C31—H31B	109.5
P1—C3—H3	105.0	C3—C31—H31C	109.5
C1—C11—H11A	109.5	H31A—C31—H31C	109.5
C1—C11—H11B	109.5	H31B—C31—H31C	109.5
H11A—C11—H11B	109.5	C3—C32—H32A	109.5
C1—C11—H11C	109.5	C3—C32—H32B	109.5
H11A—C11—H11C	109.5	H32A—C32—H32B	109.5
H11B—C11—H11C	109.5	C3—C32—H32C	109.5
C1—C12—H12A	109.5	H32A—C32—H32C	109.5
C1—C12—H12B	109.5	H32B—C32—H32C	109.5
H12A—C12—H12B	109.5		
			
Cl2—Au1—S1—P1	−117.55 (3)	C1—P1—C2—C23	41.02 (16)
Cl3—Au1—S1—P1	66.53 (3)	S1—P1—C2—C23	−77.95 (13)
Au1—S1—P1—C3	−71.70 (7)	C3—P1—C2—C22	44.11 (16)
Au1—S1—P1—C2	169.16 (6)	C1—P1—C2—C22	−80.31 (15)
Au1—S1—P1—C1	48.21 (7)	S1—P1—C2—C22	160.72 (12)
C3—P1—C1—C12	159.11 (13)	C3—P1—C2—C21	−75.95 (16)
C2—P1—C1—C12	−74.46 (15)	C1—P1—C2—C21	159.63 (13)
S1—P1—C1—C12	39.02 (14)	S1—P1—C2—C21	40.66 (14)
C3—P1—C1—C13	40.34 (15)	C2—P1—C3—C31	56.54 (17)
C2—P1—C1—C13	166.77 (12)	C1—P1—C3—C31	−176.48 (14)
S1—P1—C1—C13	−79.75 (13)	S1—P1—C3—C31	−55.39 (15)
C3—P1—C1—C11	−79.62 (15)	C2—P1—C3—C32	−73.88 (16)
C2—P1—C1—C11	46.82 (16)	C1—P1—C3—C32	53.10 (16)
S1—P1—C1—C11	160.30 (12)	S1—P1—C3—C32	174.19 (13)
C3—P1—C2—C23	165.44 (13)		

### Trichlorido[di-tert-butyl(propan-2-yl)phosphane sulfide-κS]gold(III) (11a) Hydrogen-bond geometry (Å, º)

**Table:**

*D*—H···*A*	*D*—H	H···*A*	*D*···*A*	*D*—H···*A*
C13—H13*A*···Au1	0.98	2.71	3.6142 (19)	154
C12—H12*C*···S1	0.98	2.86	3.391 (2)	115
C3—H3···Cl3	1.00	2.62	3.451 (2)	140
C12—H12*C*···Cl2	0.98	2.81	3.788 (2)	174
C13—H13*C*···Cl2^i^	0.98	2.91	3.851 (2)	161

Symmetry code: (i) −*x*+1, −*y*+1, −*z*+1.

### Trichlorido[di-tert-butyl(propan-2-yl)phosphane sulfide-κS]gold(III) chloroform-d monosilvate (11aa) Crystal data

**Table:**

[AuCl_3_(C_11_H_25_PS)]·CDCl_3_	*Z* = 2
*M**_r_* = 644.03	*F*(000) = 620
Triclinic, *P*1	*D*_x_ = 2.031 Mg m^−^^3^
*a* = 9.6382 (4) Å	Mo *K*α radiation, λ = 0.71073 Å
*b* = 10.2787 (3) Å	Cell parameters from 35195 reflections
*c* = 11.8483 (5) Å	θ = 2.3–30.9°
α = 75.115 (3)°	µ = 7.91 mm^−^^1^
β = 68.875 (4)°	*T* = 101 K
γ = 89.728 (3)°	Plate, dichroic red / orange
*V* = 1053.13 (8) Å^3^	0.15 × 0.06 × 0.05 mm

### Trichlorido[di-tert-butyl(propan-2-yl)phosphane sulfide-κS]gold(III) chloroform-d monosilvate (11aa) Data collection

**Table:**

Oxford Diffraction Xcalibur, Eos diffractometer	6247 independent reflections
Radiation source: fine-focus sealed tube	5976 reflections with *I* > 2σ(*I*)
Detector resolution: 16.1419 pixels mm^-1^	*R*_int_ = 0.038
ω scan	θ_max_ = 30.9°, θ_min_ = 2.3°
Absorption correction: multi-scan (CrysAlisPro; Rigaku OD, 2020)	*h* = −13→13
*T*_min_ = 0.454, *T*_max_ = 1.000	*k* = −14→14
76947 measured reflections	*l* = −17→17

### Trichlorido[di-tert-butyl(propan-2-yl)phosphane sulfide-κS]gold(III) chloroform-d monosilvate (11aa) Refinement

**Table:**

Refinement on *F*^2^	Secondary atom site location: difference Fourier map
Least-squares matrix: full	Hydrogen site location: inferred from neighbouring sites
*R*[*F*^2^ > 2σ(*F*^2^)] = 0.016	H-atom parameters constrained
*wR*(*F*^2^) = 0.033	*w* = 1/[σ^2^(*F*_o_^2^) + (0.0135*P*)^2^ + 0.8433*P*] where *P* = (*F*_o_^2^ + 2*F*_c_^2^)/3
*S* = 1.05	(Δ/σ)_max_ = 0.002
6247 reflections	Δρ_max_ = 1.02 e Å^−^^3^
199 parameters	Δρ_min_ = −0.95 e Å^−^^3^
0 restraints	Extinction correction: SHELXL-2019/3 (Sheldrick, 2015), *F*_c_^*^ = *kF*_c_[1 + 0.001 *F*_c_^2^λ^3^/sin(2θ)]^-1/4^
Primary atom site location: structure-invariant direct methods	Extinction coefficient: 0.00133 (9)

### Trichlorido[di-tert-butyl(propan-2-yl)phosphane sulfide-κS]gold(III) chloroform-d monosilvate (11aa) Fractional atomic coordinates and isotropic or equivalent isotropic displacement parameters (Å^2^)

**Table:**

	*x*	*y*	*z*	*U*_iso_*/*U*_eq_	
Au1	0.71266 (2)	0.36753 (2)	0.29738 (2)	0.01055 (3)	
Cl1	0.85075 (5)	0.38930 (5)	0.41619 (4)	0.01711 (9)	
Cl2	0.62356 (5)	0.57177 (5)	0.31290 (5)	0.01741 (9)	
Cl3	0.81385 (5)	0.16897 (5)	0.27791 (4)	0.01599 (9)	
S1	0.58436 (5)	0.35877 (5)	0.16572 (4)	0.01218 (8)	
P1	0.41142 (5)	0.20633 (5)	0.24806 (4)	0.00919 (8)	
C1	0.28613 (19)	0.21927 (19)	0.40702 (17)	0.0122 (3)	
C2	0.3218 (2)	0.23846 (19)	0.12786 (17)	0.0124 (3)	
C3	0.4907 (2)	0.04179 (18)	0.26592 (18)	0.0130 (3)	
H3	0.553904	0.045302	0.316195	0.016*	
C11	0.1340 (2)	0.1352 (2)	0.45315 (18)	0.0163 (4)	
H11A	0.080315	0.173434	0.397891	0.024*	
H11B	0.074531	0.137380	0.539288	0.024*	
H11C	0.150519	0.041430	0.451538	0.024*	
C12	0.2587 (2)	0.3669 (2)	0.40576 (19)	0.0171 (4)	
H12A	0.203030	0.371837	0.491747	0.026*	
H12B	0.200708	0.400980	0.353136	0.026*	
H12C	0.354755	0.422152	0.371587	0.026*	
C13	0.3657 (2)	0.1650 (2)	0.49907 (18)	0.0168 (4)	
H13A	0.304536	0.174026	0.582547	0.025*	
H13B	0.463110	0.216998	0.468932	0.025*	
H13C	0.379886	0.069480	0.504286	0.025*	
C21	0.4419 (2)	0.2753 (2)	−0.00570 (18)	0.0168 (4)	
H21A	0.498151	0.361608	−0.021724	0.025*	
H21B	0.393993	0.283422	−0.067086	0.025*	
H21C	0.510291	0.204345	−0.013415	0.025*	
C22	0.2238 (2)	0.1106 (2)	0.14607 (19)	0.0164 (4)	
H22A	0.172467	0.130120	0.086540	0.025*	
H22B	0.149695	0.083415	0.232245	0.025*	
H22C	0.287056	0.037131	0.130972	0.025*	
C23	0.2252 (2)	0.3569 (2)	0.13874 (19)	0.0175 (4)	
H23A	0.191371	0.380526	0.067932	0.026*	
H23B	0.284111	0.435102	0.137323	0.026*	
H23C	0.138221	0.330673	0.217906	0.026*	
C31	0.5994 (2)	0.0242 (2)	0.1402 (2)	0.0194 (4)	
H31A	0.542761	0.004996	0.091357	0.029*	
H31B	0.658640	−0.051177	0.156429	0.029*	
H31C	0.666269	0.107416	0.092779	0.029*	
C32	0.3807 (2)	−0.0861 (2)	0.3403 (2)	0.0188 (4)	
H32A	0.435800	−0.166423	0.339373	0.028*	
H32B	0.307285	−0.090300	0.301368	0.028*	
H32C	0.329015	−0.082875	0.427331	0.028*	
C99	0.9953 (2)	0.7075 (2)	0.19499 (19)	0.0174 (4)	
D99	0.908901	0.650866	0.267327	0.021*	
Cl4	1.15235 (7)	0.69803 (6)	0.23818 (6)	0.03287 (13)	
Cl5	1.02700 (7)	0.64377 (7)	0.06444 (5)	0.03256 (13)	
Cl6	0.94906 (6)	0.87534 (5)	0.16252 (6)	0.02991 (12)	

### Trichlorido[di-tert-butyl(propan-2-yl)phosphane sulfide-κS]gold(III) chloroform-d monosilvate (11aa) Atomic displacement parameters (Å^2^)

**Table:**

	*U*^11^	*U*^22^	*U*^33^	*U*^12^	*U*^13^	*U*^23^
Au1	0.00947 (3)	0.00984 (4)	0.01275 (4)	−0.00047 (2)	−0.00505 (2)	−0.00240 (2)
Cl1	0.0184 (2)	0.0171 (2)	0.0213 (2)	0.00185 (17)	−0.01281 (18)	−0.00659 (18)
Cl2	0.0179 (2)	0.0133 (2)	0.0260 (2)	0.00321 (16)	−0.01179 (18)	−0.00836 (18)
Cl3	0.01472 (19)	0.0135 (2)	0.0228 (2)	0.00333 (16)	−0.00933 (17)	−0.00660 (17)
S1	0.01193 (18)	0.0116 (2)	0.01239 (19)	−0.00215 (15)	−0.00557 (16)	−0.00065 (16)
P1	0.00947 (18)	0.0086 (2)	0.0102 (2)	0.00033 (15)	−0.00460 (16)	−0.00252 (16)
C1	0.0111 (7)	0.0147 (9)	0.0112 (8)	0.0001 (6)	−0.0038 (6)	−0.0045 (7)
C2	0.0139 (8)	0.0125 (9)	0.0126 (8)	0.0009 (6)	−0.0074 (7)	−0.0029 (7)
C3	0.0146 (8)	0.0104 (8)	0.0168 (9)	0.0029 (6)	−0.0089 (7)	−0.0037 (7)
C11	0.0116 (8)	0.0188 (10)	0.0167 (9)	−0.0022 (7)	−0.0029 (7)	−0.0049 (7)
C12	0.0163 (8)	0.0169 (10)	0.0192 (9)	0.0021 (7)	−0.0051 (7)	−0.0093 (8)
C13	0.0170 (9)	0.0215 (10)	0.0116 (8)	0.0005 (7)	−0.0059 (7)	−0.0031 (7)
C21	0.0206 (9)	0.0180 (10)	0.0128 (8)	0.0012 (7)	−0.0079 (7)	−0.0034 (7)
C22	0.0183 (9)	0.0157 (9)	0.0192 (9)	−0.0001 (7)	−0.0111 (7)	−0.0055 (7)
C23	0.0179 (9)	0.0155 (9)	0.0229 (10)	0.0046 (7)	−0.0122 (8)	−0.0049 (8)
C31	0.0216 (9)	0.0184 (10)	0.0230 (10)	0.0086 (8)	−0.0102 (8)	−0.0112 (8)
C32	0.0227 (9)	0.0109 (9)	0.0251 (10)	−0.0003 (7)	−0.0134 (8)	−0.0025 (8)
C99	0.0163 (8)	0.0164 (10)	0.0190 (9)	0.0002 (7)	−0.0071 (7)	−0.0034 (8)
Cl4	0.0319 (3)	0.0245 (3)	0.0538 (4)	0.0034 (2)	−0.0308 (3)	−0.0084 (3)
Cl5	0.0323 (3)	0.0471 (4)	0.0242 (3)	0.0140 (3)	−0.0109 (2)	−0.0191 (3)
Cl6	0.0246 (2)	0.0168 (2)	0.0473 (3)	0.0038 (2)	−0.0153 (2)	−0.0042 (2)

### Trichlorido[di-tert-butyl(propan-2-yl)phosphane sulfide-κS]gold(III) chloroform-d monosilvate (11aa) Geometric parameters (Å, º)

**Table:**

Au1—Cl3	2.2871 (5)	C13—H13A	0.9800
Au1—Cl2	2.2903 (5)	C13—H13B	0.9800
Au1—Cl1	2.3060 (4)	C13—H13C	0.9800
Au1—S1	2.3312 (4)	C21—H21A	0.9800
S1—P1	2.0622 (6)	C21—H21B	0.9800
P1—C3	1.8465 (19)	C21—H21C	0.9800
P1—C1	1.8737 (18)	C22—H22A	0.9800
P1—C2	1.8778 (18)	C22—H22B	0.9800
C1—C12	1.537 (3)	C22—H22C	0.9800
C1—C13	1.541 (2)	C23—H23A	0.9800
C1—C11	1.545 (2)	C23—H23B	0.9800
C2—C23	1.532 (3)	C23—H23C	0.9800
C2—C21	1.538 (3)	C31—H31A	0.9800
C2—C22	1.544 (3)	C31—H31B	0.9800
C3—C31	1.536 (3)	C31—H31C	0.9800
C3—C32	1.540 (3)	C32—H32A	0.9800
C3—H3	1.0000	C32—H32B	0.9800
C11—H11A	0.9800	C32—H32C	0.9800
C11—H11B	0.9800	C99—Cl5	1.758 (2)
C11—H11C	0.9800	C99—Cl4	1.759 (2)
C12—H12A	0.9800	C99—Cl6	1.761 (2)
C12—H12B	0.9800	C99—D99	1.0000
C12—H12C	0.9800		
			
Cl3—Au1—Cl2	177.050 (16)	C1—C13—H13A	109.5
Cl3—Au1—Cl1	88.505 (17)	C1—C13—H13B	109.5
Cl2—Au1—Cl1	89.783 (17)	H13A—C13—H13B	109.5
Cl3—Au1—S1	92.936 (17)	C1—C13—H13C	109.5
Cl2—Au1—S1	88.609 (16)	H13A—C13—H13C	109.5
Cl1—Au1—S1	175.816 (16)	H13B—C13—H13C	109.5
P1—S1—Au1	111.96 (2)	C2—C21—H21A	109.5
C3—P1—C1	108.94 (8)	C2—C21—H21B	109.5
C3—P1—C2	112.62 (8)	H21A—C21—H21B	109.5
C1—P1—C2	114.39 (8)	C2—C21—H21C	109.5
C3—P1—S1	108.73 (6)	H21A—C21—H21C	109.5
C1—P1—S1	110.75 (6)	H21B—C21—H21C	109.5
C2—P1—S1	101.09 (6)	C2—C22—H22A	109.5
C12—C1—C13	108.22 (15)	C2—C22—H22B	109.5
C12—C1—C11	108.90 (15)	H22A—C22—H22B	109.5
C13—C1—C11	109.41 (15)	C2—C22—H22C	109.5
C12—C1—P1	111.28 (13)	H22A—C22—H22C	109.5
C13—C1—P1	107.56 (12)	H22B—C22—H22C	109.5
C11—C1—P1	111.40 (12)	C2—C23—H23A	109.5
C23—C2—C21	107.64 (15)	C2—C23—H23B	109.5
C23—C2—C22	109.35 (15)	H23A—C23—H23B	109.5
C21—C2—C22	108.61 (15)	C2—C23—H23C	109.5
C23—C2—P1	110.76 (13)	H23A—C23—H23C	109.5
C21—C2—P1	110.35 (12)	H23B—C23—H23C	109.5
C22—C2—P1	110.07 (12)	C3—C31—H31A	109.5
C31—C3—C32	110.45 (16)	C3—C31—H31B	109.5
C31—C3—P1	112.59 (13)	H31A—C31—H31B	109.5
C32—C3—P1	117.44 (13)	C3—C31—H31C	109.5
C31—C3—H3	105.0	H31A—C31—H31C	109.5
C32—C3—H3	105.0	H31B—C31—H31C	109.5
P1—C3—H3	105.0	C3—C32—H32A	109.5
C1—C11—H11A	109.5	C3—C32—H32B	109.5
C1—C11—H11B	109.5	H32A—C32—H32B	109.5
H11A—C11—H11B	109.5	C3—C32—H32C	109.5
C1—C11—H11C	109.5	H32A—C32—H32C	109.5
H11A—C11—H11C	109.5	H32B—C32—H32C	109.5
H11B—C11—H11C	109.5	Cl5—C99—Cl4	110.93 (11)
C1—C12—H12A	109.5	Cl5—C99—Cl6	110.46 (11)
C1—C12—H12B	109.5	Cl4—C99—Cl6	110.43 (11)
H12A—C12—H12B	109.5	Cl5—C99—D99	108.3
C1—C12—H12C	109.5	Cl4—C99—D99	108.3
H12A—C12—H12C	109.5	Cl6—C99—D99	108.3
H12B—C12—H12C	109.5		
			
Cl3—Au1—S1—P1	69.79 (3)	C1—P1—C2—C23	40.95 (16)
Cl2—Au1—S1—P1	−112.73 (3)	S1—P1—C2—C23	−78.08 (13)
Au1—S1—P1—C3	−66.93 (7)	C3—P1—C2—C21	−74.85 (15)
Au1—S1—P1—C1	52.74 (7)	C1—P1—C2—C21	160.04 (13)
Au1—S1—P1—C2	174.37 (6)	S1—P1—C2—C21	41.01 (14)
C3—P1—C1—C12	161.09 (12)	C3—P1—C2—C22	45.01 (15)
C2—P1—C1—C12	−71.89 (14)	C1—P1—C2—C22	−80.10 (15)
S1—P1—C1—C12	41.54 (13)	S1—P1—C2—C22	160.87 (12)
C3—P1—C1—C13	42.71 (14)	C1—P1—C3—C31	−178.11 (13)
C2—P1—C1—C13	169.73 (12)	C2—P1—C3—C31	53.87 (15)
S1—P1—C1—C13	−76.84 (13)	S1—P1—C3—C31	−57.31 (14)
C3—P1—C1—C11	−77.18 (14)	C1—P1—C3—C32	51.93 (16)
C2—P1—C1—C11	49.84 (16)	C2—P1—C3—C32	−76.10 (16)
S1—P1—C1—C11	163.27 (11)	S1—P1—C3—C32	172.72 (12)
C3—P1—C2—C23	166.06 (13)		

### Trichlorido[di-tert-butyl(propan-2-yl)phosphane sulfide-κS]gold(III) chloroform-d monosilvate (11aa) Hydrogen-bond geometry (Å, º)

**Table:**

*D*—H···*A*	*D*—H	H···*A*	*D*···*A*	*D*—H···*A*
C13—H13*B*···Au1	0.98	2.68	3.6027 (19)	156
C21—H21*A*···S1	0.98	2.63	3.1082 (19)	110
C12—H12*C*···S1	0.98	2.86	3.411 (2)	116
C3—H3···Cl3	1.00	2.65	3.4471 (18)	137
C12—H12*C*···Cl2	0.98	2.78	3.755 (2)	171
C99—D99···Cl1	1.00	2.74	3.537 (2)	137
C99—D99···Cl2	1.00	2.69	3.489 (2)	137
C12—H12*A*···Cl1^i^	0.98	2.91	3.596 (2)	128

Symmetry code: (i) −*x*+1, −*y*+1, −*z*+1.

### Trichlorido(tri-tert-butylphosphane sulfide-κS)gold(III) dichloromethane monosolvate (12a) Crystal data

**Table:**

[AuCl_3_(C_12_H_27_PS)]·CH_2_Cl_2_	*Z* = 2
*M**_r_* = 622.61	*F*(000) = 604
Triclinic, *P*1	*D*_x_ = 1.924 Mg m^−^^3^
*a* = 8.4202 (3) Å	Mo *K*α radiation, λ = 0.71073 Å
*b* = 11.2194 (4) Å	Cell parameters from 47731 reflections
*c* = 11.8355 (4) Å	θ = 2.8–30.6°
α = 98.398 (3)°	µ = 7.63 mm^−^^1^
β = 101.174 (3)°	*T* = 100 K
γ = 95.991 (3)°	Block, red
*V* = 1074.95 (7) Å^3^	0.15 × 0.1 × 0.1 mm

### Trichlorido(tri-tert-butylphosphane sulfide-κS)gold(III) dichloromethane monosolvate (12a) Data collection

**Table:**

Oxford Diffraction Xcalibur, Eos diffractometer	6558 independent reflections
Radiation source: Enhance (Mo) X-ray Source	6124 reflections with *I* > 2σ(*I*)
Detector resolution: 16.1419 pixels mm^-1^	*R*_int_ = 0.054
ω–scan	θ_max_ = 31.1°, θ_min_ = 2.3°
Absorption correction: multi-scan (CrysAlisPro; Rigaku OD, 2020)	*h* = −12→12
*T*_min_ = 0.783, *T*_max_ = 1.000	*k* = −16→16
157605 measured reflections	*l* = −16→16

### Trichlorido(tri-tert-butylphosphane sulfide-κS)gold(III) dichloromethane monosolvate (12a) Refinement

**Table:**

Refinement on *F*^2^	Primary atom site location: structure-invariant direct methods
Least-squares matrix: full	Secondary atom site location: difference Fourier map
*R*[*F*^2^ > 2σ(*F*^2^)] = 0.021	Hydrogen site location: inferred from neighbouring sites
*wR*(*F*^2^) = 0.037	H-atom parameters constrained
*S* = 1.05	*w* = 1/[σ^2^(*F*_o_^2^) + (0.0147*P*)^2^ + 0.9642*P*] where *P* = (*F*_o_^2^ + 2*F*_c_^2^)/3
6558 reflections	(Δ/σ)_max_ = 0.002
199 parameters	Δρ_max_ = 1.76 e Å^−^^3^
0 restraints	Δρ_min_ = −1.01 e Å^−^^3^

### Trichlorido(tri-tert-butylphosphane sulfide-κS)gold(III) dichloromethane monosolvate (12a) Fractional atomic coordinates and isotropic or equivalent isotropic displacement parameters (Å^2^)

**Table:**

	*x*	*y*	*z*	*U*_iso_*/*U*_eq_	
Au1	0.53561 (2)	0.65489 (2)	0.29756 (2)	0.01423 (3)	
P1	0.63223 (6)	0.33330 (4)	0.26499 (4)	0.01110 (9)	
S1	0.55574 (6)	0.47197 (4)	0.36749 (4)	0.01588 (10)	
Cl1	0.51329 (8)	0.84355 (5)	0.24639 (6)	0.03068 (13)	
Cl2	0.78232 (7)	0.73160 (5)	0.41951 (5)	0.02751 (12)	
Cl3	0.28358 (6)	0.58666 (4)	0.18025 (5)	0.01819 (10)	
C1	0.6853 (2)	0.23096 (17)	0.37810 (18)	0.0147 (4)	
C2	0.8182 (2)	0.38937 (18)	0.21024 (18)	0.0156 (4)	
C3	0.4579 (2)	0.25575 (17)	0.14205 (18)	0.0152 (4)	
C11	0.7890 (3)	0.13553 (18)	0.3356 (2)	0.0189 (4)	
H11A	0.896288	0.176237	0.332015	0.028*	
H11B	0.733736	0.091959	0.257732	0.028*	
H11C	0.803183	0.077662	0.390133	0.028*	
C12	0.5282 (3)	0.16507 (19)	0.4010 (2)	0.0187 (4)	
H12A	0.556046	0.121650	0.466725	0.028*	
H12B	0.472365	0.106869	0.331072	0.028*	
H12C	0.456061	0.224697	0.419971	0.028*	
C13	0.7792 (3)	0.3055 (2)	0.49633 (19)	0.0204 (4)	
H13A	0.806888	0.250522	0.552104	0.031*	
H13B	0.710742	0.362704	0.526409	0.031*	
H13C	0.879701	0.350796	0.485668	0.031*	
C21	0.8590 (3)	0.2910 (2)	0.1190 (2)	0.0210 (4)	
H21A	0.959839	0.320758	0.096282	0.031*	
H21B	0.769214	0.272475	0.049961	0.031*	
H21C	0.873861	0.217124	0.152734	0.031*	
C22	0.9645 (3)	0.4278 (2)	0.3140 (2)	0.0217 (4)	
H22A	1.055508	0.469794	0.288068	0.033*	
H22B	0.998283	0.355582	0.344279	0.033*	
H22C	0.933090	0.482692	0.375708	0.033*	
C23	0.7922 (3)	0.50296 (19)	0.1537 (2)	0.0198 (4)	
H23A	0.785061	0.571139	0.213648	0.030*	
H23B	0.690694	0.486182	0.093839	0.030*	
H23C	0.884367	0.523857	0.117485	0.030*	
C31	0.4816 (3)	0.12534 (18)	0.0939 (2)	0.0209 (4)	
H31A	0.392796	0.091382	0.026551	0.031*	
H31B	0.480460	0.074977	0.154879	0.031*	
H31C	0.586512	0.126580	0.069678	0.031*	
C32	0.2951 (2)	0.25318 (19)	0.1836 (2)	0.0186 (4)	
H32A	0.277011	0.336554	0.210355	0.028*	
H32B	0.299746	0.207114	0.248201	0.028*	
H32C	0.205260	0.214234	0.118818	0.028*	
C33	0.4420 (3)	0.32898 (19)	0.04080 (19)	0.0191 (4)	
H33A	0.538213	0.325449	0.005922	0.029*	
H33B	0.434062	0.413848	0.070728	0.029*	
H33C	0.343501	0.294182	−0.018603	0.029*	
C99	1.0588 (4)	0.8679 (2)	0.2453 (2)	0.0322 (6)	
H99A	0.963855	0.820052	0.262990	0.039*	
H99B	1.141490	0.813027	0.234783	0.039*	
Cl4	1.14230 (7)	0.98659 (5)	0.36326 (5)	0.02400 (11)	
Cl5	0.99590 (10)	0.92468 (7)	0.11422 (6)	0.04378 (17)	

### Trichlorido(tri-tert-butylphosphane sulfide-κS)gold(III) dichloromethane monosolvate (12a) Atomic displacement parameters (Å^2^)

**Table:**

	*U*^11^	*U*^22^	*U*^33^	*U*^12^	*U*^13^	*U*^23^
Au1	0.01635 (4)	0.01008 (3)	0.01700 (5)	0.00177 (2)	0.00743 (3)	−0.00052 (2)
P1	0.0114 (2)	0.0111 (2)	0.0110 (2)	0.00285 (16)	0.00292 (18)	0.00101 (17)
S1	0.0220 (2)	0.0139 (2)	0.0133 (2)	0.00565 (17)	0.00704 (19)	0.00088 (17)
Cl1	0.0464 (3)	0.0118 (2)	0.0372 (3)	0.0048 (2)	0.0158 (3)	0.0055 (2)
Cl2	0.0202 (2)	0.0244 (3)	0.0320 (3)	−0.00319 (19)	0.0049 (2)	−0.0094 (2)
Cl3	0.0174 (2)	0.0178 (2)	0.0199 (3)	0.00508 (17)	0.00395 (19)	0.00340 (18)
C1	0.0158 (9)	0.0145 (8)	0.0148 (10)	0.0040 (7)	0.0026 (8)	0.0052 (7)
C2	0.0149 (9)	0.0168 (9)	0.0174 (11)	0.0031 (7)	0.0079 (8)	0.0041 (7)
C3	0.0148 (9)	0.0131 (8)	0.0157 (10)	0.0031 (7)	0.0012 (8)	−0.0016 (7)
C11	0.0204 (10)	0.0179 (9)	0.0206 (11)	0.0084 (8)	0.0043 (8)	0.0063 (8)
C12	0.0190 (9)	0.0193 (9)	0.0197 (11)	0.0018 (7)	0.0063 (8)	0.0071 (8)
C13	0.0225 (10)	0.0235 (10)	0.0150 (11)	0.0048 (8)	0.0008 (9)	0.0054 (8)
C21	0.0214 (10)	0.0226 (10)	0.0229 (12)	0.0087 (8)	0.0117 (9)	0.0034 (8)
C22	0.0152 (9)	0.0253 (10)	0.0239 (12)	−0.0011 (8)	0.0044 (9)	0.0047 (9)
C23	0.0231 (10)	0.0186 (9)	0.0212 (12)	0.0036 (8)	0.0109 (9)	0.0058 (8)
C31	0.0230 (10)	0.0152 (9)	0.0208 (12)	0.0048 (8)	−0.0004 (9)	−0.0037 (8)
C32	0.0114 (8)	0.0190 (9)	0.0237 (12)	0.0019 (7)	0.0012 (8)	0.0013 (8)
C33	0.0217 (10)	0.0204 (10)	0.0144 (11)	0.0069 (8)	0.0011 (8)	0.0010 (8)
C99	0.0485 (15)	0.0211 (11)	0.0241 (13)	0.0000 (10)	0.0032 (12)	0.0043 (9)
Cl4	0.0240 (2)	0.0242 (2)	0.0232 (3)	0.00542 (19)	0.0015 (2)	0.0053 (2)
Cl5	0.0588 (5)	0.0443 (4)	0.0230 (3)	0.0033 (3)	−0.0035 (3)	0.0075 (3)

### Trichlorido(tri-tert-butylphosphane sulfide-κS)gold(III) dichloromethane monosolvate (12a) Geometric parameters (Å, º)

**Table:**

Au1—Cl3	2.2860 (5)	C13—H13B	0.9800
Au1—Cl2	2.2894 (6)	C13—H13C	0.9800
Au1—Cl1	2.3013 (5)	C21—H21A	0.9800
Au1—S1	2.3323 (5)	C21—H21B	0.9800
P1—C3	1.888 (2)	C21—H21C	0.9800
P1—C2	1.8906 (19)	C22—H22A	0.9800
P1—C1	1.906 (2)	C22—H22B	0.9800
P1—S1	2.0658 (6)	C22—H22C	0.9800
C1—C12	1.537 (3)	C23—H23A	0.9800
C1—C11	1.540 (3)	C23—H23B	0.9800
C1—C13	1.541 (3)	C23—H23C	0.9800
C2—C22	1.536 (3)	C31—H31A	0.9800
C2—C23	1.540 (3)	C31—H31B	0.9800
C2—C21	1.542 (3)	C31—H31C	0.9800
C3—C31	1.538 (3)	C32—H32A	0.9800
C3—C33	1.542 (3)	C32—H32B	0.9800
C3—C32	1.542 (3)	C32—H32C	0.9800
C11—H11A	0.9800	C33—H33A	0.9800
C11—H11B	0.9800	C33—H33B	0.9800
C11—H11C	0.9800	C33—H33C	0.9800
C12—H12A	0.9800	C99—Cl4	1.760 (3)
C12—H12B	0.9800	C99—Cl5	1.770 (3)
C12—H12C	0.9800	C99—H99A	0.9900
C13—H13A	0.9800	C99—H99B	0.9900
			
Cl3—Au1—Cl2	176.996 (18)	C1—C13—H13C	109.5
Cl3—Au1—Cl1	88.53 (2)	H13A—C13—H13C	109.5
Cl2—Au1—Cl1	89.55 (2)	H13B—C13—H13C	109.5
Cl3—Au1—S1	93.402 (18)	C2—C21—H21A	109.5
Cl2—Au1—S1	88.30 (2)	C2—C21—H21B	109.5
Cl1—Au1—S1	174.441 (19)	H21A—C21—H21B	109.5
C3—P1—C2	112.33 (9)	C2—C21—H21C	109.5
C3—P1—C1	111.29 (9)	H21A—C21—H21C	109.5
C2—P1—C1	111.38 (9)	H21B—C21—H21C	109.5
C3—P1—S1	110.17 (6)	C2—C22—H22A	109.5
C2—P1—S1	111.66 (6)	C2—C22—H22B	109.5
C1—P1—S1	99.33 (6)	H22A—C22—H22B	109.5
P1—S1—Au1	117.50 (3)	C2—C22—H22C	109.5
C12—C1—C11	108.93 (16)	H22A—C22—H22C	109.5
C12—C1—C13	106.51 (17)	H22B—C22—H22C	109.5
C11—C1—C13	109.07 (17)	C2—C23—H23A	109.5
C12—C1—P1	110.11 (14)	C2—C23—H23B	109.5
C11—C1—P1	110.82 (14)	H23A—C23—H23B	109.5
C13—C1—P1	111.28 (13)	C2—C23—H23C	109.5
C22—C2—C23	106.45 (17)	H23A—C23—H23C	109.5
C22—C2—C21	110.01 (17)	H23B—C23—H23C	109.5
C23—C2—C21	108.11 (17)	C3—C31—H31A	109.5
C22—C2—P1	109.39 (14)	C3—C31—H31B	109.5
C23—C2—P1	111.29 (13)	H31A—C31—H31B	109.5
C21—C2—P1	111.46 (14)	C3—C31—H31C	109.5
C31—C3—C33	108.18 (17)	H31A—C31—H31C	109.5
C31—C3—C32	109.39 (16)	H31B—C31—H31C	109.5
C33—C3—C32	106.69 (17)	C3—C32—H32A	109.5
C31—C3—P1	112.55 (14)	C3—C32—H32B	109.5
C33—C3—P1	109.73 (13)	H32A—C32—H32B	109.5
C32—C3—P1	110.12 (14)	C3—C32—H32C	109.5
C1—C11—H11A	109.5	H32A—C32—H32C	109.5
C1—C11—H11B	109.5	H32B—C32—H32C	109.5
H11A—C11—H11B	109.5	C3—C33—H33A	109.5
C1—C11—H11C	109.5	C3—C33—H33B	109.5
H11A—C11—H11C	109.5	H33A—C33—H33B	109.5
H11B—C11—H11C	109.5	C3—C33—H33C	109.5
C1—C12—H12A	109.5	H33A—C33—H33C	109.5
C1—C12—H12B	109.5	H33B—C33—H33C	109.5
H12A—C12—H12B	109.5	Cl4—C99—Cl5	111.42 (13)
C1—C12—H12C	109.5	Cl4—C99—H99A	109.3
H12A—C12—H12C	109.5	Cl5—C99—H99A	109.3
H12B—C12—H12C	109.5	Cl4—C99—H99B	109.3
C1—C13—H13A	109.5	Cl5—C99—H99B	109.3
C1—C13—H13B	109.5	H99A—C99—H99B	108.0
H13A—C13—H13B	109.5		
			
C3—P1—S1—Au1	−78.75 (7)	S1—P1—C2—C22	66.86 (15)
C2—P1—S1—Au1	46.80 (8)	C3—P1—C2—C23	73.86 (16)
C1—P1—S1—Au1	164.36 (6)	C1—P1—C2—C23	−160.53 (14)
Cl3—Au1—S1—P1	82.19 (3)	S1—P1—C2—C23	−50.49 (16)
Cl2—Au1—S1—P1	−100.28 (3)	C3—P1—C2—C21	−46.91 (17)
C3—P1—C1—C12	−41.56 (16)	C1—P1—C2—C21	78.70 (17)
C2—P1—C1—C12	−167.75 (13)	S1—P1—C2—C21	−171.26 (13)
S1—P1—C1—C12	74.48 (14)	C2—P1—C3—C31	77.86 (17)
C3—P1—C1—C11	79.02 (16)	C1—P1—C3—C31	−47.81 (17)
C2—P1—C1—C11	−47.17 (16)	S1—P1—C3—C31	−156.98 (13)
S1—P1—C1—C11	−164.93 (13)	C2—P1—C3—C33	−42.66 (16)
C3—P1—C1—C13	−159.43 (13)	C1—P1—C3—C33	−168.32 (13)
C2—P1—C1—C13	74.39 (16)	S1—P1—C3—C33	82.51 (13)
S1—P1—C1—C13	−43.38 (14)	C2—P1—C3—C32	−159.80 (13)
C3—P1—C2—C22	−168.80 (13)	C1—P1—C3—C32	74.54 (15)
C1—P1—C2—C22	−43.18 (16)	S1—P1—C3—C32	−34.64 (15)

### Trichlorido(tri-tert-butylphosphane sulfide-κS)gold(III) dichloromethane monosolvate (12a) Hydrogen-bond geometry (Å, º)

**Table:**

*D*—H···*A*	*D*—H	H···*A*	*D*···*A*	*D*—H···*A*
C23—H23*A*···Au1	0.98	2.69	3.438 (2)	134
C13—H13*B*···S1	0.98	2.61	3.131 (2)	114
C32—H32*A*···S1	0.98	2.83	3.323 (2)	112
C23—H23*A*···Cl2	0.98	2.82	3.778 (2)	168
C32—H32*A*···Cl3	0.98	2.88	3.759 (2)	150
C33—H33*B*···Cl3	0.98	2.73	3.623 (2)	152
C99—H99*A*···Cl2	0.99	2.84	3.749 (3)	153
C99—H99*B*···Cl3^i^	0.99	2.96	3.903 (3)	160
C22—H22*A*···Cl3^i^	0.98	2.82	3.791 (2)	171

Symmetry code: (i) *x*+1, *y*, *z*.

### (tert-Butyldipropan-2-ylphosphane selenide-κS)trichloridogold(III) (14a) Crystal data

**Table:**

[AuCl_3_(C_10_H_23_PSe)]	*F*(000) = 1048
*M**_r_* = 556.53	*D*_x_ = 2.231 Mg m^−^^3^
Monoclinic, *P*2_1_/*n*	Mo *K*α radiation, λ = 0.71073 Å
*a* = 7.92516 (18) Å	Cell parameters from 44121 reflections
*b* = 14.5559 (4) Å	θ = 2.8–29.0°
*c* = 14.3635 (4) Å	µ = 11.64 mm^−^^1^
β = 91.264 (2)°	*T* = 100 K
*V* = 1656.54 (7) Å^3^	Block, red
*Z* = 4	0.15 × 0.15 × 0.1 mm

### (tert-Butyldipropan-2-ylphosphane selenide-κS)trichloridogold(III) (14a) Data collection

**Table:**

Oxford Diffraction Xcalibur, Eos diffractometer	4112 independent reflections
Radiation source: Enhance (Mo) X-ray Source	3878 reflections with *I* > 2σ(*I*)
Graphite monochromator	*R*_int_ = 0.084
Detector resolution: 16.1419 pixels mm^-1^	θ_max_ = 28.3°, θ_min_ = 2.8°
ω scans	*h* = −10→10
Absorption correction: multi-scan (CrysAlisPro; Rigaku OD, 2020)	*k* = −19→19
*T*_min_ = 0.483, *T*_max_ = 1.000	*l* = −19→19
196706 measured reflections	

### (tert-Butyldipropan-2-ylphosphane selenide-κS)trichloridogold(III) (14a) Refinement

**Table:**

Refinement on *F*^2^	Primary atom site location: structure-invariant direct methods
Least-squares matrix: full	Hydrogen site location: inferred from neighbouring sites
*R*[*F*^2^ > 2σ(*F*^2^)] = 0.043	H-atom parameters constrained
*wR*(*F*^2^) = 0.093	*w* = 1/[σ^2^(*F*_o_^2^) + 30.9095*P*] where *P* = (*F*_o_^2^ + 2*F*_c_^2^)/3
*S* = 1.32	(Δ/σ)_max_ = 0.001
4112 reflections	Δρ_max_ = 3.81 e Å^−^^3^
152 parameters	Δρ_min_ = −2.41 e Å^−^^3^
0 restraints	

### (tert-Butyldipropan-2-ylphosphane selenide-κS)trichloridogold(III) (14a) Special details

**Table:**

Geometry. All esds (except the esd in the dihedral angle between two l.s. planes) are estimated using the full covariance matrix. The cell esds are taken into account individually in the estimation of esds in distances, angles and torsion angles; correlations between esds in cell parameters are only used when they are defined by crystal symmetry. An approximate (isotropic) treatment of cell esds is used for estimating esds involving l.s. planes.

### (tert-Butyldipropan-2-ylphosphane selenide-κS)trichloridogold(III) (14a) Fractional atomic coordinates and isotropic or equivalent isotropic displacement parameters (Å^2^)

**Table:**

	*x*	*y*	*z*	*U*_iso_*/*U*_eq_	
Au1	0.45922 (4)	0.06629 (2)	0.31589 (2)	0.01738 (8)	
Cl1	0.4313 (3)	0.01307 (15)	0.16364 (15)	0.0276 (4)	
Cl2	0.1741 (2)	0.08748 (16)	0.31991 (16)	0.0292 (5)	
Cl3	0.7414 (2)	0.03567 (14)	0.31613 (14)	0.0239 (4)	
Se1	0.47042 (10)	0.12099 (5)	0.47644 (6)	0.01951 (17)	
P1	0.6495 (2)	0.23732 (13)	0.48512 (13)	0.0145 (4)	
C1	0.6072 (12)	0.3269 (5)	0.3948 (6)	0.0240 (17)	
C2	0.6083 (10)	0.2813 (6)	0.6029 (6)	0.0207 (16)	
H2	0.693302	0.330393	0.617188	0.025*	
C3	0.8687 (10)	0.1988 (6)	0.4766 (6)	0.0240 (17)	
H3	0.883705	0.180911	0.410097	0.029*	
C11	0.6701 (12)	0.4210 (6)	0.4307 (6)	0.0270 (18)	
H11A	0.651327	0.467517	0.382269	0.041*	
H11B	0.790957	0.417208	0.446273	0.041*	
H11C	0.608111	0.438057	0.486405	0.041*	
C12	0.4190 (13)	0.3337 (7)	0.3704 (8)	0.039 (2)	
H12A	0.356324	0.347367	0.426822	0.058*	
H12B	0.379581	0.275247	0.343994	0.058*	
H12C	0.400336	0.382903	0.324754	0.058*	
C13	0.7024 (16)	0.3017 (7)	0.3058 (7)	0.040 (3)	
H13A	0.660326	0.242939	0.281512	0.061*	
H13B	0.823381	0.296514	0.320588	0.061*	
H13C	0.684125	0.349746	0.258870	0.061*	
C21	0.4334 (11)	0.3234 (6)	0.6129 (6)	0.0265 (18)	
H21A	0.347661	0.275184	0.606145	0.040*	
H21B	0.414959	0.370033	0.564578	0.040*	
H21C	0.425261	0.351900	0.674447	0.040*	
C22	0.6290 (15)	0.2065 (7)	0.6771 (6)	0.040 (3)	
H22A	0.600785	0.231711	0.738109	0.060*	
H22B	0.746135	0.184855	0.678801	0.060*	
H22C	0.553465	0.155102	0.662001	0.060*	
C31	0.9989 (13)	0.2759 (8)	0.4970 (10)	0.054 (3)	
H31A	0.987031	0.297574	0.561092	0.081*	
H31B	0.978980	0.327034	0.453743	0.081*	
H31C	1.113213	0.251872	0.488980	0.081*	
C32	0.9129 (13)	0.1140 (8)	0.5341 (7)	0.039 (3)	
H32A	1.014400	0.085151	0.509559	0.059*	
H32B	0.818852	0.070321	0.530678	0.059*	
H32C	0.933871	0.131909	0.599105	0.059*	

### (tert-Butyldipropan-2-ylphosphane selenide-κS)trichloridogold(III) (14a) Atomic displacement parameters (Å^2^)

**Table:**

	*U*^11^	*U*^22^	*U*^33^	*U*^12^	*U*^13^	*U*^23^
Au1	0.01636 (13)	0.01447 (14)	0.02152 (15)	−0.00068 (11)	0.00488 (10)	−0.00236 (11)
Cl1	0.0283 (10)	0.0306 (11)	0.0237 (10)	0.0010 (8)	−0.0005 (8)	−0.0059 (8)
Cl2	0.0167 (9)	0.0371 (12)	0.0340 (11)	0.0028 (8)	0.0031 (8)	−0.0006 (9)
Cl3	0.0179 (8)	0.0261 (10)	0.0281 (10)	0.0015 (7)	0.0052 (7)	−0.0103 (8)
Se1	0.0227 (4)	0.0153 (4)	0.0209 (4)	−0.0047 (3)	0.0098 (3)	−0.0021 (3)
P1	0.0151 (8)	0.0122 (8)	0.0162 (9)	0.0010 (7)	0.0047 (7)	−0.0006 (7)
C1	0.039 (5)	0.012 (3)	0.022 (4)	0.002 (3)	0.011 (3)	0.002 (3)
C2	0.022 (4)	0.019 (4)	0.021 (4)	0.002 (3)	0.006 (3)	−0.004 (3)
C3	0.017 (4)	0.024 (4)	0.031 (4)	0.005 (3)	0.003 (3)	−0.007 (4)
C11	0.034 (5)	0.016 (4)	0.031 (5)	0.001 (3)	0.011 (4)	0.004 (3)
C12	0.048 (6)	0.022 (5)	0.045 (6)	0.007 (4)	−0.017 (5)	0.003 (4)
C13	0.075 (8)	0.021 (5)	0.027 (5)	0.005 (5)	0.022 (5)	0.001 (4)
C21	0.029 (4)	0.027 (4)	0.024 (4)	0.005 (3)	0.013 (3)	−0.001 (3)
C22	0.064 (7)	0.041 (6)	0.016 (4)	0.021 (5)	0.001 (4)	−0.005 (4)
C31	0.025 (5)	0.047 (7)	0.091 (10)	0.004 (5)	0.002 (5)	−0.034 (7)
C32	0.036 (5)	0.050 (6)	0.031 (5)	0.031 (5)	0.003 (4)	0.003 (5)

### (tert-Butyldipropan-2-ylphosphane selenide-κS)trichloridogold(III) (14a) Geometric parameters (Å, º)

**Table:**

Au1—Cl3	2.2803 (19)	C1—C12	1.528 (13)
Au1—Cl2	2.283 (2)	C1—C13	1.543 (12)
Au1—Cl1	2.326 (2)	C1—C11	1.543 (12)
Au1—Se1	2.4393 (8)	C2—C21	1.524 (11)
Se1—P1	2.211 (2)	C2—C22	1.530 (13)
P1—C3	1.832 (8)	C3—C32	1.522 (13)
P1—C2	1.845 (8)	C3—C31	1.548 (13)
P1—C1	1.864 (8)		
			
Cl3—Au1—Cl2	176.18 (8)	C12—C1—C13	108.6 (9)
Cl3—Au1—Cl1	90.53 (7)	C12—C1—C11	109.0 (7)
Cl2—Au1—Cl1	89.72 (8)	C13—C1—C11	109.1 (7)
Cl3—Au1—Se1	92.70 (5)	C12—C1—P1	111.4 (6)
Cl2—Au1—Se1	86.98 (6)	C13—C1—P1	109.1 (6)
Cl1—Au1—Se1	176.59 (6)	C11—C1—P1	109.7 (6)
P1—Se1—Au1	108.25 (6)	C21—C2—C22	107.7 (7)
C3—P1—C2	110.8 (4)	C21—C2—P1	113.9 (6)
C3—P1—C1	108.9 (4)	C22—C2—P1	111.9 (6)
C2—P1—C1	111.3 (4)	C32—C3—C31	109.9 (9)
C3—P1—Se1	111.7 (3)	C32—C3—P1	114.8 (6)
C2—P1—Se1	101.0 (3)	C31—C3—P1	113.3 (6)
C1—P1—Se1	113.0 (3)		
			
Cl3—Au1—Se1—P1	58.66 (8)	Se1—P1—C1—C11	152.6 (5)
Cl2—Au1—Se1—P1	−125.16 (8)	C3—P1—C2—C21	174.3 (6)
Au1—Se1—P1—C3	−72.6 (3)	C1—P1—C2—C21	53.0 (7)
Au1—Se1—P1—C2	169.6 (3)	Se1—P1—C2—C21	−67.1 (6)
Au1—Se1—P1—C1	50.7 (3)	C3—P1—C2—C22	−63.2 (8)
C3—P1—C1—C12	156.7 (6)	C1—P1—C2—C22	175.5 (7)
C2—P1—C1—C12	−81.0 (7)	Se1—P1—C2—C22	55.3 (7)
Se1—P1—C1—C12	31.9 (7)	C2—P1—C3—C32	67.7 (8)
C3—P1—C1—C13	36.8 (8)	C1—P1—C3—C32	−169.6 (7)
C2—P1—C1—C13	159.2 (7)	Se1—P1—C3—C32	−44.0 (7)
Se1—P1—C1—C13	−88.0 (7)	C2—P1—C3—C31	−59.6 (9)
C3—P1—C1—C11	−82.6 (6)	C1—P1—C3—C31	63.0 (9)
C2—P1—C1—C11	39.8 (7)	Se1—P1—C3—C31	−171.4 (7)

### (tert-Butyldipropan-2-ylphosphane selenide-κS)trichloridogold(III) (14a) Hydrogen-bond geometry (Å, º)

**Table:**

*D*—H···*A*	*D*—H	H···*A*	*D*···*A*	*D*—H···*A*
C3—H3···Cl3	1.00	2.74	3.445 (9)	128
C3—H3···Cl2^i^	1.00	2.99	3.714 (8)	130
C21—H21*C*···Cl2^ii^	0.98	2.98	3.730 (10)	135
C22—H22*A*···Cl2^ii^	0.98	2.94	3.646 (11)	130
C13—H13*C*···Cl3^iii^	0.98	2.98	3.860 (10)	151

Symmetry codes: (i) *x*+1, *y*, *z*; (ii) *x*+1/2, −*y*+1/2, *z*+1/2; (iii) −*x*+3/2, *y*+1/2, −*z*+1/2.

### Trichlorido[di-tert-butyl(propan-2-yl)phosphane selenide-κS]gold(III) (15a) Crystal data

**Table:**

[AuCl_3_(C_11_H_25_PSe)]	*Z* = 2
*M**_r_* = 570.55	*F*(000) = 540
Triclinic, *P*1	*D*_x_ = 2.164 Mg m^−^^3^
*a* = 8.5878 (4) Å	Mo *K*α radiation, λ = 0.71073 Å
*b* = 9.8435 (4) Å	Cell parameters from 26441 reflections
*c* = 11.5022 (5) Å	θ = 2.2–30.8°
α = 78.391 (3)°	µ = 11.01 mm^−^^1^
β = 71.168 (4)°	*T* = 100 K
γ = 73.463 (4)°	Block, dark red
*V* = 875.78 (7) Å^3^	0.18 × 0.15 × 0.12 mm

### Trichlorido[di-tert-butyl(propan-2-yl)phosphane selenide-κS]gold(III) (15a) Data collection

**Table:**

Oxford Diffraction Xcalibur, Eos diffractometer	5231 independent reflections
Radiation source: Enhance (Mo) X-ray Source	4984 reflections with *I* > 2σ(*I*)
Detector resolution: 16.1419 pixels mm^-1^	*R*_int_ = 0.032
ω scans	θ_max_ = 30.9°, θ_min_ = 2.2°
Absorption correction: multi-scan (CrysAlisPro; Rigaku OD, 2020)	*h* = −12→12
*T*_min_ = 0.700, *T*_max_ = 1.000	*k* = −14→14
54532 measured reflections	*l* = −16→16

### Trichlorido[di-tert-butyl(propan-2-yl)phosphane selenide-κS]gold(III) (15a) Refinement

**Table:**

Refinement on *F*^2^	Secondary atom site location: difference Fourier map
Least-squares matrix: full	Hydrogen site location: inferred from neighbouring sites
*R*[*F*^2^ > 2σ(*F*^2^)] = 0.014	H-atom parameters constrained
*wR*(*F*^2^) = 0.030	*w* = 1/[σ^2^(*F*_o_^2^) + (0.0102*P*)^2^ + 0.6517*P*] where *P* = (*F*_o_^2^ + 2*F*_c_^2^)/3
*S* = 1.10	(Δ/σ)_max_ = 0.002
5231 reflections	Δρ_max_ = 0.84 e Å^−^^3^
163 parameters	Δρ_min_ = −0.73 e Å^−^^3^
0 restraints	Extinction correction: SHELXL-2019/3 (Sheldrick, 2015), *F*_c_^*^ = *kF*_c_[1 + 0.001 *F*_c_^2^λ^3^/sin(2θ)]^-1/4^
Primary atom site location: structure-invariant direct methods	Extinction coefficient: 0.00113 (8)

### Trichlorido[di-tert-butyl(propan-2-yl)phosphane selenide-κS]gold(III) (15a) Fractional atomic coordinates and isotropic or equivalent isotropic displacement parameters (Å^2^)

**Table:**

	*x*	*y*	*z*	*U*_iso_*/*U*_eq_	
Au1	0.47065 (2)	0.34817 (2)	0.24083 (2)	0.01225 (3)	
Cl1	0.53440 (7)	0.12047 (5)	0.34417 (5)	0.02266 (10)	
Cl2	0.73944 (6)	0.36884 (5)	0.21249 (4)	0.01954 (9)	
Cl3	0.20708 (6)	0.32192 (5)	0.25905 (5)	0.02097 (9)	
Se1	0.41932 (2)	0.58622 (2)	0.12550 (2)	0.01264 (4)	
P1	0.21270 (5)	0.73111 (5)	0.24893 (4)	0.00884 (8)	
C1	0.2579 (2)	0.71444 (18)	0.40126 (15)	0.0110 (3)	
C2	0.2204 (2)	0.90901 (18)	0.15358 (16)	0.0119 (3)	
C3	0.0106 (2)	0.68238 (19)	0.27683 (17)	0.0139 (3)	
H3	0.029641	0.580448	0.314717	0.017*	
C11	0.1588 (2)	0.8456 (2)	0.47037 (16)	0.0154 (3)	
H11A	0.178588	0.829709	0.551846	0.023*	
H11B	0.037554	0.860209	0.480689	0.023*	
H11C	0.197124	0.930249	0.422547	0.023*	
C12	0.4474 (2)	0.6965 (2)	0.38347 (17)	0.0160 (4)	
H12A	0.467866	0.684501	0.464383	0.024*	
H12B	0.482936	0.781360	0.333425	0.024*	
H12C	0.512449	0.612140	0.341234	0.024*	
C13	0.2056 (2)	0.58019 (19)	0.48112 (16)	0.0151 (3)	
H13A	0.261624	0.497634	0.434914	0.023*	
H13B	0.082529	0.594352	0.501794	0.023*	
H13C	0.239410	0.563197	0.557428	0.023*	
C21	0.2454 (3)	0.8959 (2)	0.01674 (16)	0.0171 (4)	
H21A	0.235623	0.990931	−0.030806	0.026*	
H21B	0.158520	0.852840	0.011331	0.026*	
H21C	0.357828	0.835579	−0.017068	0.026*	
C22	0.0541 (2)	1.01974 (19)	0.19973 (17)	0.0164 (4)	
H22A	0.031062	1.022339	0.288534	0.025*	
H22B	−0.039081	0.993275	0.185004	0.025*	
H22C	0.064293	1.114141	0.155043	0.025*	
C23	0.3698 (2)	0.9605 (2)	0.15942 (17)	0.0164 (4)	
H23A	0.377807	1.049299	0.103710	0.025*	
H23B	0.474790	0.887482	0.134006	0.025*	
H23C	0.352286	0.977511	0.244277	0.025*	
C31	−0.0328 (3)	0.6819 (2)	0.15753 (19)	0.0222 (4)	
H31A	−0.121644	0.630111	0.176110	0.033*	
H31B	0.068516	0.634926	0.097399	0.033*	
H31C	−0.072920	0.780409	0.122908	0.033*	
C32	−0.1437 (2)	0.7623 (2)	0.37100 (19)	0.0191 (4)	
H32A	−0.169270	0.864012	0.339953	0.029*	
H32B	−0.118272	0.750211	0.450156	0.029*	
H32C	−0.241604	0.723426	0.382853	0.029*	

### Trichlorido[di-tert-butyl(propan-2-yl)phosphane selenide-κS]gold(III) (15a) Atomic displacement parameters (Å^2^)

**Table:**

	*U*^11^	*U*^22^	*U*^33^	*U*^12^	*U*^13^	*U*^23^
Au1	0.01513 (4)	0.00940 (3)	0.01254 (4)	0.00065 (2)	−0.00576 (2)	−0.00410 (2)
Cl1	0.0321 (3)	0.0122 (2)	0.0273 (2)	−0.00214 (18)	−0.0176 (2)	0.00029 (17)
Cl2	0.0152 (2)	0.0192 (2)	0.0244 (2)	0.00072 (16)	−0.00806 (17)	−0.00586 (17)
Cl3	0.0193 (2)	0.0132 (2)	0.0327 (3)	−0.00315 (16)	−0.00950 (19)	−0.00519 (18)
Se1	0.01521 (8)	0.01085 (8)	0.00931 (7)	0.00077 (6)	−0.00215 (6)	−0.00334 (6)
P1	0.00901 (19)	0.00871 (19)	0.00890 (18)	−0.00114 (15)	−0.00311 (15)	−0.00166 (14)
C1	0.0116 (8)	0.0127 (8)	0.0084 (7)	−0.0003 (6)	−0.0034 (6)	−0.0031 (6)
C2	0.0139 (8)	0.0100 (7)	0.0116 (8)	−0.0028 (6)	−0.0038 (6)	−0.0006 (6)
C3	0.0121 (8)	0.0128 (8)	0.0183 (8)	−0.0040 (6)	−0.0071 (7)	0.0009 (6)
C11	0.0169 (8)	0.0144 (8)	0.0133 (8)	0.0007 (7)	−0.0031 (7)	−0.0062 (6)
C12	0.0123 (8)	0.0210 (9)	0.0159 (8)	0.0001 (7)	−0.0066 (7)	−0.0065 (7)
C13	0.0173 (8)	0.0148 (8)	0.0110 (8)	−0.0013 (7)	−0.0037 (7)	−0.0007 (6)
C21	0.0238 (10)	0.0153 (8)	0.0117 (8)	−0.0047 (7)	−0.0061 (7)	0.0015 (6)
C22	0.0171 (9)	0.0109 (8)	0.0195 (9)	0.0004 (7)	−0.0066 (7)	−0.0007 (7)
C23	0.0169 (9)	0.0154 (8)	0.0171 (9)	−0.0062 (7)	−0.0027 (7)	−0.0026 (7)
C31	0.0235 (10)	0.0226 (10)	0.0275 (10)	−0.0089 (8)	−0.0157 (8)	0.0004 (8)
C32	0.0098 (8)	0.0177 (9)	0.0263 (10)	−0.0019 (7)	−0.0038 (7)	0.0011 (7)

### Trichlorido[di-tert-butyl(propan-2-yl)phosphane selenide-κS]gold(III) (15a) Geometric parameters (Å, º)

**Table:**

Au1—Cl2	2.2871 (5)	C12—H12B	0.9800
Au1—Cl3	2.2889 (5)	C12—H12C	0.9800
Au1—Cl1	2.3207 (5)	C13—H13A	0.9800
Au1—Se1	2.4460 (2)	C13—H13B	0.9800
Se1—P1	2.2240 (5)	C13—H13C	0.9800
P1—C3	1.8435 (18)	C21—H21A	0.9800
P1—C2	1.8762 (18)	C21—H21B	0.9800
P1—C1	1.8802 (17)	C21—H21C	0.9800
C1—C12	1.535 (2)	C22—H22A	0.9800
C1—C11	1.541 (2)	C22—H22B	0.9800
C1—C13	1.541 (2)	C22—H22C	0.9800
C2—C23	1.532 (3)	C23—H23A	0.9800
C2—C22	1.539 (2)	C23—H23B	0.9800
C2—C21	1.545 (2)	C23—H23C	0.9800
C3—C31	1.535 (3)	C31—H31A	0.9800
C3—C32	1.539 (3)	C31—H31B	0.9800
C3—H3	1.0000	C31—H31C	0.9800
C11—H11A	0.9800	C32—H32A	0.9800
C11—H11B	0.9800	C32—H32B	0.9800
C11—H11C	0.9800	C32—H32C	0.9800
C12—H12A	0.9800		
			
Cl2—Au1—Cl3	176.794 (17)	C1—C12—H12C	109.5
Cl2—Au1—Cl1	89.966 (18)	H12A—C12—H12C	109.5
Cl3—Au1—Cl1	89.946 (18)	H12B—C12—H12C	109.5
Cl2—Au1—Se1	87.383 (14)	C1—C13—H13A	109.5
Cl3—Au1—Se1	92.617 (13)	C1—C13—H13B	109.5
Cl1—Au1—Se1	176.951 (14)	H13A—C13—H13B	109.5
P1—Se1—Au1	108.487 (14)	C1—C13—H13C	109.5
C3—P1—C2	112.81 (8)	H13A—C13—H13C	109.5
C3—P1—C1	108.96 (8)	H13B—C13—H13C	109.5
C2—P1—C1	114.08 (8)	C2—C21—H21A	109.5
C3—P1—Se1	109.45 (6)	C2—C21—H21B	109.5
C2—P1—Se1	101.19 (6)	H21A—C21—H21B	109.5
C1—P1—Se1	110.07 (5)	C2—C21—H21C	109.5
C12—C1—C11	108.31 (15)	H21A—C21—H21C	109.5
C12—C1—C13	108.25 (14)	H21B—C21—H21C	109.5
C11—C1—C13	109.14 (14)	C2—C22—H22A	109.5
C12—C1—P1	111.25 (11)	C2—C22—H22B	109.5
C11—C1—P1	111.89 (12)	H22A—C22—H22B	109.5
C13—C1—P1	107.91 (12)	C2—C22—H22C	109.5
C23—C2—C22	109.72 (15)	H22A—C22—H22C	109.5
C23—C2—C21	107.75 (14)	H22B—C22—H22C	109.5
C22—C2—C21	108.65 (15)	C2—C23—H23A	109.5
C23—C2—P1	110.50 (12)	C2—C23—H23B	109.5
C22—C2—P1	110.36 (12)	H23A—C23—H23B	109.5
C21—C2—P1	109.82 (12)	C2—C23—H23C	109.5
C31—C3—C32	110.83 (15)	H23A—C23—H23C	109.5
C31—C3—P1	113.41 (13)	H23B—C23—H23C	109.5
C32—C3—P1	116.45 (13)	C3—C31—H31A	109.5
C31—C3—H3	105.0	C3—C31—H31B	109.5
C32—C3—H3	105.0	H31A—C31—H31B	109.5
P1—C3—H3	105.0	C3—C31—H31C	109.5
C1—C11—H11A	109.5	H31A—C31—H31C	109.5
C1—C11—H11B	109.5	H31B—C31—H31C	109.5
H11A—C11—H11B	109.5	C3—C32—H32A	109.5
C1—C11—H11C	109.5	C3—C32—H32B	109.5
H11A—C11—H11C	109.5	H32A—C32—H32B	109.5
H11B—C11—H11C	109.5	C3—C32—H32C	109.5
C1—C12—H12A	109.5	H32A—C32—H32C	109.5
C1—C12—H12B	109.5	H32B—C32—H32C	109.5
H12A—C12—H12B	109.5		
			
Cl2—Au1—Se1—P1	−118.493 (18)	C1—P1—C2—C23	41.28 (14)
Cl3—Au1—Se1—P1	64.717 (19)	Se1—P1—C2—C23	−76.86 (12)
Au1—Se1—P1—C3	−69.97 (6)	C3—P1—C2—C22	44.79 (15)
Au1—Se1—P1—C2	170.76 (6)	C1—P1—C2—C22	−80.24 (14)
Au1—Se1—P1—C1	49.76 (6)	Se1—P1—C2—C22	161.62 (11)
C3—P1—C1—C12	158.15 (12)	C3—P1—C2—C21	−74.96 (14)
C2—P1—C1—C12	−74.80 (14)	C1—P1—C2—C21	160.01 (12)
Se1—P1—C1—C12	38.13 (14)	Se1—P1—C2—C21	41.87 (13)
C3—P1—C1—C11	−80.54 (14)	C2—P1—C3—C31	56.35 (16)
C2—P1—C1—C11	46.50 (15)	C1—P1—C3—C31	−175.89 (13)
Se1—P1—C1—C11	159.43 (11)	Se1—P1—C3—C31	−55.48 (14)
C3—P1—C1—C13	39.54 (14)	C2—P1—C3—C32	−74.05 (15)
C2—P1—C1—C13	166.59 (11)	C1—P1—C3—C32	53.71 (15)
Se1—P1—C1—C13	−80.49 (11)	Se1—P1—C3—C32	174.13 (12)
C3—P1—C2—C23	166.31 (12)		

### Trichlorido[di-tert-butyl(propan-2-yl)phosphane selenide-κS]gold(III) (15a) Hydrogen-bond geometry (Å, º)

**Table:**

*D*—H···*A*	*D*—H	H···*A*	*D*···*A*	*D*—H···*A*
C13—H13*A*···Au1	0.98	2.76	3.6818 (18)	158
C21—H21*C*···Se1	0.98	2.68	3.1887 (19)	113
C12—H12*C*···Se1	0.98	2.92	3.4566 (18)	116
C3—H3···Cl3	1.00	2.65	3.4842 (19)	141
C12—H12*C*···Cl2	0.98	2.94	3.9145 (19)	174
C13—H13*C*···Cl2^i^	0.98	2.93	3.8489 (19)	157

Symmetry code: (i) −*x*+1, −*y*+1, −*z*+1.

### Trichlorido[di-tert-butyl(propan-2-yl)phosphane selenide-κS]gold(III) chloroform-d monosolvate (15aa) Crystal data

**Table:**

[AuCl_3_(C_11_H_25_PSe)]·CDCl_3_	*Z* = 2
*M**_r_* = 690.93	*F*(000) = 656
Triclinic, *P*1	*D*_x_ = 2.152 Mg m^−^^3^
*a* = 8.5343 (2) Å	Mo *K*α radiation, λ = 0.71073 Å
*b* = 9.7185 (3) Å	Cell parameters from 34566 reflections
*c* = 14.0759 (4) Å	θ = 2.2–30.8°
α = 74.398 (2)°	µ = 9.42 mm^−^^1^
β = 78.121 (2)°	*T* = 100 K
γ = 73.257 (2)°	Tablet, red
*V* = 1066.31 (5) Å^3^	0.4 × 0.25 × 0.08 mm

### Trichlorido[di-tert-butyl(propan-2-yl)phosphane selenide-κS]gold(III) chloroform-d monosolvate (15aa) Data collection

**Table:**

Oxford Diffraction Xcalibur, Eos diffractometer	6287 independent reflections
Radiation source: Enhance (Mo) X-ray Source	6060 reflections with *I* > 2σ(*I*)
Detector resolution: 16.1419 pixels mm^-1^	*R*_int_ = 0.040
ω scans	θ_max_ = 30.8°, θ_min_ = 2.3°
Absorption correction: multi-scan (CrysAlisPro; Rigaku OD, 2020)	*h* = −12→11
*T*_min_ = 0.151, *T*_max_ = 1.000	*k* = −13→13
76069 measured reflections	*l* = −20→20

### Trichlorido[di-tert-butyl(propan-2-yl)phosphane selenide-κS]gold(III) chloroform-d monosolvate (15aa) Refinement

**Table:**

Refinement on *F*^2^	Primary atom site location: structure-invariant direct methods
Least-squares matrix: full	Secondary atom site location: difference Fourier map
*R*[*F*^2^ > 2σ(*F*^2^)] = 0.021	Hydrogen site location: inferred from neighbouring sites
*wR*(*F*^2^) = 0.048	H-atom parameters constrained
*S* = 1.10	*w* = 1/[σ^2^(*F*_o_^2^) + (0.0179*P*)^2^ + 2.4716*P*] where *P* = (*F*_o_^2^ + 2*F*_c_^2^)/3
6287 reflections	(Δ/σ)_max_ = 0.001
235 parameters	Δρ_max_ = 1.88 e Å^−^^3^
39 restraints	Δρ_min_ = −1.30 e Å^−^^3^

### Trichlorido[di-tert-butyl(propan-2-yl)phosphane selenide-κS]gold(III) chloroform-d monosolvate (15aa) Fractional atomic coordinates and isotropic or equivalent isotropic displacement parameters (Å^2^)

**Table:**

	*x*	*y*	*z*	*U*_iso_*/*U*_eq_	Occ. (<1)
Au1	0.19009 (2)	0.76455 (2)	0.29588 (2)	0.01280 (3)	
Se1	0.31024 (3)	0.58178 (3)	0.19374 (2)	0.01509 (5)	
Cl1	0.06715 (8)	0.94493 (7)	0.38594 (5)	0.02097 (13)	
Cl2	−0.06581 (8)	0.75760 (7)	0.27413 (5)	0.01900 (12)	
Cl3	0.44411 (8)	0.78057 (7)	0.31290 (5)	0.02113 (13)	
P1	0.43631 (7)	0.37535 (7)	0.29102 (4)	0.00998 (11)	
C1	0.2963 (3)	0.3257 (3)	0.40964 (18)	0.0121 (4)	
C2	0.4876 (3)	0.2413 (3)	0.20853 (19)	0.0145 (4)	
C3	0.6212 (3)	0.4055 (3)	0.32273 (19)	0.0147 (5)	
H3	0.579371	0.492572	0.354051	0.018*	
C11	0.3539 (3)	0.1623 (3)	0.46191 (19)	0.0164 (5)	
H11A	0.280779	0.141114	0.524606	0.025*	
H11B	0.467264	0.142210	0.475766	0.025*	
H11C	0.350289	0.099770	0.418588	0.025*	
C12	0.1176 (3)	0.3544 (3)	0.3902 (2)	0.0174 (5)	
H12A	0.047017	0.330491	0.453586	0.026*	
H12B	0.114078	0.292454	0.346449	0.026*	
H12C	0.077929	0.458417	0.358281	0.026*	
C13	0.2968 (3)	0.4237 (3)	0.47907 (18)	0.0155 (5)	
H13A	0.258643	0.527574	0.446277	0.023*	
H13B	0.409134	0.405259	0.494072	0.023*	
H13C	0.222798	0.400609	0.541098	0.023*	
C21	0.5491 (4)	0.3147 (3)	0.1006 (2)	0.0231 (6)	
H21A	0.587049	0.240826	0.059935	0.035*	
H21B	0.640676	0.356461	0.100995	0.035*	
H21C	0.458495	0.393467	0.072699	0.035*	
C22	0.6250 (3)	0.1067 (3)	0.2467 (2)	0.0196 (5)	
H22A	0.590896	0.063570	0.316669	0.029*	
H22B	0.726761	0.137778	0.240897	0.029*	
H22C	0.644756	0.033218	0.206881	0.029*	
C23	0.3345 (4)	0.1911 (3)	0.2054 (2)	0.0201 (5)	
H23A	0.361551	0.127902	0.157554	0.030*	
H23B	0.245281	0.277625	0.184829	0.030*	
H23C	0.298648	0.135850	0.271644	0.030*	
C31	0.7423 (4)	0.4521 (3)	0.2310 (2)	0.0238 (6)	
H31A	0.816732	0.497748	0.249454	0.036*	
H31B	0.680530	0.523186	0.179360	0.036*	
H31C	0.807063	0.365276	0.205380	0.036*	
C32	0.7134 (3)	0.2816 (3)	0.4007 (2)	0.0192 (5)	
H32A	0.756384	0.191589	0.374977	0.029*	
H32B	0.637176	0.262735	0.462303	0.029*	
H32C	0.805331	0.311493	0.414288	0.029*	
C99	−0.1097 (9)	0.8164 (8)	0.0177 (5)	0.0278 (16)	0.525 (4)
D99	−0.055137	0.830295	0.069726	0.033*	0.525 (4)
Cl4	−0.3237 (7)	0.8542 (6)	0.0573 (4)	0.0381 (9)	0.525 (4)
Cl5	−0.0625 (3)	0.9393 (2)	−0.09447 (12)	0.0435 (7)	0.525 (4)
Cl6	−0.0308 (2)	0.6342 (3)	0.00484 (14)	0.0420 (6)	0.525 (4)
C99'	−0.1635 (10)	0.8634 (8)	0.0319 (6)	0.0296 (19)	0.475 (4)
D99'	−0.126484	0.882844	0.088950	0.036*	0.475 (4)
Cl4'	−0.3518 (8)	0.8272 (8)	0.0703 (6)	0.0483 (14)	0.475 (4)
Cl5'	−0.1902 (5)	1.0248 (3)	−0.06191 (19)	0.0700 (13)	0.475 (4)
Cl6'	−0.0189 (3)	0.7176 (4)	−0.0073 (3)	0.0579 (8)	0.475 (4)

### Trichlorido[di-tert-butyl(propan-2-yl)phosphane selenide-κS]gold(III) chloroform-d monosolvate (15aa) Atomic displacement parameters (Å^2^)

**Table:**

	*U*^11^	*U*^22^	*U*^33^	*U*^12^	*U*^13^	*U*^23^
Au1	0.01412 (5)	0.00834 (5)	0.01369 (5)	−0.00048 (3)	−0.00226 (3)	−0.00097 (3)
Se1	0.01999 (12)	0.01043 (11)	0.01201 (11)	0.00044 (9)	−0.00387 (9)	−0.00108 (8)
Cl1	0.0216 (3)	0.0152 (3)	0.0250 (3)	−0.0012 (2)	0.0004 (2)	−0.0090 (2)
Cl2	0.0154 (3)	0.0172 (3)	0.0222 (3)	−0.0003 (2)	−0.0059 (2)	−0.0025 (2)
Cl3	0.0174 (3)	0.0139 (3)	0.0333 (3)	−0.0034 (2)	−0.0038 (2)	−0.0075 (2)
P1	0.0093 (3)	0.0086 (3)	0.0116 (3)	−0.0015 (2)	−0.0014 (2)	−0.0022 (2)
C1	0.0100 (10)	0.0103 (10)	0.0133 (10)	−0.0010 (8)	−0.0008 (8)	−0.0002 (8)
C2	0.0171 (11)	0.0118 (11)	0.0153 (11)	−0.0031 (9)	−0.0015 (9)	−0.0055 (9)
C3	0.0114 (10)	0.0151 (11)	0.0201 (12)	−0.0048 (9)	−0.0016 (9)	−0.0070 (9)
C11	0.0159 (11)	0.0117 (11)	0.0180 (11)	−0.0022 (9)	−0.0026 (9)	0.0015 (9)
C12	0.0096 (10)	0.0185 (12)	0.0219 (12)	−0.0037 (9)	−0.0021 (9)	−0.0009 (10)
C13	0.0158 (11)	0.0153 (11)	0.0133 (11)	−0.0011 (9)	−0.0005 (9)	−0.0039 (9)
C21	0.0325 (15)	0.0219 (13)	0.0133 (11)	−0.0051 (12)	0.0026 (10)	−0.0073 (10)
C22	0.0189 (12)	0.0150 (12)	0.0246 (13)	0.0015 (10)	−0.0036 (10)	−0.0096 (10)
C23	0.0238 (13)	0.0179 (12)	0.0227 (13)	−0.0058 (10)	−0.0082 (10)	−0.0070 (10)
C31	0.0191 (13)	0.0278 (15)	0.0274 (14)	−0.0140 (11)	0.0043 (11)	−0.0077 (12)
C32	0.0111 (11)	0.0204 (13)	0.0272 (13)	0.0009 (9)	−0.0078 (10)	−0.0084 (11)
C99	0.033 (4)	0.037 (5)	0.017 (3)	−0.018 (4)	−0.003 (3)	−0.002 (3)
Cl4	0.044 (2)	0.0402 (17)	0.0314 (16)	−0.0052 (13)	−0.0013 (12)	−0.0194 (15)
Cl5	0.0657 (14)	0.0527 (13)	0.0210 (7)	−0.0413 (11)	−0.0127 (8)	0.0107 (7)
Cl6	0.0357 (9)	0.0418 (13)	0.0336 (8)	−0.0004 (9)	0.0073 (7)	−0.0026 (8)
C99'	0.044 (5)	0.024 (4)	0.016 (3)	−0.002 (3)	−0.002 (3)	−0.005 (3)
Cl4'	0.040 (2)	0.058 (3)	0.041 (2)	−0.0050 (17)	−0.0056 (15)	−0.0084 (18)
Cl5'	0.141 (3)	0.0371 (13)	0.0460 (14)	−0.0363 (17)	−0.0559 (18)	0.0142 (10)
Cl6'	0.0431 (13)	0.0568 (19)	0.0751 (18)	0.0098 (13)	−0.0053 (11)	−0.0429 (16)

### Trichlorido[di-tert-butyl(propan-2-yl)phosphane selenide-κS]gold(III) chloroform-d monosolvate (15aa) Geometric parameters (Å, º)

**Table:**

Au1—Cl3	2.2825 (7)	C13—H13C	0.9800
Au1—Cl2	2.2889 (6)	C21—H21A	0.9800
Au1—Cl1	2.3172 (6)	C21—H21B	0.9800
Au1—Se1	2.4476 (3)	C21—H21C	0.9800
Se1—P1	2.2232 (6)	C22—H22A	0.9800
P1—C3	1.844 (3)	C22—H22B	0.9800
P1—C2	1.874 (2)	C22—H22C	0.9800
P1—C1	1.878 (2)	C23—H23A	0.9800
C1—C12	1.537 (3)	C23—H23B	0.9800
C1—C13	1.538 (3)	C23—H23C	0.9800
C1—C11	1.541 (3)	C31—H31A	0.9800
C2—C23	1.533 (4)	C31—H31B	0.9800
C2—C22	1.540 (4)	C31—H31C	0.9800
C2—C21	1.548 (4)	C32—H32A	0.9800
C3—C31	1.532 (4)	C32—H32B	0.9800
C3—C32	1.540 (4)	C32—H32C	0.9800
C3—H3	1.0000	C99—Cl6	1.748 (7)
C11—H11A	0.9800	C99—Cl4	1.756 (8)
C11—H11B	0.9800	C99—Cl5	1.756 (7)
C11—H11C	0.9800	C99—D99	1.0000
C12—H12A	0.9800	C99'—Cl4'	1.692 (9)
C12—H12B	0.9800	C99'—Cl6'	1.723 (7)
C12—H12C	0.9800	C99'—Cl5'	1.749 (8)
C13—H13A	0.9800	C99'—D99'	1.0000
C13—H13B	0.9800		
			
Cl3—Au1—Cl2	177.64 (2)	C1—C13—H13C	109.5
Cl3—Au1—Cl1	89.67 (2)	H13A—C13—H13C	109.5
Cl2—Au1—Cl1	89.66 (2)	H13B—C13—H13C	109.5
Cl3—Au1—Se1	92.332 (18)	C2—C21—H21A	109.5
Cl2—Au1—Se1	88.238 (18)	C2—C21—H21B	109.5
Cl1—Au1—Se1	176.855 (18)	H21A—C21—H21B	109.5
P1—Se1—Au1	107.617 (18)	C2—C21—H21C	109.5
C3—P1—C2	112.97 (12)	H21A—C21—H21C	109.5
C3—P1—C1	108.44 (11)	H21B—C21—H21C	109.5
C2—P1—C1	114.23 (11)	C2—C22—H22A	109.5
C3—P1—Se1	108.64 (9)	C2—C22—H22B	109.5
C2—P1—Se1	101.78 (8)	H22A—C22—H22B	109.5
C1—P1—Se1	110.54 (8)	C2—C22—H22C	109.5
C12—C1—C13	108.0 (2)	H22A—C22—H22C	109.5
C12—C1—C11	108.3 (2)	H22B—C22—H22C	109.5
C13—C1—C11	109.4 (2)	C2—C23—H23A	109.5
C12—C1—P1	111.35 (17)	C2—C23—H23B	109.5
C13—C1—P1	108.18 (17)	H23A—C23—H23B	109.5
C11—C1—P1	111.60 (16)	C2—C23—H23C	109.5
C23—C2—C22	109.8 (2)	H23A—C23—H23C	109.5
C23—C2—C21	107.5 (2)	H23B—C23—H23C	109.5
C22—C2—C21	108.7 (2)	C3—C31—H31A	109.5
C23—C2—P1	110.79 (18)	C3—C31—H31B	109.5
C22—C2—P1	109.77 (17)	H31A—C31—H31B	109.5
C21—C2—P1	110.23 (18)	C3—C31—H31C	109.5
C31—C3—C32	110.6 (2)	H31A—C31—H31C	109.5
C31—C3—P1	113.10 (19)	H31B—C31—H31C	109.5
C32—C3—P1	116.51 (18)	C3—C32—H32A	109.5
C31—C3—H3	105.2	C3—C32—H32B	109.5
C32—C3—H3	105.2	H32A—C32—H32B	109.5
P1—C3—H3	105.2	C3—C32—H32C	109.5
C1—C11—H11A	109.5	H32A—C32—H32C	109.5
C1—C11—H11B	109.5	H32B—C32—H32C	109.5
H11A—C11—H11B	109.5	Cl6—C99—Cl4	111.3 (4)
C1—C11—H11C	109.5	Cl6—C99—Cl5	110.4 (4)
H11A—C11—H11C	109.5	Cl4—C99—Cl5	110.9 (4)
H11B—C11—H11C	109.5	Cl6—C99—D99	108.0
C1—C12—H12A	109.5	Cl4—C99—D99	108.0
C1—C12—H12B	109.5	Cl5—C99—D99	108.0
H12A—C12—H12B	109.5	Cl4'—C99'—Cl6'	111.9 (5)
C1—C12—H12C	109.5	Cl4'—C99'—Cl5'	106.4 (5)
H12A—C12—H12C	109.5	Cl6'—C99'—Cl5'	112.7 (5)
H12B—C12—H12C	109.5	Cl4'—C99'—D99'	108.6
C1—C13—H13A	109.5	Cl6'—C99'—D99'	108.6
C1—C13—H13B	109.5	Cl5'—C99'—D99'	108.6
H13A—C13—H13B	109.5		
			
Cl3—Au1—Se1—P1	68.16 (3)	C1—P1—C2—C23	41.5 (2)
Cl2—Au1—Se1—P1	−114.14 (3)	Se1—P1—C2—C23	−77.67 (18)
Au1—Se1—P1—C3	−69.90 (9)	C3—P1—C2—C22	44.6 (2)
Au1—Se1—P1—C2	170.69 (8)	C1—P1—C2—C22	−80.0 (2)
Au1—Se1—P1—C1	48.97 (9)	Se1—P1—C2—C22	160.85 (17)
C3—P1—C1—C12	159.02 (17)	C3—P1—C2—C21	−75.1 (2)
C2—P1—C1—C12	−74.0 (2)	C1—P1—C2—C21	160.34 (18)
Se1—P1—C1—C12	40.02 (19)	Se1—P1—C2—C21	41.2 (2)
C3—P1—C1—C13	40.54 (19)	C2—P1—C3—C31	54.3 (2)
C2—P1—C1—C13	167.49 (16)	C1—P1—C3—C31	−178.01 (19)
Se1—P1—C1—C13	−78.46 (16)	Se1—P1—C3—C31	−57.8 (2)
C3—P1—C1—C11	−79.9 (2)	C2—P1—C3—C32	−75.4 (2)
C2—P1—C1—C11	47.1 (2)	C1—P1—C3—C32	52.2 (2)
Se1—P1—C1—C11	161.13 (15)	Se1—P1—C3—C32	172.41 (16)
C3—P1—C2—C23	166.03 (18)		

### Trichlorido[di-tert-butyl(propan-2-yl)phosphane selenide-κS]gold(III) chloroform-d monosolvate (15aa) Hydrogen-bond geometry (Å, º)

**Table:**

*D*—H···*A*	*D*—H	H···*A*	*D*···*A*	*D*—H···*A*
C99—D99···Cl2	1.00	2.76	3.580 (7)	139
C13—H13*A*···Au1	0.98	2.69	3.618 (3)	158
C21—H21*C*···Se1	0.98	2.70	3.208 (3)	112
C3—H3···Cl3	1.00	2.65	3.497 (3)	142
C13—H13*C*···Cl2^i^	0.98	2.92	3.866 (3)	163

Symmetry code: (i) −*x*, −*y*+1, −*z*+1.

### Tribromrido(tripropan-2-ylphosphane sulfide-κS)gold(III) (9b) Crystal data

**Table:**

[AuBr_3_(C_10_H_23_PS)]	*F*(000) = 1160
*M**_r_* = 628.98	*D*_x_ = 2.564 Mg m^−^^3^
Monoclinic, *P*2_1_/*c*	Mo *K*α radiation, λ = 0.71073 Å
*a* = 9.1341 (2) Å	Cell parameters from 22624 reflections
*b* = 7.9039 (2) Å	θ = 2.2–30.8°
*c* = 22.6420 (4) Å	µ = 16.58 mm^−^^1^
β = 94.519 (2)°	*T* = 100 K
*V* = 1629.56 (6) Å^3^	Plate, red
*Z* = 4	0.15 × 0.1 × 0.1 mm

### Tribromrido(tripropan-2-ylphosphane sulfide-κS)gold(III) (9b) Data collection

**Table:**

Oxford Diffraction Xcalibur, Eos diffractometer	4964 independent reflections
Radiation source: Enhance (Mo) X-ray Source	4633 reflections with *I* > 2σ(*I*)
Detector resolution: 16.1419 pixels mm^-1^	*R*_int_ = 0.037
ω scans	θ_max_ = 30.9°, θ_min_ = 2.2°
Absorption correction: multi-scan (CrysAlisPro; Rigaku OD, 2020)	*h* = −13→13
*T*_min_ = 0.486, *T*_max_ = 1.000	*k* = −11→11
64266 measured reflections	*l* = −32→31

### Tribromrido(tripropan-2-ylphosphane sulfide-κS)gold(III) (9b) Refinement

**Table:**

Refinement on *F*^2^	Secondary atom site location: difference Fourier map
Least-squares matrix: full	Hydrogen site location: inferred from neighbouring sites
*R*[*F*^2^ > 2σ(*F*^2^)] = 0.022	H-atom parameters constrained
*wR*(*F*^2^) = 0.038	*w* = 1/[σ^2^(*F*_o_^2^) + (0.007*P*)^2^ + 3.9739*P*] where *P* = (*F*_o_^2^ + 2*F*_c_^2^)/3
*S* = 1.23	(Δ/σ)_max_ = 0.003
4964 reflections	Δρ_max_ = 1.58 e Å^−^^3^
143 parameters	Δρ_min_ = −0.95 e Å^−^^3^
0 restraints	Extinction correction: SHELXL-2019/3 (Sheldrick, 2015), *F*_c_^*^ = *kF*_c_[1 + 0.001 *F*_c_^2^λ^3^/sin(2θ)]^-1/4^
Primary atom site location: structure-invariant direct methods	Extinction coefficient: 0.00043 (2)

### Tribromrido(tripropan-2-ylphosphane sulfide-κS)gold(III) (9b) Fractional atomic coordinates and isotropic or equivalent isotropic displacement parameters (Å^2^)

**Table:**

	*x*	*y*	*z*	*U*_iso_*/*U*_eq_	
Au1	0.14463 (2)	0.72099 (2)	0.36757 (2)	0.01115 (3)	
Br1	−0.10011 (3)	0.84484 (4)	0.36034 (2)	0.02107 (7)	
Br3	0.11734 (3)	0.65623 (4)	0.26267 (2)	0.01764 (6)	
Br2	0.17679 (4)	0.81030 (4)	0.47063 (2)	0.02013 (7)	
P1	0.36854 (8)	0.35281 (10)	0.37465 (3)	0.01090 (13)	
S1	0.38356 (8)	0.61179 (9)	0.37761 (3)	0.01358 (13)	
C1	0.5535 (3)	0.2875 (4)	0.40279 (14)	0.0171 (6)	
H1	0.562788	0.316948	0.445876	0.021*	
C2	0.3126 (3)	0.2763 (4)	0.30005 (12)	0.0131 (5)	
H2	0.221692	0.340584	0.286679	0.016*	
C3	0.2412 (3)	0.2647 (4)	0.42503 (13)	0.0153 (6)	
H3	0.252010	0.138946	0.423206	0.018*	
C11	0.5728 (4)	0.0942 (5)	0.39940 (16)	0.0269 (8)	
H11A	0.577770	0.060273	0.357972	0.040*	
H11B	0.489227	0.038208	0.415724	0.040*	
H11C	0.663821	0.061200	0.422335	0.040*	
C12	0.6787 (3)	0.3807 (5)	0.37507 (15)	0.0256 (7)	
H12A	0.772171	0.354456	0.397475	0.038*	
H12B	0.661110	0.502875	0.376156	0.038*	
H12C	0.682852	0.344092	0.333872	0.038*	
C21	0.4268 (3)	0.3213 (4)	0.25618 (13)	0.0173 (6)	
H21A	0.514162	0.250385	0.264097	0.026*	
H21B	0.454273	0.440703	0.260875	0.026*	
H21C	0.384802	0.301537	0.215597	0.026*	
C22	0.2702 (3)	0.0890 (4)	0.29712 (14)	0.0173 (6)	
H22A	0.240437	0.057944	0.256012	0.026*	
H22B	0.188340	0.068959	0.321750	0.026*	
H22C	0.354579	0.020019	0.311729	0.026*	
C31	0.2794 (4)	0.3151 (5)	0.48959 (14)	0.0241 (7)	
H31A	0.276855	0.438616	0.493159	0.036*	
H31B	0.378065	0.274003	0.502450	0.036*	
H31C	0.207962	0.265013	0.514538	0.036*	
C32	0.0805 (3)	0.3034 (4)	0.40569 (14)	0.0184 (6)	
H32A	0.016833	0.226482	0.425981	0.028*	
H32B	0.063393	0.287823	0.362757	0.028*	
H32C	0.058238	0.420545	0.415930	0.028*	

### Tribromrido(tripropan-2-ylphosphane sulfide-κS)gold(III) (9b) Atomic displacement parameters (Å^2^)

**Table:**

	*U*^11^	*U*^22^	*U*^33^	*U*^12^	*U*^13^	*U*^23^
Au1	0.01252 (5)	0.01097 (5)	0.00987 (5)	0.00095 (4)	0.00031 (3)	0.00136 (4)
Br1	0.01612 (14)	0.02620 (16)	0.02102 (15)	0.00786 (12)	0.00227 (11)	0.00523 (13)
Br3	0.02175 (14)	0.01905 (15)	0.01134 (13)	0.00400 (11)	−0.00357 (10)	−0.00144 (11)
Br2	0.02871 (16)	0.02110 (16)	0.01053 (13)	0.00543 (12)	0.00127 (11)	−0.00117 (11)
P1	0.0092 (3)	0.0123 (3)	0.0112 (3)	0.0014 (3)	0.0009 (2)	0.0005 (3)
S1	0.0114 (3)	0.0131 (3)	0.0160 (3)	−0.0012 (2)	−0.0003 (2)	−0.0010 (3)
C1	0.0136 (13)	0.0215 (15)	0.0158 (14)	0.0045 (12)	−0.0021 (10)	−0.0017 (12)
C2	0.0128 (12)	0.0145 (13)	0.0119 (12)	−0.0007 (11)	0.0008 (10)	−0.0012 (11)
C3	0.0168 (13)	0.0150 (14)	0.0148 (13)	0.0004 (11)	0.0051 (11)	0.0042 (11)
C11	0.0230 (16)	0.0259 (18)	0.0300 (18)	0.0146 (14)	−0.0100 (14)	−0.0072 (15)
C12	0.0097 (14)	0.042 (2)	0.0248 (17)	0.0024 (13)	−0.0009 (12)	−0.0009 (15)
C21	0.0197 (14)	0.0202 (15)	0.0124 (13)	−0.0022 (12)	0.0036 (11)	−0.0003 (11)
C22	0.0161 (14)	0.0159 (14)	0.0197 (15)	−0.0013 (11)	0.0007 (11)	−0.0024 (12)
C31	0.0288 (17)	0.0304 (19)	0.0135 (14)	−0.0009 (14)	0.0045 (12)	0.0039 (13)
C32	0.0150 (13)	0.0180 (16)	0.0230 (15)	−0.0009 (11)	0.0071 (11)	0.0016 (12)

### Tribromrido(tripropan-2-ylphosphane sulfide-κS)gold(III) (9b) Geometric parameters (Å, º)

**Table:**

Au1—S1	2.3413 (7)	C11—H11B	0.9800
Au1—Br3	2.4233 (3)	C11—H11C	0.9800
Au1—Br2	2.4333 (3)	C12—H12A	0.9800
Au1—Br1	2.4341 (3)	C12—H12B	0.9800
P1—C2	1.828 (3)	C12—H12C	0.9800
P1—C3	1.830 (3)	C21—H21A	0.9800
P1—C1	1.832 (3)	C21—H21B	0.9800
P1—S1	2.0523 (10)	C21—H21C	0.9800
C1—C12	1.536 (4)	C22—H22A	0.9800
C1—C11	1.540 (5)	C22—H22B	0.9800
C1—H1	1.0000	C22—H22C	0.9800
C2—C22	1.531 (4)	C31—H31A	0.9800
C2—C21	1.538 (4)	C31—H31B	0.9800
C2—H2	1.0000	C31—H31C	0.9800
C3—C31	1.529 (4)	C32—H32A	0.9800
C3—C32	1.529 (4)	C32—H32B	0.9800
C3—H3	1.0000	C32—H32C	0.9800
C11—H11A	0.9800		
			
S1—Au1—Br3	92.317 (19)	C1—C11—H11C	109.5
S1—Au1—Br2	88.43 (2)	H11A—C11—H11C	109.5
Br3—Au1—Br2	175.188 (12)	H11B—C11—H11C	109.5
S1—Au1—Br1	177.35 (2)	C1—C12—H12A	109.5
Br3—Au1—Br1	89.771 (11)	C1—C12—H12B	109.5
Br2—Au1—Br1	89.350 (11)	H12A—C12—H12B	109.5
C2—P1—C3	107.76 (14)	C1—C12—H12C	109.5
C2—P1—C1	114.26 (14)	H12A—C12—H12C	109.5
C3—P1—C1	106.86 (14)	H12B—C12—H12C	109.5
C2—P1—S1	111.90 (10)	C2—C21—H21A	109.5
C3—P1—S1	113.79 (11)	C2—C21—H21B	109.5
C1—P1—S1	102.26 (11)	H21A—C21—H21B	109.5
P1—S1—Au1	107.77 (4)	C2—C21—H21C	109.5
C12—C1—C11	111.3 (3)	H21A—C21—H21C	109.5
C12—C1—P1	114.8 (2)	H21B—C21—H21C	109.5
C11—C1—P1	111.6 (2)	C2—C22—H22A	109.5
C12—C1—H1	106.2	C2—C22—H22B	109.5
C11—C1—H1	106.2	H22A—C22—H22B	109.5
P1—C1—H1	106.2	C2—C22—H22C	109.5
C22—C2—C21	112.2 (2)	H22A—C22—H22C	109.5
C22—C2—P1	114.3 (2)	H22B—C22—H22C	109.5
C21—C2—P1	111.5 (2)	C3—C31—H31A	109.5
C22—C2—H2	106.1	C3—C31—H31B	109.5
C21—C2—H2	106.1	H31A—C31—H31B	109.5
P1—C2—H2	106.1	C3—C31—H31C	109.5
C31—C3—C32	111.3 (3)	H31A—C31—H31C	109.5
C31—C3—P1	112.8 (2)	H31B—C31—H31C	109.5
C32—C3—P1	112.9 (2)	C3—C32—H32A	109.5
C31—C3—H3	106.4	C3—C32—H32B	109.5
C32—C3—H3	106.4	H32A—C32—H32B	109.5
P1—C3—H3	106.4	C3—C32—H32C	109.5
C1—C11—H11A	109.5	H32A—C32—H32C	109.5
C1—C11—H11B	109.5	H32B—C32—H32C	109.5
H11A—C11—H11B	109.5		
			
C2—P1—S1—Au1	72.07 (10)	C1—P1—C2—C22	75.9 (2)
C3—P1—S1—Au1	−50.38 (12)	S1—P1—C2—C22	−168.53 (17)
C1—P1—S1—Au1	−165.21 (10)	C3—P1—C2—C21	−171.3 (2)
Br3—Au1—S1—P1	−74.36 (4)	C1—P1—C2—C21	−52.7 (3)
Br2—Au1—S1—P1	110.40 (4)	S1—P1—C2—C21	62.9 (2)
C2—P1—C1—C12	74.0 (3)	C2—P1—C3—C31	178.2 (2)
C3—P1—C1—C12	−166.9 (2)	C1—P1—C3—C31	54.9 (3)
S1—P1—C1—C12	−47.1 (2)	S1—P1—C3—C31	−57.2 (2)
C2—P1—C1—C11	−53.8 (3)	C2—P1—C3—C32	−54.6 (3)
C3—P1—C1—C11	65.3 (3)	C1—P1—C3—C32	−177.8 (2)
S1—P1—C1—C11	−174.9 (2)	S1—P1—C3—C32	70.1 (2)
C3—P1—C2—C22	−42.7 (2)		

### Tribromrido(tripropan-2-ylphosphane sulfide-κS)gold(III) (9b) Hydrogen-bond geometry (Å, º)

**Table:**

*D*—H···*A*	*D*—H	H···*A*	*D*···*A*	*D*—H···*A*
C32—H32*C*···Au1	0.98	2.76	3.473 (3)	131
C12—H12*B*···S1	0.98	2.68	3.261 (3)	118
C2—H2···Br3	1.00	2.71	3.560 (3)	143
C22—H22*B*···Au1^i^	0.98	2.98	3.551 (3)	119
C21—H21*C*···Br1^ii^	0.98	3.02	3.829 (3)	141
C3—H3···Br2^i^	1.00	2.91	3.796 (3)	148
C32—H32*A*···Br2^iii^	0.98	3.06	3.900 (3)	145
C11—H11*C*···Br2^iv^	0.98	2.91	3.661 (3)	134

Symmetry codes: (i) *x*, *y*−1, *z*; (ii) −*x*, *y*−1/2, −*z*+1/2; (iii) −*x*, −*y*+1, −*z*+1; (iv) −*x*+1, −*y*+1, −*z*+1.

### Tribromido[di-tert-butyl(propan-2-yl)phosphane sulfide-κS]gold(III) (11b) Crystal data

**Table:**

[AuBr_3_(C_11_H_25_PS)]	*Z* = 2
*M**_r_* = 657.04	*F*(000) = 612
Triclinic, *P*1	*D*_x_ = 2.451 Mg m^−^^3^
*a* = 8.6067 (8) Å	Mo *K*α radiation, λ = 0.71073 Å
*b* = 10.1161 (12) Å	Cell parameters from 29495 reflections
*c* = 11.5123 (12) Å	θ = 2.6–30.8°
α = 77.873 (10)°	µ = 15.18 mm^−^^1^
β = 70.257 (10)°	*T* = 100 K
γ = 71.867 (10)°	Block, red-brown
*V* = 890.37 (18) Å^3^	0.2 × 0.2 × 0.2 mm

### Tribromido[di-tert-butyl(propan-2-yl)phosphane sulfide-κS]gold(III) (11b) Data collection

**Table:**

Oxford Diffaction Xcalibur, Eos diffractometer	5297 independent reflections
Radiation source: Enhance (Mo) X-ray Source	5057 reflections with *I* > 2σ(*I*)
Detector resolution: 16.1419 pixels mm^-1^	*R*_int_ = 0.038
ω scan	θ_max_ = 30.9°, θ_min_ = 2.6°
Absorption correction: multi-scan (CrysAlisPro; Rigaku OD, 2020)	*h* = −12→12
*T*_min_ = 0.447, *T*_max_ = 1.000	*k* = −14→14
65025 measured reflections	*l* = −16→15

### Tribromido[di-tert-butyl(propan-2-yl)phosphane sulfide-κS]gold(III) (11b) Refinement

**Table:**

Refinement on *F*^2^	Secondary atom site location: difference Fourier map
Least-squares matrix: full	Hydrogen site location: inferred from neighbouring sites
*R*[*F*^2^ > 2σ(*F*^2^)] = 0.017	H-atom parameters constrained
*wR*(*F*^2^) = 0.036	*w* = 1/[σ^2^(*F*_o_^2^) + (0.0122*P*)^2^ + 1.1802*P*] where *P* = (*F*_o_^2^ + 2*F*_c_^2^)/3
*S* = 1.14	(Δ/σ)_max_ = 0.002
5297 reflections	Δρ_max_ = 1.08 e Å^−^^3^
163 parameters	Δρ_min_ = −1.43 e Å^−^^3^
0 restraints	Extinction correction: SHELXL-2019/3 (Sheldrick, 2015), *F*_c_^*^ = *kF*_c_[1 + 0.001 *F*_c_^2^λ^3^/sin(2θ)]^-1/4^
Primary atom site location: structure-invariant direct methods	Extinction coefficient: 0.00557 (11)

### Tribromido[di-tert-butyl(propan-2-yl)phosphane sulfide-κS]gold(III) (11b) Fractional atomic coordinates and isotropic or equivalent isotropic displacement parameters (Å^2^)

**Table:**

	*x*	*y*	*z*	*U*_iso_*/*U*_eq_	
Au1	0.46412 (2)	0.35795 (2)	0.23440 (2)	0.00889 (3)	
Br1	0.53350 (3)	0.12322 (2)	0.34392 (2)	0.01652 (5)	
Br2	0.74993 (3)	0.37590 (2)	0.20749 (2)	0.01400 (5)	
Br3	0.19529 (3)	0.32221 (2)	0.23936 (2)	0.01676 (5)	
P1	0.21504 (7)	0.72300 (6)	0.24517 (5)	0.00701 (10)	
S1	0.40792 (7)	0.58600 (6)	0.13111 (5)	0.00991 (10)	
C1	0.2527 (3)	0.7059 (2)	0.3996 (2)	0.0098 (4)	
C2	0.2271 (3)	0.8961 (2)	0.1503 (2)	0.0104 (4)	
C3	0.0095 (3)	0.6834 (2)	0.2715 (2)	0.0105 (4)	
H3	0.027169	0.582577	0.306905	0.013*	
C11	0.1481 (3)	0.8353 (2)	0.4690 (2)	0.0141 (5)	
H11A	0.188266	0.917296	0.422652	0.021*	
H11B	0.162709	0.818416	0.552175	0.021*	
H11C	0.026852	0.852351	0.476344	0.021*	
C12	0.4426 (3)	0.6869 (3)	0.3846 (2)	0.0146 (5)	
H12A	0.512084	0.606051	0.339530	0.022*	
H12B	0.459923	0.671395	0.466740	0.022*	
H12C	0.476683	0.771305	0.337815	0.022*	
C13	0.2010 (3)	0.5748 (2)	0.4781 (2)	0.0132 (4)	
H13A	0.227809	0.559330	0.556900	0.020*	
H13B	0.264247	0.493282	0.432585	0.020*	
H13C	0.077974	0.588312	0.494739	0.020*	
C21	0.2602 (3)	0.8846 (2)	0.0122 (2)	0.0147 (5)	
H21A	0.373120	0.821183	−0.019419	0.022*	
H21B	0.256084	0.977510	−0.035458	0.022*	
H21C	0.172280	0.847983	0.003539	0.022*	
C22	0.0581 (3)	1.0081 (2)	0.1944 (2)	0.0144 (5)	
H22A	0.070696	1.100103	0.150803	0.022*	
H22B	0.029741	1.009738	0.284121	0.022*	
H22C	−0.033703	0.985600	0.176468	0.022*	
C23	0.3757 (3)	0.9422 (2)	0.1601 (2)	0.0150 (5)	
H23A	0.386915	1.028408	0.104086	0.023*	
H23B	0.481998	0.868304	0.136720	0.023*	
H23C	0.353000	0.959173	0.245746	0.023*	
C31	−0.0315 (3)	0.6906 (3)	0.1500 (2)	0.0172 (5)	
H31A	−0.071273	0.788820	0.117928	0.026*	
H31B	−0.121040	0.642829	0.166014	0.026*	
H31C	0.071737	0.644795	0.088671	0.026*	
C32	−0.1474 (3)	0.7621 (3)	0.3675 (2)	0.0158 (5)	
H32A	−0.246278	0.727637	0.377566	0.024*	
H32B	−0.172299	0.862586	0.338601	0.024*	
H32C	−0.123437	0.746175	0.447427	0.024*	

### Tribromido[di-tert-butyl(propan-2-yl)phosphane sulfide-κS]gold(III) (11b) Atomic displacement parameters (Å^2^)

**Table:**

	*U*^11^	*U*^22^	*U*^33^	*U*^12^	*U*^13^	*U*^23^
Au1	0.01042 (5)	0.00647 (4)	0.00921 (4)	−0.00031 (3)	−0.00286 (3)	−0.00260 (3)
Br1	0.02234 (13)	0.00864 (10)	0.01814 (11)	−0.00152 (9)	−0.00895 (9)	0.00053 (8)
Br2	0.01064 (11)	0.01396 (10)	0.01718 (11)	−0.00019 (8)	−0.00497 (8)	−0.00448 (8)
Br3	0.01402 (11)	0.01009 (10)	0.02811 (13)	−0.00327 (8)	−0.00674 (10)	−0.00517 (9)
P1	0.0074 (2)	0.0062 (2)	0.0072 (2)	−0.00126 (19)	−0.00218 (19)	−0.00103 (18)
S1	0.0113 (3)	0.0084 (2)	0.0075 (2)	−0.00064 (19)	−0.00086 (19)	−0.00185 (18)
C1	0.0117 (10)	0.0100 (9)	0.0074 (9)	−0.0006 (8)	−0.0040 (8)	−0.0019 (7)
C2	0.0134 (11)	0.0073 (9)	0.0095 (10)	−0.0029 (8)	−0.0026 (8)	0.0001 (7)
C3	0.0100 (10)	0.0084 (9)	0.0142 (10)	−0.0033 (8)	−0.0053 (8)	0.0009 (8)
C11	0.0151 (11)	0.0132 (10)	0.0134 (11)	−0.0012 (9)	−0.0032 (9)	−0.0058 (8)
C12	0.0110 (11)	0.0185 (11)	0.0146 (11)	−0.0003 (9)	−0.0062 (9)	−0.0043 (9)
C13	0.0181 (12)	0.0103 (10)	0.0091 (10)	−0.0017 (9)	−0.0040 (8)	−0.0001 (8)
C21	0.0217 (12)	0.0119 (10)	0.0107 (10)	−0.0053 (9)	−0.0054 (9)	0.0009 (8)
C22	0.0159 (11)	0.0077 (10)	0.0180 (11)	−0.0010 (8)	−0.0053 (9)	−0.0008 (8)
C23	0.0167 (12)	0.0127 (10)	0.0170 (11)	−0.0077 (9)	−0.0040 (9)	−0.0003 (9)
C31	0.0186 (12)	0.0180 (11)	0.0210 (12)	−0.0081 (10)	−0.0125 (10)	0.0011 (9)
C32	0.0084 (11)	0.0144 (11)	0.0217 (12)	−0.0023 (9)	−0.0024 (9)	−0.0006 (9)

### Tribromido[di-tert-butyl(propan-2-yl)phosphane sulfide-κS]gold(III) (11b) Geometric parameters (Å, º)

**Table:**

Au1—S1	2.3477 (6)	C12—H12B	0.9800
Au1—Br3	2.4310 (3)	C12—H12C	0.9800
Au1—Br2	2.4330 (3)	C13—H13A	0.9800
Au1—Br1	2.4399 (4)	C13—H13B	0.9800
P1—C3	1.847 (2)	C13—H13C	0.9800
P1—C2	1.872 (2)	C21—H21A	0.9800
P1—C1	1.877 (2)	C21—H21B	0.9800
P1—S1	2.0640 (8)	C21—H21C	0.9800
C1—C12	1.537 (3)	C22—H22A	0.9800
C1—C13	1.538 (3)	C22—H22B	0.9800
C1—C11	1.543 (3)	C22—H22C	0.9800
C2—C23	1.535 (3)	C23—H23A	0.9800
C2—C21	1.541 (3)	C23—H23B	0.9800
C2—C22	1.541 (3)	C23—H23C	0.9800
C3—C31	1.536 (3)	C31—H31A	0.9800
C3—C32	1.540 (3)	C31—H31B	0.9800
C3—H3	1.0000	C31—H31C	0.9800
C11—H11A	0.9800	C32—H32A	0.9800
C11—H11B	0.9800	C32—H32B	0.9800
C11—H11C	0.9800	C32—H32C	0.9800
C12—H12A	0.9800		
			
S1—Au1—Br3	93.532 (19)	C1—C12—H12C	109.5
S1—Au1—Br2	87.959 (19)	H12A—C12—H12C	109.5
Br3—Au1—Br2	172.720 (9)	H12B—C12—H12C	109.5
S1—Au1—Br1	177.293 (16)	C1—C13—H13A	109.5
Br3—Au1—Br1	89.099 (14)	C1—C13—H13B	109.5
Br2—Au1—Br1	89.510 (14)	H13A—C13—H13B	109.5
C3—P1—C2	112.57 (10)	C1—C13—H13C	109.5
C3—P1—C1	108.53 (11)	H13A—C13—H13C	109.5
C2—P1—C1	113.56 (10)	H13B—C13—H13C	109.5
C3—P1—S1	109.09 (8)	C2—C21—H21A	109.5
C2—P1—S1	101.70 (7)	C2—C21—H21B	109.5
C1—P1—S1	111.23 (7)	H21A—C21—H21B	109.5
P1—S1—Au1	111.56 (3)	C2—C21—H21C	109.5
C12—C1—C13	108.27 (19)	H21A—C21—H21C	109.5
C12—C1—C11	107.87 (19)	H21B—C21—H21C	109.5
C13—C1—C11	109.49 (19)	C2—C22—H22A	109.5
C12—C1—P1	111.22 (15)	C2—C22—H22B	109.5
C13—C1—P1	107.83 (15)	H22A—C22—H22B	109.5
C11—C1—P1	112.09 (15)	C2—C22—H22C	109.5
C23—C2—C21	107.46 (19)	H22A—C22—H22C	109.5
C23—C2—C22	109.62 (19)	H22B—C22—H22C	109.5
C21—C2—C22	108.69 (19)	C2—C23—H23A	109.5
C23—C2—P1	110.46 (16)	C2—C23—H23B	109.5
C21—C2—P1	110.21 (15)	H23A—C23—H23B	109.5
C22—C2—P1	110.34 (16)	C2—C23—H23C	109.5
C31—C3—C32	110.81 (19)	H23A—C23—H23C	109.5
C31—C3—P1	112.74 (17)	H23B—C23—H23C	109.5
C32—C3—P1	117.05 (16)	C3—C31—H31A	109.5
C31—C3—H3	105.0	C3—C31—H31B	109.5
C32—C3—H3	105.0	H31A—C31—H31B	109.5
P1—C3—H3	105.0	C3—C31—H31C	109.5
C1—C11—H11A	109.5	H31A—C31—H31C	109.5
C1—C11—H11B	109.5	H31B—C31—H31C	109.5
H11A—C11—H11B	109.5	C3—C32—H32A	109.5
C1—C11—H11C	109.5	C3—C32—H32B	109.5
H11A—C11—H11C	109.5	H32A—C32—H32B	109.5
H11B—C11—H11C	109.5	C3—C32—H32C	109.5
C1—C12—H12A	109.5	H32A—C32—H32C	109.5
C1—C12—H12B	109.5	H32B—C32—H32C	109.5
H12A—C12—H12B	109.5		
			
C3—P1—S1—Au1	−70.82 (8)	C1—P1—C2—C23	41.03 (19)
C2—P1—S1—Au1	170.07 (8)	S1—P1—C2—C23	−78.55 (15)
C1—P1—S1—Au1	48.86 (9)	C3—P1—C2—C21	−76.56 (18)
Br3—Au1—S1—P1	70.22 (3)	C1—P1—C2—C21	159.61 (16)
Br2—Au1—S1—P1	−116.91 (3)	S1—P1—C2—C21	40.03 (17)
C3—P1—C1—C12	160.43 (16)	C3—P1—C2—C22	43.48 (19)
C2—P1—C1—C12	−73.58 (18)	C1—P1—C2—C22	−80.34 (18)
S1—P1—C1—C12	40.42 (17)	S1—P1—C2—C22	160.08 (15)
C3—P1—C1—C13	41.86 (17)	C2—P1—C3—C31	54.96 (19)
C2—P1—C1—C13	167.86 (15)	C1—P1—C3—C31	−178.47 (16)
S1—P1—C1—C13	−78.15 (15)	S1—P1—C3—C31	−57.14 (17)
C3—P1—C1—C11	−78.73 (18)	C2—P1—C3—C32	−75.32 (19)
C2—P1—C1—C11	47.3 (2)	C1—P1—C3—C32	51.24 (19)
S1—P1—C1—C11	161.26 (14)	S1—P1—C3—C32	172.58 (15)
C3—P1—C2—C23	164.85 (15)		

### Tribromido[di-tert-butyl(propan-2-yl)phosphane sulfide-κS]gold(III) (11b) Hydrogen-bond geometry (Å, º)

**Table:**

*D*—H···*A*	*D*—H	H···*A*	*D*···*A*	*D*—H···*A*
C13—H13*B*···Au1	0.98	2.69	3.607 (2)	156
C21—H21*A*···S1	0.98	2.63	3.109 (2)	110
C12—H12*A*···S1	0.98	2.89	3.417 (2)	114
C3—H3···Br3	1.00	2.71	3.546 (2)	141
C12—H12*A*···Br2	0.98	2.89	3.863 (2)	174
C13—H13*A*···Br2^i^	0.98	3.00	3.931 (2)	159

Symmetry code: (i) −*x*+1, −*y*+1, −*z*+1.

### Tribromido(tripropan-2-ylphosphane selenide-κS)gold(III) (13b) Crystal data

**Table:**

[AuBr_3_(C_9_H_21_PSe)]	*Z* = 2
*M**_r_* = 675.88	*F*(000) = 616
Triclinic, *P*1	*D*_x_ = 2.760 Mg m^−^^3^
*a* = 8.3928 (2) Å	Mo *K*α radiation, λ = 0.71073 Å
*b* = 10.1417 (4) Å	Cell parameters from 15280 reflections
*c* = 10.7567 (4) Å	θ = 2.2–30.9°
α = 94.419 (3)°	µ = 18.72 mm^−^^1^
β = 105.612 (3)°	*T* = 100 K
γ = 110.113 (3)°	Plate, dichroic pale brown / black
*V* = 813.33 (5) Å^3^	0.2 × 0.1 × 0.01 mm

### Tribromido(tripropan-2-ylphosphane selenide-κS)gold(III) (13b) Data collection

**Table:**

Oxford Diffraction Xcalibur, Eos diffractometer	4825 independent reflections
Radiation source: Enhance (Mo) X-ray Source	4278 reflections with *I* > 2σ(*I*)
Detector resolution: 16.1419 pixels mm^-1^	*R*_int_ = 0.059
ω scan	θ_max_ = 31.0°, θ_min_ = 2.2°
Absorption correction: multi-scan (CrysAlisPro; Rigaku OD, 2020)	*h* = −11→12
*T*_min_ = 0.200, *T*_max_ = 1.000	*k* = −14→14
44535 measured reflections	*l* = −14→15

### Tribromido(tripropan-2-ylphosphane selenide-κS)gold(III) (13b) Refinement

**Table:**

Refinement on *F*^2^	Primary atom site location: structure-invariant direct methods
Least-squares matrix: full	Secondary atom site location: difference Fourier map
*R*[*F*^2^ > 2σ(*F*^2^)] = 0.028	Hydrogen site location: inferred from neighbouring sites
*wR*(*F*^2^) = 0.074	H-atom parameters constrained
*S* = 1.05	*w* = 1/[σ^2^(*F*_o_^2^) + (0.0378*P*)^2^ + 1.6418*P*] where *P* = (*F*_o_^2^ + 2*F*_c_^2^)/3
4825 reflections	(Δ/σ)_max_ = 0.001
142 parameters	Δρ_max_ = 1.43 e Å^−^^3^
0 restraints	Δρ_min_ = −1.49 e Å^−^^3^

### Tribromido(tripropan-2-ylphosphane selenide-κS)gold(III) (13b) Fractional atomic coordinates and isotropic or equivalent isotropic displacement parameters (Å^2^)

**Table:**

	*x*	*y*	*z*	*U*_iso_*/*U*_eq_	
Au1	0.69422 (2)	0.57788 (2)	0.18566 (2)	0.01164 (6)	
Br1	0.83155 (6)	0.83965 (5)	0.25275 (5)	0.02209 (10)	
Br3	0.40852 (5)	0.58195 (4)	0.19223 (4)	0.01647 (9)	
Br2	0.97546 (5)	0.57176 (5)	0.17238 (5)	0.02254 (10)	
P1	0.48156 (13)	0.21791 (11)	0.26640 (10)	0.01046 (19)	
Se1	0.56222 (5)	0.31839 (4)	0.10650 (4)	0.01349 (9)	
C1	0.4341 (5)	0.0298 (4)	0.2060 (4)	0.0144 (8)	
H1	0.544743	0.026681	0.189699	0.017*	
C2	0.2956 (5)	0.2543 (4)	0.2980 (4)	0.0131 (7)	
H2	0.338155	0.360716	0.321681	0.016*	
C3	0.6650 (5)	0.2762 (4)	0.4225 (4)	0.0122 (7)	
H3	0.619362	0.219320	0.486244	0.015*	
C11	0.3991 (7)	−0.0633 (5)	0.3087 (5)	0.0241 (10)	
H11A	0.289038	−0.065939	0.325605	0.036*	
H11B	0.499672	−0.022943	0.390446	0.036*	
H11C	0.386077	−0.160493	0.275910	0.036*	
C12	0.2817 (7)	−0.0355 (5)	0.0745 (5)	0.0288 (11)	
H12A	0.278900	−0.128698	0.039686	0.043*	
H12B	0.301865	0.028362	0.011491	0.043*	
H12C	0.167277	−0.047530	0.088602	0.043*	
C21	0.1296 (5)	0.2087 (5)	0.1770 (4)	0.0176 (8)	
H21A	0.064233	0.105155	0.162763	0.026*	
H21B	0.165974	0.233390	0.099863	0.026*	
H21C	0.052253	0.258177	0.190817	0.026*	
C22	0.2491 (5)	0.1964 (5)	0.4180 (4)	0.0170 (8)	
H22A	0.159561	0.229361	0.436607	0.025*	
H22B	0.357276	0.231354	0.494636	0.025*	
H22C	0.200989	0.091614	0.398734	0.025*	
C31	0.8257 (6)	0.2440 (5)	0.4101 (5)	0.0224 (9)	
H31A	0.871399	0.294863	0.345388	0.034*	
H31B	0.789032	0.140919	0.381437	0.034*	
H31C	0.919803	0.275568	0.495506	0.034*	
C32	0.7203 (6)	0.4337 (5)	0.4804 (4)	0.0178 (8)	
H32A	0.803324	0.455971	0.569887	0.027*	
H32B	0.613970	0.452440	0.482590	0.027*	
H32C	0.778982	0.493540	0.425864	0.027*	

### Tribromido(tripropan-2-ylphosphane selenide-κS)gold(III) (13b) Atomic displacement parameters (Å^2^)

**Table:**

	*U*^11^	*U*^22^	*U*^33^	*U*^12^	*U*^13^	*U*^23^
Au1	0.01092 (8)	0.01220 (8)	0.01097 (8)	0.00286 (6)	0.00402 (6)	0.00270 (6)
Br1	0.0235 (2)	0.0131 (2)	0.0234 (2)	−0.00006 (16)	0.00726 (17)	0.00196 (16)
Br3	0.01689 (19)	0.0151 (2)	0.0233 (2)	0.00837 (15)	0.01141 (16)	0.00740 (16)
Br2	0.01151 (18)	0.0285 (2)	0.0274 (2)	0.00615 (17)	0.00769 (17)	0.00587 (19)
P1	0.0096 (4)	0.0100 (4)	0.0109 (4)	0.0028 (3)	0.0035 (3)	0.0006 (4)
Se1	0.01722 (19)	0.01294 (19)	0.01227 (19)	0.00549 (15)	0.00815 (15)	0.00196 (14)
C1	0.0175 (18)	0.0105 (18)	0.0163 (19)	0.0066 (15)	0.0058 (15)	0.0012 (15)
C2	0.0118 (17)	0.0117 (18)	0.0154 (19)	0.0044 (14)	0.0046 (14)	0.0003 (14)
C3	0.0094 (16)	0.0180 (19)	0.0080 (17)	0.0043 (14)	0.0023 (13)	0.0025 (14)
C11	0.035 (3)	0.012 (2)	0.025 (2)	0.0063 (18)	0.011 (2)	0.0037 (17)
C12	0.034 (3)	0.022 (2)	0.019 (2)	0.006 (2)	−0.0006 (19)	−0.0088 (18)
C21	0.0115 (18)	0.022 (2)	0.017 (2)	0.0045 (16)	0.0031 (15)	0.0033 (16)
C22	0.0128 (18)	0.022 (2)	0.018 (2)	0.0056 (16)	0.0086 (15)	0.0055 (16)
C31	0.0123 (19)	0.025 (2)	0.026 (2)	0.0068 (17)	0.0032 (17)	−0.0025 (19)
C32	0.0167 (19)	0.016 (2)	0.0151 (19)	0.0032 (16)	0.0022 (15)	−0.0011 (16)

### Tribromido(tripropan-2-ylphosphane selenide-κS)gold(III) (13b) Geometric parameters (Å, º)

**Table:**

Au1—Br2	2.4241 (4)	C11—H11B	0.9800
Au1—Br3	2.4321 (4)	C11—H11C	0.9800
Au1—Se1	2.4535 (4)	C12—H12A	0.9800
Au1—Br1	2.4597 (5)	C12—H12B	0.9800
P1—C2	1.831 (4)	C12—H12C	0.9800
P1—C1	1.835 (4)	C21—H21A	0.9800
P1—C3	1.835 (4)	C21—H21B	0.9800
P1—Se1	2.2085 (11)	C21—H21C	0.9800
C1—C11	1.531 (6)	C22—H22A	0.9800
C1—C12	1.541 (6)	C22—H22B	0.9800
C1—H1	1.0000	C22—H22C	0.9800
C2—C21	1.529 (5)	C31—H31A	0.9800
C2—C22	1.547 (6)	C31—H31B	0.9800
C2—H2	1.0000	C31—H31C	0.9800
C3—C32	1.528 (6)	C32—H32A	0.9800
C3—C31	1.529 (6)	C32—H32B	0.9800
C3—H3	1.0000	C32—H32C	0.9800
C11—H11A	0.9800		
			
Br2—Au1—Br3	178.373 (15)	C1—C11—H11C	109.5
Br2—Au1—Se1	87.616 (16)	H11A—C11—H11C	109.5
Br3—Au1—Se1	91.709 (14)	H11B—C11—H11C	109.5
Br2—Au1—Br1	90.566 (17)	C1—C12—H12A	109.5
Br3—Au1—Br1	90.031 (16)	C1—C12—H12B	109.5
Se1—Au1—Br1	176.522 (15)	H12A—C12—H12B	109.5
C2—P1—C1	115.50 (19)	C1—C12—H12C	109.5
C2—P1—C3	107.15 (18)	H12A—C12—H12C	109.5
C1—P1—C3	107.53 (19)	H12B—C12—H12C	109.5
C2—P1—Se1	112.81 (14)	C2—C21—H21A	109.5
C1—P1—Se1	101.08 (14)	C2—C21—H21B	109.5
C3—P1—Se1	112.75 (13)	H21A—C21—H21B	109.5
P1—Se1—Au1	107.24 (3)	C2—C21—H21C	109.5
C11—C1—C12	110.4 (4)	H21A—C21—H21C	109.5
C11—C1—P1	112.1 (3)	H21B—C21—H21C	109.5
C12—C1—P1	113.9 (3)	C2—C22—H22A	109.5
C11—C1—H1	106.6	C2—C22—H22B	109.5
C12—C1—H1	106.6	H22A—C22—H22B	109.5
P1—C1—H1	106.6	C2—C22—H22C	109.5
C21—C2—C22	111.7 (3)	H22A—C22—H22C	109.5
C21—C2—P1	113.5 (3)	H22B—C22—H22C	109.5
C22—C2—P1	113.3 (3)	C3—C31—H31A	109.5
C21—C2—H2	105.8	C3—C31—H31B	109.5
C22—C2—H2	105.8	H31A—C31—H31B	109.5
P1—C2—H2	105.8	C3—C31—H31C	109.5
C32—C3—C31	111.2 (3)	H31A—C31—H31C	109.5
C32—C3—P1	112.2 (3)	H31B—C31—H31C	109.5
C31—C3—P1	112.0 (3)	C3—C32—H32A	109.5
C32—C3—H3	107.0	C3—C32—H32B	109.5
C31—C3—H3	107.0	H32A—C32—H32B	109.5
P1—C3—H3	107.0	C3—C32—H32C	109.5
C1—C11—H11A	109.5	H32A—C32—H32C	109.5
C1—C11—H11B	109.5	H32B—C32—H32C	109.5
H11A—C11—H11B	109.5		
			
C2—P1—Se1—Au1	67.97 (14)	C3—P1—C2—C21	−178.9 (3)
C1—P1—Se1—Au1	−168.10 (13)	Se1—P1—C2—C21	56.5 (3)
C3—P1—Se1—Au1	−53.58 (15)	C1—P1—C2—C22	69.7 (3)
Br2—Au1—Se1—P1	114.96 (3)	C3—P1—C2—C22	−50.1 (3)
Br3—Au1—Se1—P1	−66.53 (3)	Se1—P1—C2—C22	−174.7 (2)
C2—P1—C1—C11	−65.9 (4)	C2—P1—C3—C32	−57.0 (3)
C3—P1—C1—C11	53.7 (4)	C1—P1—C3—C32	178.3 (3)
Se1—P1—C1—C11	172.0 (3)	Se1—P1—C3—C32	67.7 (3)
C2—P1—C1—C12	60.4 (4)	C2—P1—C3—C31	177.1 (3)
C3—P1—C1—C12	179.9 (3)	C1—P1—C3—C31	52.3 (4)
Se1—P1—C1—C12	−61.7 (3)	Se1—P1—C3—C31	−58.2 (3)
C1—P1—C2—C21	−59.1 (4)		

### Tribromido(tripropan-2-ylphosphane selenide-κS)gold(III) (13b) Hydrogen-bond geometry (Å, º)

**Table:**

*D*—H···*A*	*D*—H	H···*A*	*D*···*A*	*D*—H···*A*
C32—H32*C*···Au1	0.98	2.77	3.578 (5)	140
C12—H12*B*···Se1	0.98	2.90	3.479 (5)	119
C2—H2···Br3	1.00	2.71	3.497 (4)	136
C11—H11*C*···Br3^i^	0.98	2.78	3.752 (5)	171
C32—H32*A*···Br2^ii^	0.98	2.99	3.933 (4)	163

Symmetry codes: (i) *x*, *y*−1, *z*; (ii) −*x*+2, −*y*+1, −*z*+1.

### Tribromido[di-tert-butyl(propan-2-yl)phosphane selenide-κS]gold(III) (15b) Crystal data

**Table:**

[AuBr_3_(C_11_H_25_PSe)]	*Z* = 2
*M**_r_* = 703.93	*F*(000) = 648
Triclinic, *P*1	*D*_x_ = 2.586 Mg m^−^^3^
*a* = 8.6000 (5) Å	Mo *K*α radiation, λ = 0.71073 Å
*b* = 10.2045 (7) Å	Cell parameters from 9711 reflections
*c* = 11.5987 (7) Å	θ = 2.6–30.7°
α = 77.475 (6)°	µ = 16.85 mm^−^^1^
β = 69.764 (6)°	*T* = 100 K
γ = 72.601 (6)°	Lath, dichroic black / orange
*V* = 904.02 (11) Å^3^	0.2 × 0.06 × 0.04 mm

### Tribromido[di-tert-butyl(propan-2-yl)phosphane selenide-κS]gold(III) (15b) Data collection

**Table:**

Oxford Diffraction Xcalibur, Eos diffractometer	5282 independent reflections
Radiation source: Enhance (Mo) X-ray Source	4719 reflections with *I* > 2σ(*I*)
Detector resolution: 16.1419 pixels mm^-1^	*R*_int_ = 0.033
ω scans	θ_max_ = 30.8°, θ_min_ = 2.6°
Absorption correction: multi-scan (CrysAlisPro; Rigaku OD, 2020)	*h* = −12→12
*T*_min_ = 0.376, *T*_max_ = 1.000	*k* = −14→14
25566 measured reflections	*l* = −16→16

### Tribromido[di-tert-butyl(propan-2-yl)phosphane selenide-κS]gold(III) (15b) Refinement

**Table:**

Refinement on *F*^2^	Hydrogen site location: inferred from neighbouring sites
Least-squares matrix: full	H-atom parameters constrained
*R*[*F*^2^ > 2σ(*F*^2^)] = 0.020	*w* = 1/[σ^2^(*F*_o_^2^) + (0.011*P*)^2^ + 0.1908*P*] where *P* = (*F*_o_^2^ + 2*F*_c_^2^)/3
*wR*(*F*^2^) = 0.036	(Δ/σ)_max_ = 0.001
*S* = 1.05	Δρ_max_ = 1.04 e Å^−^^3^
5282 reflections	Δρ_min_ = −0.80 e Å^−^^3^
163 parameters	Extinction correction: SHELXL-2019/3 (Sheldrick, 2015), *F*_c_^*^ = *kF*_c_[1 + 0.001 *F*_c_^2^λ^3^/sin(2θ)]^-1/4^
0 restraints	Extinction coefficient: 0.00115 (6)
Primary atom site location: structure-invariant direct methods	

### Tribromido[di-tert-butyl(propan-2-yl)phosphane selenide-κS]gold(III) (15b) Fractional atomic coordinates and isotropic or equivalent isotropic displacement parameters (Å^2^)

**Table:**

	*x*	*y*	*z*	*U*_iso_*/*U*_eq_	
Au1	0.47180 (2)	0.35576 (2)	0.23406 (2)	0.01020 (3)	
Br1	0.53346 (3)	0.12201 (3)	0.34571 (2)	0.01753 (6)	
Br2	0.75742 (3)	0.37440 (3)	0.20826 (2)	0.01554 (6)	
Br3	0.19859 (3)	0.32636 (3)	0.24007 (3)	0.01777 (6)	
P1	0.21219 (8)	0.73040 (7)	0.24676 (6)	0.00837 (12)	
Se1	0.42295 (3)	0.59094 (3)	0.12166 (2)	0.01100 (5)	
C1	0.2529 (3)	0.7103 (3)	0.3998 (2)	0.0105 (5)	
C2	0.2206 (3)	0.9043 (3)	0.1533 (2)	0.0121 (5)	
C3	0.0086 (3)	0.6854 (3)	0.2742 (2)	0.0120 (5)	
H3	0.028316	0.585158	0.308624	0.014*	
C11	0.1482 (3)	0.8360 (3)	0.4709 (2)	0.0144 (5)	
H11A	0.185931	0.919044	0.424127	0.022*	
H11B	0.165555	0.818824	0.552543	0.022*	
H11C	0.026587	0.849945	0.481054	0.022*	
C12	0.4427 (3)	0.6939 (3)	0.3826 (2)	0.0145 (5)	
H12A	0.511328	0.612298	0.340854	0.022*	
H12B	0.459941	0.682562	0.463805	0.022*	
H12C	0.478089	0.776527	0.332290	0.022*	
C13	0.2029 (3)	0.5779 (3)	0.4767 (2)	0.0138 (5)	
H13A	0.234962	0.558998	0.553047	0.021*	
H13B	0.262708	0.499890	0.428497	0.021*	
H13C	0.079221	0.590537	0.497531	0.021*	
C21	0.2509 (3)	0.8956 (3)	0.0155 (2)	0.0152 (5)	
H21A	0.365047	0.836963	−0.018616	0.023*	
H21B	0.242429	0.988685	−0.030855	0.023*	
H21C	0.164595	0.855704	0.008515	0.023*	
C22	0.0512 (3)	1.0113 (3)	0.2003 (2)	0.0159 (5)	
H22A	0.060599	1.102954	0.154970	0.024*	
H22B	0.026812	1.013658	0.288936	0.024*	
H22C	−0.041513	0.985354	0.186872	0.024*	
C23	0.3687 (3)	0.9527 (3)	0.1604 (2)	0.0152 (5)	
H23A	0.376770	1.040134	0.106096	0.023*	
H23B	0.475735	0.882506	0.133773	0.023*	
H23C	0.348598	0.966518	0.245897	0.023*	
C31	−0.0347 (3)	0.6930 (3)	0.1546 (2)	0.0181 (6)	
H31A	−0.073019	0.790210	0.122437	0.027*	
H31B	−0.125850	0.645820	0.172381	0.027*	
H31C	0.067180	0.647990	0.092845	0.027*	
C32	−0.1478 (3)	0.7586 (3)	0.3717 (2)	0.0179 (6)	
H32A	−0.246013	0.722341	0.382432	0.027*	
H32B	−0.174112	0.858328	0.344102	0.027*	
H32C	−0.122923	0.741852	0.450686	0.027*	

### Tribromido[di-tert-butyl(propan-2-yl)phosphane selenide-κS]gold(III) (15b) Atomic displacement parameters (Å^2^)

**Table:**

	*U*^11^	*U*^22^	*U*^33^	*U*^12^	*U*^13^	*U*^23^
Au1	0.01150 (5)	0.00826 (5)	0.01035 (5)	−0.00006 (4)	−0.00365 (3)	−0.00294 (3)
Br1	0.02268 (13)	0.01024 (13)	0.01990 (14)	−0.00161 (11)	−0.01002 (11)	0.00022 (10)
Br2	0.01258 (12)	0.01528 (13)	0.01902 (13)	−0.00035 (10)	−0.00666 (10)	−0.00385 (11)
Br3	0.01498 (12)	0.01201 (13)	0.02826 (15)	−0.00277 (10)	−0.00777 (11)	−0.00519 (11)
P1	0.0086 (3)	0.0075 (3)	0.0088 (3)	−0.0009 (2)	−0.0030 (2)	−0.0015 (2)
Se1	0.01263 (12)	0.00941 (12)	0.00860 (11)	0.00004 (10)	−0.00190 (9)	−0.00236 (9)
C1	0.0109 (11)	0.0105 (12)	0.0103 (12)	−0.0004 (10)	−0.0038 (9)	−0.0039 (10)
C2	0.0133 (12)	0.0104 (12)	0.0129 (12)	−0.0028 (10)	−0.0046 (10)	−0.0015 (10)
C3	0.0133 (12)	0.0088 (12)	0.0159 (13)	−0.0028 (10)	−0.0070 (10)	−0.0009 (10)
C11	0.0179 (13)	0.0122 (13)	0.0122 (12)	−0.0002 (10)	−0.0044 (10)	−0.0047 (10)
C12	0.0117 (12)	0.0202 (14)	0.0136 (13)	0.0006 (11)	−0.0080 (10)	−0.0056 (11)
C13	0.0176 (13)	0.0111 (13)	0.0109 (12)	0.0008 (10)	−0.0062 (10)	−0.0009 (10)
C21	0.0196 (13)	0.0131 (13)	0.0112 (12)	−0.0032 (11)	−0.0043 (10)	0.0004 (10)
C22	0.0158 (12)	0.0114 (13)	0.0184 (13)	−0.0003 (10)	−0.0049 (10)	−0.0023 (11)
C23	0.0180 (13)	0.0126 (13)	0.0157 (13)	−0.0064 (11)	−0.0041 (10)	−0.0010 (10)
C31	0.0173 (13)	0.0194 (15)	0.0218 (14)	−0.0052 (11)	−0.0116 (11)	−0.0005 (12)
C32	0.0098 (12)	0.0177 (14)	0.0227 (14)	−0.0027 (11)	−0.0029 (10)	0.0002 (12)

### Tribromido[di-tert-butyl(propan-2-yl)phosphane selenide-κS]gold(III) (15b) Geometric parameters (Å, º)

**Table:**

Au1—Br2	2.4302 (3)	C12—H12B	0.9800
Au1—Br3	2.4320 (3)	C12—H12C	0.9800
Au1—Br1	2.4549 (3)	C13—H13A	0.9800
Au1—Se1	2.4606 (3)	C13—H13B	0.9800
P1—C3	1.847 (2)	C13—H13C	0.9800
P1—C2	1.875 (3)	C21—H21A	0.9800
P1—C1	1.883 (3)	C21—H21B	0.9800
P1—Se1	2.2247 (7)	C21—H21C	0.9800
C1—C12	1.536 (3)	C22—H22A	0.9800
C1—C13	1.538 (3)	C22—H22B	0.9800
C1—C11	1.546 (3)	C22—H22C	0.9800
C2—C23	1.530 (3)	C23—H23A	0.9800
C2—C22	1.539 (3)	C23—H23B	0.9800
C2—C21	1.546 (3)	C23—H23C	0.9800
C3—C32	1.536 (3)	C31—H31A	0.9800
C3—C31	1.536 (3)	C31—H31B	0.9800
C3—H3	1.0000	C31—H31C	0.9800
C11—H11A	0.9800	C32—H32A	0.9800
C11—H11B	0.9800	C32—H32B	0.9800
C11—H11C	0.9800	C32—H32C	0.9800
C12—H12A	0.9800		
			
Br2—Au1—Br3	174.078 (10)	C1—C12—H12C	109.5
Br2—Au1—Br1	90.066 (11)	H12A—C12—H12C	109.5
Br3—Au1—Br1	89.729 (12)	H12B—C12—H12C	109.5
Br2—Au1—Se1	87.317 (11)	C1—C13—H13A	109.5
Br3—Au1—Se1	92.892 (11)	C1—C13—H13B	109.5
Br1—Au1—Se1	177.377 (9)	H13A—C13—H13B	109.5
C3—P1—C2	112.60 (11)	C1—C13—H13C	109.5
C3—P1—C1	108.84 (11)	H13A—C13—H13C	109.5
C2—P1—C1	114.02 (11)	H13B—C13—H13C	109.5
C3—P1—Se1	109.35 (9)	C2—C21—H21A	109.5
C2—P1—Se1	101.46 (8)	C2—C21—H21B	109.5
C1—P1—Se1	110.34 (8)	H21A—C21—H21B	109.5
P1—Se1—Au1	108.81 (2)	C2—C21—H21C	109.5
C12—C1—C13	108.1 (2)	H21A—C21—H21C	109.5
C12—C1—C11	108.1 (2)	H21B—C21—H21C	109.5
C13—C1—C11	109.8 (2)	C2—C22—H22A	109.5
C12—C1—P1	111.46 (16)	C2—C22—H22B	109.5
C13—C1—P1	107.66 (17)	H22A—C22—H22B	109.5
C11—C1—P1	111.76 (17)	C2—C22—H22C	109.5
C23—C2—C22	109.7 (2)	H22A—C22—H22C	109.5
C23—C2—C21	107.4 (2)	H22B—C22—H22C	109.5
C22—C2—C21	108.6 (2)	C2—C23—H23A	109.5
C23—C2—P1	110.52 (17)	C2—C23—H23B	109.5
C22—C2—P1	110.37 (17)	H23A—C23—H23B	109.5
C21—C2—P1	110.18 (18)	C2—C23—H23C	109.5
C32—C3—C31	110.6 (2)	H23A—C23—H23C	109.5
C32—C3—P1	116.61 (18)	H23B—C23—H23C	109.5
C31—C3—P1	113.40 (17)	C3—C31—H31A	109.5
C32—C3—H3	105.0	C3—C31—H31B	109.5
C31—C3—H3	105.0	H31A—C31—H31B	109.5
P1—C3—H3	105.0	C3—C31—H31C	109.5
C1—C11—H11A	109.5	H31A—C31—H31C	109.5
C1—C11—H11B	109.5	H31B—C31—H31C	109.5
H11A—C11—H11B	109.5	C3—C32—H32A	109.5
C1—C11—H11C	109.5	C3—C32—H32B	109.5
H11A—C11—H11C	109.5	H32A—C32—H32B	109.5
H11B—C11—H11C	109.5	C3—C32—H32C	109.5
C1—C12—H12A	109.5	H32A—C32—H32C	109.5
C1—C12—H12B	109.5	H32B—C32—H32C	109.5
H12A—C12—H12B	109.5		
			
C3—P1—Se1—Au1	−69.41 (9)	C1—P1—C2—C23	41.2 (2)
C2—P1—Se1—Au1	171.47 (8)	Se1—P1—C2—C23	−77.43 (17)
C1—P1—Se1—Au1	50.27 (9)	C3—P1—C2—C22	44.3 (2)
Br2—Au1—Se1—P1	−117.58 (2)	C1—P1—C2—C22	−80.3 (2)
Br3—Au1—Se1—P1	68.34 (2)	Se1—P1—C2—C22	161.06 (16)
C3—P1—C1—C12	159.36 (17)	C3—P1—C2—C21	−75.64 (19)
C2—P1—C1—C12	−74.0 (2)	C1—P1—C2—C21	159.73 (16)
Se1—P1—C1—C12	39.38 (19)	Se1—P1—C2—C21	41.14 (17)
C3—P1—C1—C13	41.02 (19)	C2—P1—C3—C32	−75.6 (2)
C2—P1—C1—C13	167.64 (15)	C1—P1—C3—C32	51.9 (2)
Se1—P1—C1—C13	−78.96 (16)	Se1—P1—C3—C32	172.46 (16)
C3—P1—C1—C11	−79.61 (19)	C2—P1—C3—C31	54.6 (2)
C2—P1—C1—C11	47.0 (2)	C1—P1—C3—C31	−177.92 (18)
Se1—P1—C1—C11	160.40 (15)	Se1—P1—C3—C31	−57.3 (2)
C3—P1—C2—C23	165.79 (17)		

### Tribromido[di-tert-butyl(propan-2-yl)phosphane selenide-κS]gold(III) (15b) Hydrogen-bond geometry (Å, º)

**Table:**

*D*—H···*A*	*D*—H	H···*A*	*D*···*A*	*D*—H···*A*
C13—H13*B*···Au1	0.98	2.74	3.679 (3)	160
C21—H21*A*···Se1	0.98	2.69	3.197 (3)	113
C12—H12*A*···Se1	0.98	2.96	3.484 (3)	115
C3—H3···Br3	1.00	2.75	3.585 (3)	142
C12—H12*A*···Br2	0.98	3.01	3.980 (3)	173
C13—H13*A*···Br2^i^	0.98	3.02	3.929 (3)	156

Symmetry code: (i) −*x*+1, −*y*+1, −*z*+1.
